# Hidden Markov induced Dynamic Bayesian Network for recovering time evolving gene regulatory networks

**DOI:** 10.1038/srep17841

**Published:** 2015-12-18

**Authors:** Shijia Zhu, Yadong Wang

**Affiliations:** 1 School of Computer Science and Technology, Harbin Institute of Technology, Harbin, Heilongjiang, 150001, China

## Abstract

Dynamic Bayesian Networks (DBN) have been widely used to recover gene regulatory relationships from time-series data in computational systems biology. Its standard assumption is ‘stationarity’, and therefore, several research efforts have been recently proposed to relax this restriction. However, those methods suffer from three challenges: long running time, low accuracy and reliance on parameter settings. To address these problems, we propose a novel non-stationary DBN model by extending each hidden node of Hidden Markov Model into a DBN (called HMDBN), which properly handles the underlying time-evolving networks. Correspondingly, an improved structural EM algorithm is proposed to learn the HMDBN. It dramatically reduces searching space, thereby substantially improving computational efficiency. Additionally, we derived a novel generalized Bayesian Information Criterion under the non-stationary assumption (called BWBIC), which can help significantly improve the reconstruction accuracy and largely reduce over-fitting. Moreover, the re-estimation formulas for all parameters of our model are derived, enabling us to avoid reliance on parameter settings. Compared to the state-of-the-art methods, the experimental evaluation of our proposed method on both synthetic and real biological data demonstrates more stably high prediction accuracy and significantly improved computation efficiency, even with no prior knowledge and parameter settings.

Among diverse tools available for analyzing temporal sequences, Dynamic Bayesian Network (DBN) has been one of the most widely used to infer regulatory relationships in systems biology. The standard assumption underlying DBN is stationarity, that is, the structure and parameters of DBN are fixed over time. However, this hypothesis is too restrictive and does not hold for many real biological problems. For instance, gene regulatory relationships and signal transduction processes in the cell are usually adaptive and change due to the environmental stimuli and growth phases, such as immune responses, cancer progression, and developmental processes.

There have been various efforts to relax the stationary assumption for *undirected* graphical models, such as Markov Chain Monte Carlo (MCMC) and convex optimization-based Gaussian graphical models^1^,^2^, and especially, the widely used *l1*-norm regression-based time-varying networks^3^,^4^,^5^,^6^,^7^. While these methods are all promising, their restriction is that the undirected graphical models lack semantic interpretability when compared to the directed probabilistic graphical model DBN. The directed edges in DBN bear a natural causal implication and are more likely to suggest regulatory relations.

Relaxing the stationary restriction in DBNs is a very recent research topic^8^,^9^,^10^,^11^,^12^,^13^. These approaches are all based on a combination of DBN with a multiple change-point process, and the application of a Bayesian inference scheme via Reversible Jump Markov Chain Monte Carlo (RJMCMC) sampling. To be specific, the works^8^,^9^ proposed a discrete non-stationary DBN, which allows for different structures in different segments of the time series, with global change points for all variables. The works^10^,^11^ proposed a continuous inhomogeneous DBN, which assumes a fixed network structure and only allows the parameters to vary with time. The works^12^,^13^ proposed an alternative continuous regression-based time-varying DBN with *node-specific* change points, that is, network structures associated with different nodes are allowed to change with time in different ways. These extended DBN models, however, still have obvious limitations, leaving room for further methodological innovation.

## Running time

These works employ RJMCMC sampling to infer the non-stationary network. The primary disadvantage of sampling methods in comparison to search methods is that they often take much longer before converging on accurate results. Additionally, it is very important but difficult for sampling technique to identify when the algorithm converges. User experience is sometimes required to specify a suitable iteration step based on the complexity of the problem.

## Parameter settings

In these methods, different probabilistic distributions are assumed to penalize the number of change points, such as exponential, negative binomial and Poisson distributions. Various parameters are introduced accordingly. However, these works did not infer all parameters from data, with some of them set manually. The prediction under different parameter settings might change largely, thereby resulting in inference uncertainty.

## Scoring criteria

The relaxation of stationary hypothesis for DBN leads to a highly flexible model. This might lead to over-fitting or inflated inference uncertainty, especially when the subsequent transition times are close together, and the network structures must be inferred from short time series segments. To address this problem, previous works have proposed to couple information sequentially^8^,^9^,^14^,^15^ or globally^16^,^17^ by assuming similar parameters for networks on different time segments. However, the traditional metrics for evaluating a stationary DBN, e.g. Bayesian-Dirichlet equivalent (BDe) metric^18^ and Bayesian Information Criteria (BIC)^19^, only use the data in each time segment to separately evaluate each individual network. These metrics cannot benefit from information sharing among different time segments. The works^9^,^11^ extend the traditional BDe and BGe scores for non-stationary networks in discrete and continuous conditions, called nsBDe and cpBGe, respectively. However, they are still simple applications of traditional BDe and BGe to stationary DBNs on each time segment.

In this paper, we propose a novel node-specific, non-stationary DBN model by extending each hidden node of Hidden Markov Model (HMM) into a DBN that is capable of modeling the underlying time-evolving network structures. Next, we propose an improved Structural Expectation Maximization (SEM) algorithm to learn a HMDBN model from a time-series dataset. On the basis of SEM, we first derive the re-estimation formulas for all parameters of our model by maximizing the objective function of SEM; meanwhile, we derived a novel generalized BIC under non-stationary assumption; finally, we propose a heuristic time-efficient approach to reduce searching space of the SEM algorithm. Compared to some recent state-of-art methods in the literatures, the experimental evaluation of our proposed method on both synthetic and real biological data demonstrates more stably high prediction accuracy and significantly improved computation speed, even without prior knowledge and parameter settings.

Our approach has the following attractive contributions:

## Novel non-stationary DBN model

Two well-studied methods, HMM and DBN, are combined to address real-biological problems. The existing research achievements for these two models, such as the well-known Viterbi algorithm, Baum-Welch algorithm^20^ and score-based greedy climbing algorithm, motivate us to propose a search-based method to decode the transition time as well as learn the parameters and network structures.

## Estimation for all parameters from data

The derived re-estimation formulas for all parameters enable us to infer all parameters from data.

## Novel reasonable non-stationary BIC metric

This novel metric can benefit from the information sharing among networks on different time segments. This sharing allows our proposed metric to more reasonably evaluate a candidate non-stationary network. Compared to traditional metrics, it can greatly improve the prediction accuracy and reduce over-fitting.

## Time-efficient heuristic searching method

A heuristic approach is proposed to reduce the searching space for a non-stationary DBN to the identical one for a stationary DBN, thereby substantially improving the computation speed.

## Methods

### DBN

Consider a finite set 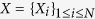 of *N* variables (nodes), and let the lowercase letter 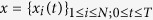 represent the realization of variables *X*_*1:N*_ across *T* + *1* time points. Note that, we specify that time points start at zero.

DBNs are flexible models for representing probabilistic relationships between interacting variables (nodes), *X*_1:*N*_, via a directed graph *G*. An edge pointed from *X*_*j*_ to *X*_*i*_ encodes the conditional dependence of *x*_*i*_*(t)* on *x*_*j*_*(t−1)*. The parent node set of *X*_*i*_ in *G*, denoted by *G*_*i*_, is the set of all nodes from which an edge points to node *X*_*i*_ in *G*. In DBN, a variable is conditionally independent of its non-descendants given all its parents (called *conditional independence*), and accordingly, the joint probability over all variables *X* can be factorized into the following chain rule:


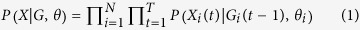


where 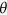 are the network parameters, composed of node-specific subvectors 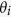, which specify the local conditional distributions in the factorization.

The common approach of learning a DBN structure is to first give a scoring function that evaluates each network with respect to the training data. BDe and BIC scores are two most widely used scoring metrics. Next, given a metric, a strategy for finding the best network must be decided. Heuristic search methods (e.g., greedy hill-climbing) can be used to find the best network topology. Alternatively, sampling methods can be used to estimate posterior probabilities over all networks. If the best network is all we need, heuristic search methods typically find it more quickly than sampling methods.

### HMM

A *HMM* can be presented as the simple example of DBN. It is a graphical probabilistic model that models the observed inputs are generated by unobserved hidden states, using transitions between hidden states to model their temporal/spatial relationships. The HMM model is characterized by its transition probability, emission probability and prior distribution. Standard HMM algorithms for path inference and parameter learning include Viterbi algorithm^20^, Baum-Welch algorithm^21^ and Viterbi training algorithm^22^. The Viterbi algorithm is a dynamic programming algorithm for identifying the optimal hidden state sequence. Baum-Welch algorithm is an Expectation Maximization (EM) algorithm for finding parameters that locally maximize the likelihood given a HMM model, and Viterbi Training algorithm (as opposed to the “Viterbi algorithm”) is the equivalent hard-updates learning algorithm, which is much faster but also less precise, because it assumes the data at each time point is generated from a single hidden state.

### The HMDBN Model

The traditional stationary *DBN* is too restricted to describe the behavior of a network topology evolving over time; in contrast, *HMM* captures the transitions among different states, although it cannot capture the conditional dependencies among variables. Motivated by such observations, we sought to combine the advantages of DBN and *HMM*, and propose a novel non-stationary *DBN*, called *Hidden Markov induced Dynamic Bayesian Network* (*HMDBN*). The *HMDBN* extends each hidden node of the traditional HMM into a hidden DBN (called *hidden graph*), and develops the transition between nodes to describe the transition between network structures. It models that multiple observed inputs 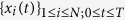 are generated by the unobserved hidden graphs. Correspondingly, the conditional probability of observation data with respect to each *DBN* and the conditional probability between *DBNs* are used as the *emission probability* and the *transition probability* for *HMDBN*, respectively. This extension integrates the description of conditional dependencies among variables (nodes) together with the network structure evolution over the time.

Next, we give the definition of *HMDBN*. We specify the node-specific attributes for *HMDBN*, and later, we will show that, similar to DBN, the likelihood for *HMDBN* can be also factorized into the product of likelihood for each node-specific *HMDBN.*

*HMDBN* is a 4-tuple 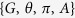:

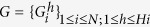
 represent hidden graphs; 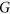 is a set of DBNs for *N* variables (nodes), *X*_*1…N*_ ; we define *H*_*i*_ to be the number of configurations of parent node sets, which *X*_*i*_ may change over time; 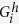 represents the *h-th* configuration of parents of *X*_*i*_;
- let 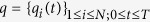 be the hidden graph sequence that generates the observation *x(1:T)*, with 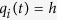 representing that *X*_*i*_*(t)* takes the parents, 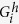;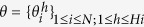 represent network parameters; in this paper, 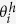 is assumed to be independent and multinomially distributed, and thus, 
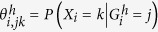
, with 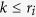 and 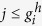, parameterizes the conditional probability of variable *X*_*i*_ given its parents 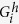, where *r*_*i*_ and 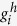 are the numbers of discrete states of *X*_*i*_ and its parent set 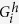, respectively;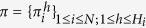, with 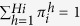, represent the prior distribution, where 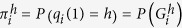 is the distribution for the initial hidden graph 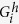;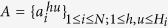, with 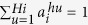, represent the transition matrix, where 
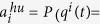
 
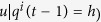
 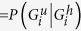 represents the transition probability from hidden graph 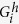 to 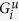;
- Finally, the conditional probability of observation *x*_*i*_*(t)* given hidden graph 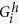 is used to represent the emission probability, i.e., 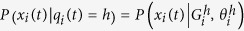.

The likelihood function of *HMDBN* for all variables *X*_*1…N*_ takes the following form:


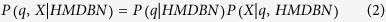


The transition between hidden graphs is assumed to follow the 1^st^ order Markov chain. Moreover the current observation is assumed to be statistically independent of the previous observations, i.e., 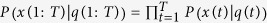. Accordingly, it is easy to prove that


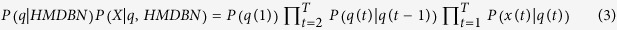


Additionally, we assume that hidden state 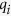 for variable *X*_*i*_ is statistically independent of those for other variables. Meanwhile, the hidden graph, which is also a DBN, follows the assumption of conditional independence. So, the above formula becomes


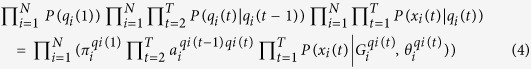


**Figure 1:**
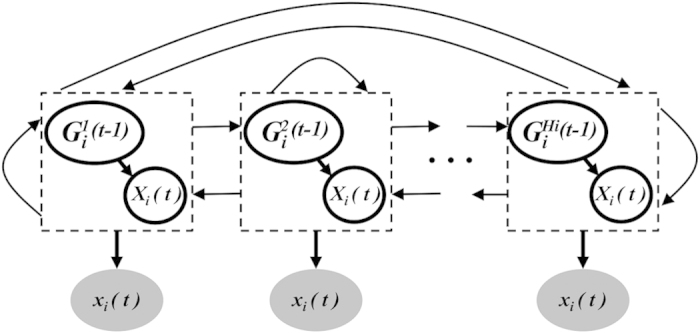
The graphical illustration of *HMDBN*_*i*_ for variable *X*_*i*_. Shaded nodes represent observed states; here, we use lowercase letter xi to represent realization of variable *X*_*i*_. The graphs in rectangles denote the latent DBN structures over time,where the blank nodes *X* represent the variables (nodes) in DBN, *G*_*i*_^*1:Hi*^ represent the parent node set for variable *X*_*i*_, and *(t)* represents the time point.

Thus, we decomposed the likelihood function for all variables into a product of terms, where each term depends only on the choice of parents for a particular variable and the relevant node-specific properties (e.g., network parameters and transition probabilities). This allows for a modular evaluation of a candidate non-stationary network and of all local changes to it. Later, we use *HMDBN*_*i*_ to represent the set of 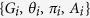, which locally models the non-stationary sub-network for variable *X*_*i*_. Figure 1 presents the graphical illustration of a non-stationary sub-network *HMDBN*_*i*_. The *HMDBNs* for other variables are similar to *HMDBN*_*i*_. In *HMDBN*_*i*_, the graph in each rectangle represents a sub-network, which only models the conditional dependencies of variable *X*_*i*_ and its parents *G*_*i*_. The digits in the parentheses represent time points. For simplicity, contemporaneous edges in DBN are not allowed, i.e. the conditional dependencies are modeled only between *X*_*i*_ at time *t* and its parents at previous time *t* − *1*. The shaded nodes represent the observable states. Each hidden graph associates with an observable state. The full connection between hidden graphs indicates that the hidden graphs could transit from one state at the current time point to any state at the next time point with certain probabilities. For one variable, the time-points are referred to as *transition times*, for which the regulatory inputs of the variables change. The *HMDBN* is node-specific, which allows different transition times and hidden graphs for each non-stationary sub-network *HMDBN*_*i*_.

### Learning HMDBN

For traditional HMM, a well-known EM algorithm (Baum-Welch algorithm) is used to learn parameters, while for *HMDBN*, we need to infer the number of hidden graphs *H*, estimate the transition probabilities *A*, and recover the hidden graph structure *G*. Therefore, we turn to the SEM algorithm^23^. It combines the standard EM algorithm, which optimizes parameters, with structure search for model selection.

#### BWBIC score

The E-step of the SEM algorithm searches for the value of the *Q* function for *HMDBN* models, i.e. the expected value of 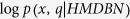
with respect to the current estimate of *HMDBN*, represented by 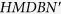, where 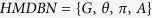. The *Q* function can be written as follows (details refer to Supplementary File 1):


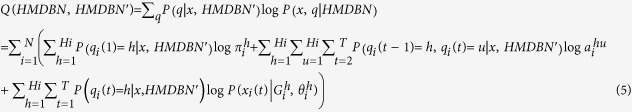


The first two terms in parenthesis of Eq. (5) can be maximized by solving with respect to 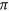 and *A*. Then, we can get their re-estimates:


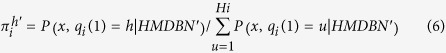



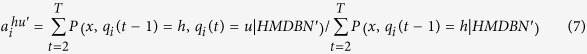


Next, to obtain 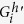, which optimizes the third term, we first get the marginal likelihood for each hidden graph 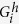, and then, solve the following optimization problem,


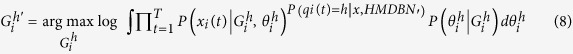


This term, however, is very hard to maximize directly with respect to the network structure *G* in the M-step, since it is a *NP-complete* problem^24^. To address this problem, the SEM algorithm does not maximize the marginal likelihood at each iteration, but attempts to find a better network structure that progressively improves the marginal likelihood. This is a generalized EM algorithm, which still guarantees to converge to a local maximum. Thus, the next question is how to calculate the logarithmic marginal likelihood with respect to a network structure in formula (8). To get an efficient way, we derived an asymptotic approximation to this integral using a large-sample approximation technique, Laplace approximation. The basic idea is that, as the sample size increases, the above integral can be approximated around its point of maximum using a multivariate Gaussian distribution. Detailed derivation is given in Supplementary File 1. Thus, the logarithmic marginal likelihood in formula (8) is approximated to the following formula:


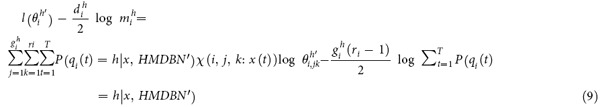


where


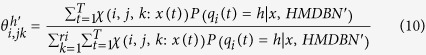


In the above formulation, 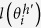 represents the logarithmic marginal likelihood of 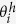 evaluated at 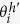; 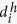 represents the number of independent parameters in 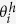; 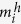 represents the expected sample size; 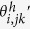 is the maximum likelihood estimate for parameter 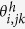; 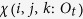 is a Kronecker delta, representing the number of times *X*_*i*_ takes on the value *k* when its parents take on the value *j* in observation *x* at time point *t*. 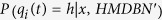 represents the probability that observation 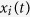 is generated by the hidden graph 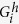. It can be calculated using the Baum-Welch algorithm (here, we do not give details).

This approximation is an extended BIC score by incorporating Baum-Welch algorithm, so, we call it Baum-Welch BIC (BWBIC). BWBIC is a generalized form of BIC under the non-stationary assumption. Its first term is the maximized value of the likelihood function; the second term is the penalty for model complexity, including the edge number and the hidden graph number. Note that, all the re-estimates for 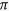, 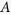 and the BWBIC score can be factorized into terms of *HMDBN* for each variable *X*_*i*_. This fact allows for a modular evaluation of a candidate non-stationary network and of all local changes to it. For instance, the evaluation of a particular change (e.g., adding an edge from *X*_*i*_ to *X*_*j*_) remains the same after changing a different part of the network (e.g., removing an edge from *X*_*i*_ to *X*_*k*_). Thus, after making one change, we do not need to re-estimate the parameters and reevaluate the score of the other non-stationary sub-networks. These properties allow for the proposal of the following algorithm by separately learning each *HMDBN*_*i*_.

#### The SEM algorithm incorporating a heuristic searching approach

The searching space for a non-stationary DBN is much bigger than a stationary DBN. To obtain the optimal time-evolving network for each variable *X*_*i*_, we need to search among 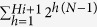 candidates. The searching space is explained as follows: given a fixed number of hidden graphs, say *h*, search for the optimal hidden graph among 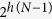 combinations of network structures, where each hidden graph has 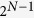 possible network structures; this step repeats with *h* changing from 1 to *H*_*i*_ + *1* at least, until the optimal *H*_*i*_is figured out, and consequently, the accurate time-evolving network is obtained. Accordingly, the total searching space is 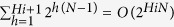.

In SEM, the BWBIC-based greedy-climbing strategy (a generalized EM algorithm) enables us to make comparisons between 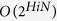 candidates and find out the accurate *HMDBN*_*i*_. However, due to huge searching space, the traditional SEM algorithm is still very time-consuming. To address this problem, we propose a heuristic approach. Its intuitive idea is similar to the greedy hill-climbing method for learning a stationary DBN: start at a random stationary DBN configuration; repeatedly change the network configuration by adding or deleting one edge until its BWBIC score cannot be improved. However, differing from learning a stationary DBN, our method, in each step, uses the Baum-Welch algorithm to infer the time-evolving probability of each single edge in the stationary DBN, and next, combine these probabilities together to infer the transition times and transform the candidate stationary DBN into the corresponding non-stationary DBN. Consequently, this heuristic approach only needs to search among 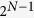
stationary networks, thereby dramatically reducing the searching space from 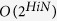 to 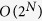 and giving a locally optimal solution.

**Figure 2:**
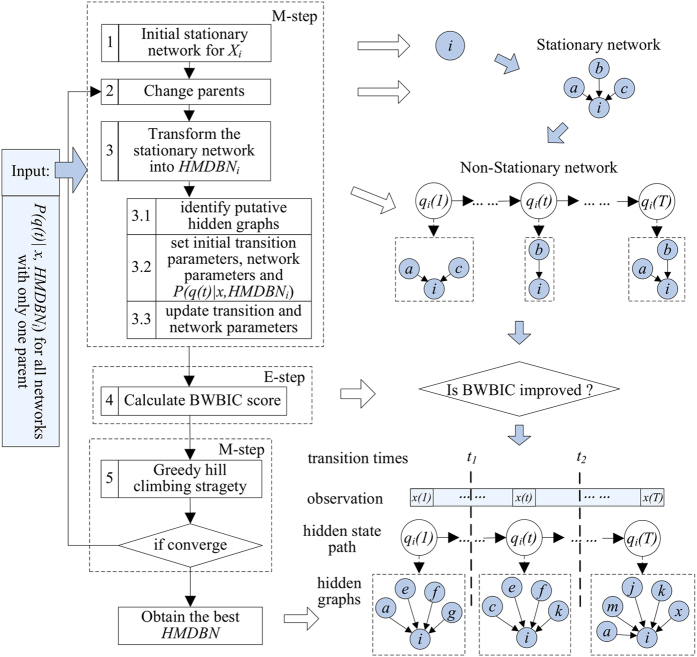
Flow chart for the HMDBN structure learning algorithm. The left are the steps in the algorithm, and the right is the output of each step.

Due to the factorization of re-estimates of parameters and scoring function as well as no contemporaneous edges, the *HMDBN* model for each variable *X*_*i*_ can be learned separately by evaluating its BWBIC score independently and adding edges without concern about feedback loops with other variables. The separate models for different variables then reconcile in the final network model. Figure 2 provides the flow chart for this algorithm. The steps are listed as follows:

1. Set a stationary network for *X*_*i*_ without parent nodes as the initial network;
2. On the basis of former stationary network, select an operation from the set {add a parent node and delete a parent node} to generate a new stationary network for *X*_*i*_;
3. Treat parent nodes in the stationary DBN as the possible parent node set of *HMDBN*_*i*_, and next, identify the accurate *HMDBN*_*i*_, which may generate the observation data most likely;
  - 3.1  identify putative hidden graphs;
  - 3.2  set initial values for 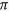, 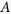 and 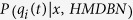, and furthermore, estimate 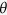 using Eq. (10);
  - 3.3  iteratively re-estimate transition parameters 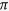 and 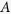 using Eq. (6, 7), refine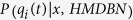, and furthermore, re-estimate parameter 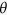 using Eq. (10);
4. Calculate the BWBIC score using Eq. (9); keep this non-stationary network, if its BWBIC score is higher than that for the former; otherwise, give it up;
5. Repeat step 2–4, until the BWBIC score cannot be improved.

Step (3) is to directly identify the number of hidden graphs, and transform the stationary network into a non-stationary network. Its detailed description is given in Supplementary File 1. Briefly, step (3) first transforms the stationary network for variable *X*_*i*_, in which *X*_*i*_ has only one parent node *X*_*j*_, into a non-stationary network *HMDBN*_*i*_. This is simple, as the corresponding non-stationary network *HMDBN*_*i*_ has at most two hidden graphs, referred to here as 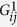 and 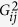, one without parent nodes and the other with one parent node *X*_*j*_. Meanwhile, distributions 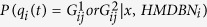 for two hidden graphs are obtained, which may reflect the extent of dependence of *X*_*i*_ on *X*_*j*_ along different time steps. Similar step applies so that we can get distributions for other parents of *X*_*i*_, e.g., 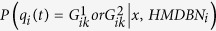. Next, the resulting distributions for *X*_*i*_ with single parent, e.g., 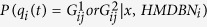 and 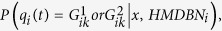 are employed to build the distribution 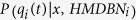
for *X*_*i*_ with two parents *X*_*j*_ and *X*_*k*_. Based on the new generated 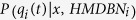, we can identify the most likely hidden graphs. Moreover, 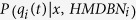 continues to be iteratively refined until it converges to the accurate value. The similar method is used to transform stationary networks with more parents.

These steps comprise the structural EM algorithm. Steps (1–3, 5) comprise the M-step, which updates the transition matrix and refines the hidden graphs, respectively, thereby improving the *Q* function. Step (4) is the E-step, which calculates the *Q* function. This algorithm does not simultaneously optimize the network structure and parameters, but instead optimizes the parameters given the fixed network structure, and next, optimizes the network structure. Either of the two steps can improve the Q function. Note that, to obtain a consensus structure prediction, existing methods separately evaluate the importance for each edge by applying a threshold for the marginal posterior probability, while our method directly identifies the network with the highest joint probability at each time point, thereby avoiding selection of thresholds.

## Results

### Evaluation Using Simulated Data

**Figure 3:**
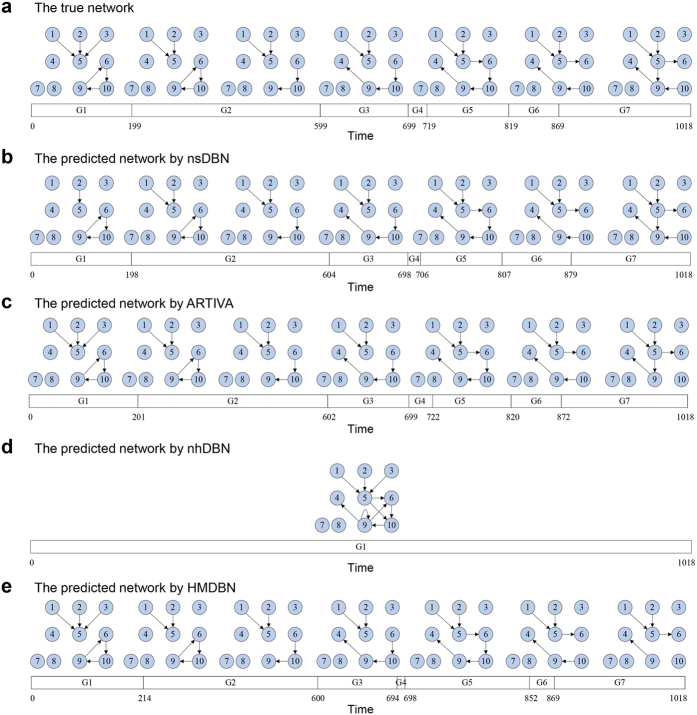
Structure learning for a simulated dataset: **(a)** The true non-stationary DBN. **(b)** The non-stationary DBN reconstructed by *nsDBN* under the settings of known transition number and unknown transition times. **(c)** The non-stationary DBN reconstructed by *ARTIVA*. **(d)** The globally fixed network reconstructed by *nhDBN* that includes the edges across all time segments. **(e)** The non-stationary DBN reconstructed by *HMDBN* without knowing transition times and the transition number.

To evaluate the effectiveness of our method, we applied it to a simulated dataset generated by the work^8^. The dataset includes 1,019 observations from an *in silico* time-varying gene regulatory network with ten nodes. The truth of the time-varying network topology is shown in Fig. 3a. There are six single-edge changes between seven epochs where the length of each epoch varies between 20 and 400 observations. To make comparisons, three representative non-stationary DBN methods, *nsDBN*^8^, *ARTIVA*^13^ and *nhDBN*^11^ were also used to recover the network topologies and transition times. The methods *nsDBN* and *ARTIVA* are RJMCMC-based discrete and continuous DBN methods, respectively, and both assume that the global network topology evolves over time; *nhDBN* is a RJMCMC-based continuous DBN method, but assumes the network topology is kept fixed, while only the parameters are allowed to vary over time.

#### Reduced running time

All methods were tested without parallel computation on a 3.2 GHz Intel Core i5 machine with 4 GB of RAM. The sampling iteration steps for *nsDBN*, *ARTIVA* and *nhDBN* are set according to their original papers^9^,^13^ or default settings of the software^11^. Table 1 gives the running time for four methods on the simulated dataset. Our *HMDBN* method takes 9 minutes to converge to the accurate result, while the others all take tens of hours. Compared to sampling methods, our method takes strikingly less time. This supports that our proposed heuristic approach largely reduces the searching space, thereby effectively decreasing the running time.

#### Outperforming prediction without parameter setting

In contrast to *HMDBN*, which learns all parameters from observation datasets, *nsDBN*, *ARTIVA* and *nhDBN* require parameter settings (part of required parameters are listed in Supplementary Table 1). Therefore, we first tested different combinations of parameters (Supplementary File 2–3) and showed their best inference results (Fig. 3b–e). Overall, the prediction by four methods are all close to the true topology. However, the best prediction by *nsDBN* is obtained with *known* transition number, while, without the prior knowledge, *nsDBN* recovers only six time segments (refer to the work^8^). The method *nhDBN* recovers all true edges, but also predicted some indirect associations; on the other hand, *nhDBN* failed to recover any transition times, even after trying different parameter settings and more sampling iterations. Among four methods, *ARTIVA* and our method *HMDBN* obtained the best reconstruction accuraries. The predicted networks by *ARTIVA* and *HMDBN* are both very close to the true topology. Moreover, both of these two methods successfully recovered seven time segments and the corresponding transition times are very close to the truth. Notably, the above results by *nsDBN*, *ARTIVA* and *nhDBN* are all obtained based on their best parameter settings. These facts suggest that our *HMDBN* method, even without parameter settings, can also obtain excellent or even better prediction.

**Figure 4:**
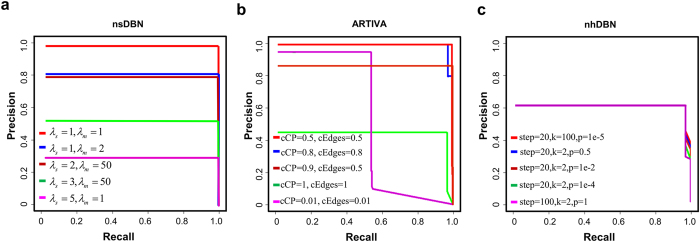
Precision and Recall curves on the simulated dataset for three methods: **(a)** *nsDBN*, **(b)** *ARTIVA*, and **(c)** *nhDBN*.The Precision does not necessarily change linearly with the varying levels of Recall. Therefore, according to the relationship between Precision-Recall and ROC curves, we first plotted the ROC curves for different thresholds, and next, translate the curves in ROC space to Precision-Recall space.

After observing the prediction under the best parameter settings by *nsDBN*, *ARTIVA* and *nhDBN*, we further explored the influence of different parameter settings on their inference results. As shown in Supplementary Files 2–3, the predicted networks by three methods are very different across different parameter settings. Since the true structures are known, we can obtain the corresponding precision and recall values. Similar to the work^8^, we calculated individual precision and recall estimates for each network at each observation, then averaged them over all observations. The precision-recall curves^25^ are drawn for each method. As shown in Fig. 4a,b, the AUC scores show a large range across the different parameter settings for both methods. The curves for *nsDBN* under (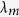 = *5*, 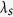 = *1*) and (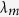 = *1*, 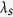 = *1*) give the highest and lowest AUC scores, and their top right points reach the corresponding highest F1-measures 0.991 and 0.434, respectively (the curves for *nsDBN* directly refer to the original paper^9^). The curves for *ARTIVA* under (*cCP* = *0.5, cEdges* = *0.5*) and (*cCP* = *0.01, cEdges* = *0.01*) give the highest and lowest AUC scores, and their top right points reach the corresponding F1-measures 0.985 and 0.578, respectively. Compared to the above two methods, the reconstructions by *nhDBN* are relatively stable under different parameter settings (Fig. 4c); all of their top right points reach the F1-measures 0.7788. In those methods, the parameter settings show the huge influence on the prediction, but the best one cannot be directly inferred from data. Thus, our method presents a distinct advantage.

**Figure 5:**
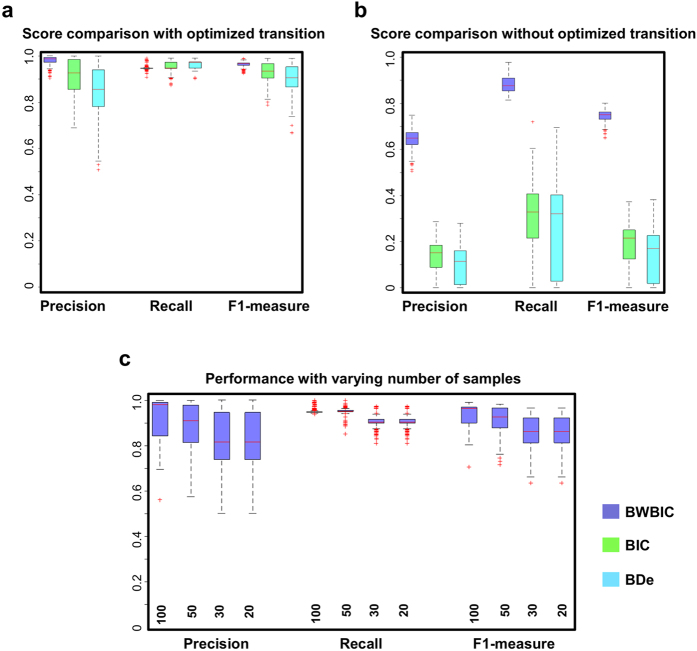
Comparsion between three metrics BWBIC, BIC and BDe. Boxplots of Precision, Recall and F1-measure values of three metrics with optimized transition probabilities **(a)** and without optimized transition probabilities **(b)**. **(c)** Prediction accuracies of *HMDBN* scales with varying number of samples. Boxplots for Precision, Recall and F1-measure values were given for 400 simulated datasets with average sample size 100, 50, 30 and 20 for each time segment.

The prediction by our method for only one simulation dataset has been shown in Fig. 3e. Next, to better demonstrate the effectiveness, we applied our method to 400 randomly generated datasets. Since our method directly identifies the network with the highest joint posterior probability at each time point, it obtained one unique precision and recall point for each dataset. The purple boxplots in Fig. 5a give the distributions of 400 precision, recall and F1-measure values by our *HMDBN* method on 400 datasets. Our method obtained stably high prediction accuracies for all datasets (for precision, median = 0.986, for recall, median = 0.945, for F1-measure, median = 0.966). Furthermore, to give an indication of our method performance with varying number of samples, we progressively reduced the sample sizes of 400 simulated datasets, with average 100, 50, 30, and 20 samples for each time segment. As shown in Fig. 5c, the prediction accuracy is gradually decreased with the reduced sample size. While the low number of samples impairs the inference of robust networks, the *HMDBN* method can still give high prediction accuracy using as few as 20 samples for each time segment (median = 0.861 for F1-measure). Additionally, to evaluate how our approach scales with a higher number of nodes, we, similarly to the work^9^, also applied our method to a 100 variable network with 50 edges over five time segments spanning 5000 samples, and one to three edges changing between each time segment. 10 datasets were simulated from this network. As a result, the medians of precision, recall and F1-measure of prediction are 0.827, 1.000 and 0.905, respectively. The reconstruction result on one of the simulated datasets is shown in Supplementary Fig. 2. Only the nodes are demonstrated, whose parent nodes evolve over time. It is shown that all of the predicted transition times are correct.

#### Reasonable metric BWBIC for non-stationary DBN

In this section, we will show that SEM-derived BWBIC score enables our *HMDBN* method to obtain stably high prediction accuracy.

Our method takes into account the information sharing for the networks across different time segments from two aspects. On the one hand, we leverage the transition between different networks to model the dependencies or similarities between networks. This tactic is similar to the information coupling proposed by the previous works, but it is not limited to the models with global information coupling^16^,^17^ or the models with sequential information coupling^8^,^9^,^14^,^15^. On the other hand, we use our derived BWBIC score to evaluate a candidate non-stationary network. Differing from the traditional BDe or BIC score, which assume that the samples in one time segment belong entirely to the corresponding network, BWBIC score assumes that one sample belongs to each network in different time segments with a certain probability. Therefore, the information from all samples can be leveraged to evaluate a network in one specific segment, even if some samples are not in this segment. This is consistent with the advantage of Baum-Welch algorithm (using weighted samples) over Viterbi training algorithm (using clearly demarcated samples) for learning a HMM^26^.

We want to investigate whether the information sharing introduced by BWBIC can help improve the prediction accuracy and reduce over-fitting, compared to traditional metrics BIC and BDe. To reduce the evaluation bias resulting from different methods, we compared with three metrics based only on our searching method. The rationale is two-fold: on the one hand, the other algorithms require additional modifications to support BWBIC; on the other hand, our method is not limited to the hypothesis of sequential or global information sharing. Moreover, to demonstrate that our searching method is unbiased for three metrics, based on our searching method, metrics BIC and BDe are also applied to the above 400 simulation datasets to reconstruct the non-stationary networks. As shown in Fig. 5a, the prediction accuracies by two metrics are also high, and very close to the accuracy by BWBIC (the median of F1-measures for BWBIC, BIC and BDe are 0.97, 0.93 and 0.91, respectively). This suggests that our proposed searching method is a general method, which can work well together with all three metrics.

Next, we explore whether these three metrics can still recover the true network without information sharing introduced by our searching method (i.e. the optimized transition probabilities). We abolish the automatic optimization and manually set the transitions between different networks equally, so that the dependencies between similar networks are removed. As shown in Fig. 5b, the F1-measure for BWBIC remains high (median = 0.75), while the F1-measures for BIC and BDe dramatically decrease to 0.21 and 0.17, respectively. The prediction results are shown in Supplementary Fig. 3. This fact suggests that, in spite of no dependencies between different networks, BWBIC can still benefit from information sharing, providing a more stable prediction than the other two metrics. Similar to our analysis, *nsDBN* has applied the sequential information sharing scheme and the extended BDe metric (nsBDe) to reconstruct the time-varying network structures. As shown in Fig. 4a, with the change of priors 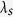 and 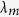, which specify the extent of information sharing, the reconstruction accuracy also deteriorates. This is consistent with our above observation, supporting the idea that traditional evaluation scores cannot benefit from information sharing to reconstruct the accurate non-stationary network.

**Figure 6:**
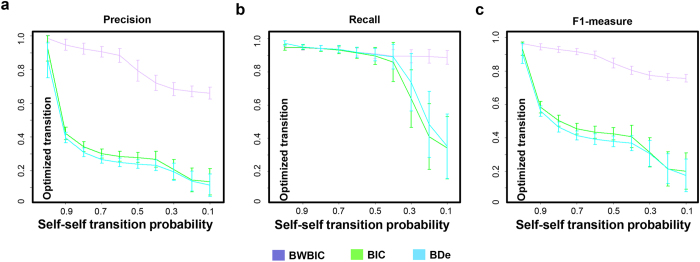
Prediction accuracies of three metrics BWBIC, BIC and BDe on simulation datasets under different parameter settings. Error bar indicates the corresponding standard deviation. **(a)** Precision, **(b)** Recall, and **(c)** F1-measure values of three metrics under different self-self transition probabilities.

Furthermore, we explored the influence of short time segments on the reconstruction accuracies of these three metrics. We progressively decreased the transition probability from one hidden network to itself and check the prediction accuracy under each setting. With the decreased self-self transition probabilities, the networks tend to transit to the others, and consequently, the time segment will be divided into small pieces. We expected that our BWBIC, by benefiting from the adjacent time segments, can still recover the accurate non-stationary network globally, even if each network has to be learned from the short time segments. As shown in Fig. 6a–c, with the decreased self-self transition probabilities, the prediction results by BIC and BDe varied wildly in precision, recall and F1-measure, coupled with the dramatic over-fitting in each short time segments. Conversely, the reconstruction accuracy by BWBIC remained stable. Even when the transition probability is decreased to 0.1, the F1-measure by BWBIC remains near 0.76, which is much better than those offered by BIC and BDe (0.20 and 0.17). This fact suggests that BWBIC can help reduce over-fitting by assuming samples belong to all networks with certain probabilities. Thus, more information sharing between networks can be included to more accurately learn the networks.

#### Evaluation Using Small-Scale *Drosophila* Gene Expression Data

To evaluate the performance of the proposed approach on real biological data, we implemented our method on *Drosophila* gene expression data^27^, which is the most frequently used real dataset for testing non-stationary network methods. This dataset contains expression measurements over 66 time points of 4028 *Drosophila* genes throughout development and growth during the embryonic, larval, pupal, and adult stages of life. The true transition times of four drosophila life periods are located at 30, 40 and 58. We preprocessed continuous expression data into binary values using the methods described by the paper^28^.

**Figure 7:**
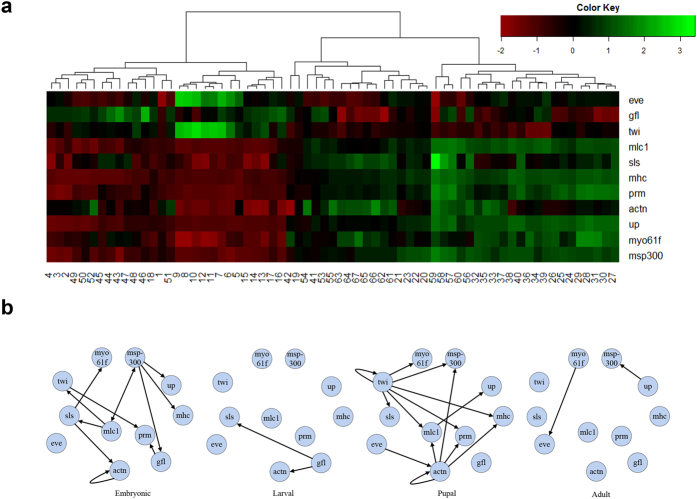
The accurate time-varying networks cannot be learned from each time segment separately. **(a)** The hierarchical clustering for the expression profiles of 11 genes across 66 time points. **(b)** The time-varying networks learned separately in each time segment.

A small set of 11 genes (*eve*, *gfl*/*lmd*, *twi*, *mlc1*, *sls*, *mhc*, *prm*, *actn*, *up*, *myo61f*, and *msp300*) was chosen for an initial analysis based on their reported involvement in *Drosophila* muscle development. Before applying non-stationary DBN methods, we first tested an intuitive approach that uses hierarchical clustering to group developmental stages, and applies a stationary DBN method to each group, respectively. This approach, however, proved to be infeasible. On the one hand, the hierarchical clustering can group expression profiles, but not regulatory relationships. As shown in Fig. 7a, the time points are mixed together by hierarchical clustering, as different expression profiles could share the same regulatory relationship. On the other hand, in spite of a big sample size across all development stages, the samples allocated to each group could be too small to recover the statistically confident relationships. As shown in Fig. 7b, four stationary DBNs are independently learned using the samples of 1:30, 31:40, 41:58 and 59:66, respectively. The networks for larval and adult, recovered using 10 and 9 samples respectively, both find only two edges. Moreover, the networks in four stages are very different from each other, which is indicative of over-fitting for each dataset in four segments. These facts to some extent support the importance of the non-stationary network method.

The methods *nsDBN, ARTIVA*, *nhDBN* and *HMDBN* were applied to this dataset. The iteration steps for the sampling-based method *nsDBN, ARTIVA*, and *nhDBN* are set according to their original papers^9^,^13^ and default settings of the software^11^. The *nsDBN, ARTIVA*, and *nhDBN* methods take 31, 25, and 49 minutes, respectively, while our method takes 2 minutes to converge to the optimal result. Next, we made the comparison between different methods from two aspects: transition time and gene regulatory network structure.

#### Transition time

**Figure 8:**
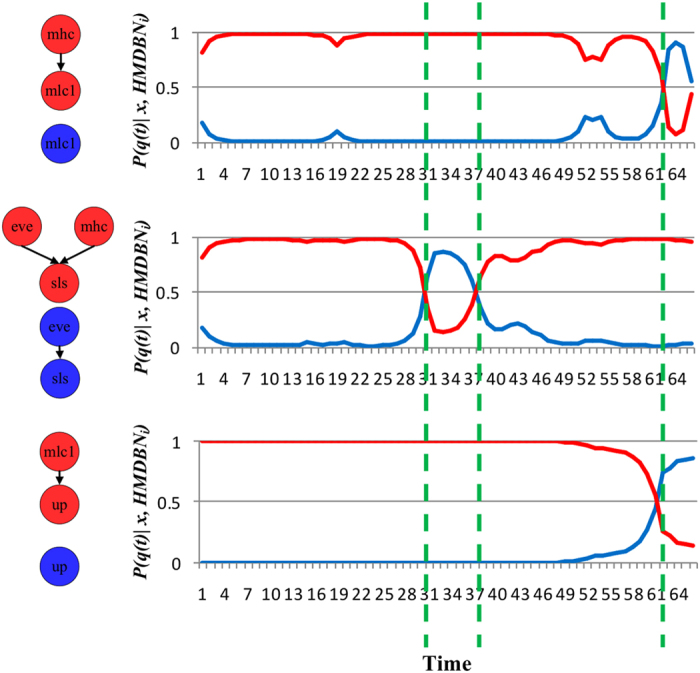
*P(q(t)|x,HMDBN)* for genes whose regulators change over time: Horizontal axis denotes time. Vertical axis represents the probability density. The red and blue curves respectively denote *P(q(t)|x,HMDBN)* over two graphs in same colors on the left of vertical axis. The green dash lines are the transition times.

**Figure 9:**
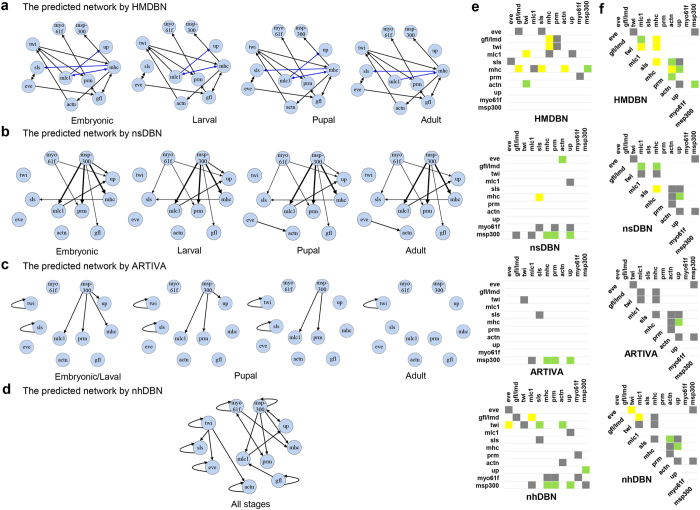
Structure learning for *Drosophila* muscle development data: **(a)** The non-stationary DBN reconstructed using our proposed method. The edges in blue are the time-varing edges across time segments. **(b)** The non-stationary DBN by *nsDBN* under the settings of known epoch number and known transition times. **(c)** The non-stationary DBN by *ARTIVA*. **(d)** The globally fixed network for all stages reported by *nhDBN*. **(e)** The retrieved interactions by four methods that are also validated by the recorded interactions. **(f)** The recorded interactions in the database that are also retrieved by four methods. The interactions marked by yellow represent the ones validated (or recovered) by direct interactions, and the interactions marked by green represent the ones validated (or recovered) by the indirect interactions via only one intermediate gene.

Presented in Fig. 8 is the predicted probability *P(q(t)|x,HMDBN)* by the *HMDBN* method for genes *mlc1*, *sls*, and *up*, whose regulators change over time. The distribution suggested that the regulators of gene *mlc1* and *up* change at time point 61, while the regulators of gene *sls* change at 31 and 37. We therefore predicted four stages whose transition times are 31, 37 and 61, which are very close to the true transition times. Furthermore, in each stage, we combined sub-networks for all genes and obtained the non-stationary DBN for the entire gene regulatory network (Fig. 9a).

Next, we tried various parameter settings for the other three methods. As shown in Supplementary File 2–3, inconsistent results were observed under different settings. Here, we present their best results, whose transition times are the closest to the truth. First, the *nsDBN* method only predicts three segments with the posterior peaks located at 11 and 21, under the best parameter setting 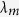 = 2, 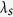 = 2, unknown transition number and unknown transition times (the best parameter settings are referred to the original paper^8^). Second, the *ARTIVA* method predicts the transition number ranging from 4 to 9, under different parameter settings. Since we know the true transition times, we report the best result, which presents four segments with the posterior peaks located at 40, 48 and 54, under the settings *cCP* = *0.5* and *cEdges* = *0.5*. To exclude the possibility that the inaccurate prediction is the result of data discretization, we also applied *ARTIVA* to the continuous dataset and tried different parameters. However, *ARTIVA* predicts only 1 to 2 time segments; moreover, under the best settings for discrete datasets, it did not predict any transition times. All predictions are shown in Supplementary File 2. Third, in contrast to the simulated datasets, the method *nhDBN* successfully predicts the transition times for the real datasets. However, the results show obvious inference uncertainty and reliance on parameter settings. With the increased change-point parameter, the predicted number of segments increases substantially, ranging from 3 to 66. The number 66 is the same with the number of time steps, which is indicative of over-fitting. The result closest to the truth is three time segments, whose transition times are located at 39 and 52 (under parameter setting *p* = *1e-4, k* = *2*). We also applied *nhDBN* to the continuous dataset and tried different parameters. Similar to the discrete datasets, the predicted transition times for continuous datasets also show obvious inference uncertainty, ranging from 3 to 66 (Supplementary File 3). These facts suggest that our method can obtain more stable and accurate prediction than the other methods, even when their best parameter settings are selected.

#### Network structure

Our *HMDBN* method predicted the unique non-stationary network structure (Fig. 9a). Similar to the transition times predicted by the other three methods, the predicted network structures are also different under various parameter settings (Supplementary File 2–3). The reference regulatory network on the muscle development of *Drosophila* is not fully available. Here, we show the best results from these three methods: the result for *nsDBN* is shown in Fig. 9b, which is obtained under the setting of *known* transition number and *known* transition times; the evolving networks for *ARTIVA* and *nhDBN* are shown in Fig. 9c,d, whose corresponding transition times are the closest to the truth among their predictions.

Despite certain similarities, we observed many differences between our results and other predictions. In the other predictions, a cluster forms around *myo61f*, *msp-300*, *up*, *mhc*, *prm*, and *mlc1*. With the exception of *up*, all of these genes are in the myosin family, which contains genes involved in muscle contraction. Our prediction also indicates intense associations among these genes, although the inferred relationships are different from those in other predictions. In our prediction, the gene *up* is not as intensely connected with this cluster as other genes, and *up* is disassociated with this cluster in the adult stage. This is consistent with the fact that gene *up* is not in the myosin family. Additionally, our results suggested that *mhc* may play a crucial role in the regulatory network for *Drosophila* muscle development, whereas other methods predicted that *msp-300* is the primary hub gene for regulating the cluster of 11 genes.

To validate the recovered relationships by different methods, we refer to the FLIGHT database^29^ and the other existing literatures, where a number of relevant biological experiments were found. The interaction network recorded by FLIGHT is shown in Supplementary Fig. 4. Based on the recorded interactions, we compared the prediction performances of different methods.

First, we assess the proportion of retrieved interactions that are validated by existing biological experiments (similar to precision). As shown in Fig. 9e, among 17 recovered interactions by our method, 8 interactions were validated, where 6 are validated by the recorded direct interactions (yellow) and 2 are validated by the recorded indirect interactions via only one intermediate gene (green). Second, we evaluated the proportion of the recorded interactions that are successfully retrieved (similar to recall). As shown in Fig. 9f, among 14 interactions in literatures, 10 interactions are recovered, where 5 are recovered directly (yellow) and 5 are recovered indirectly via only one intermediate gene (green). In contrast, the other three methods all give much lower values for both evaluations. The exact precision and recall values for all four methods are shown in Table 2.

Moreover, it is worth noting that most of the recorded interactions in FLIGHT result from undirected interactions, while one prominent evidence for directed regulatory relationship is that the gene *gfl/lmd* and *twi* are indicated to direct upstream regulators of *mef2*^30^,^31^ that directly regulates some target *myosin* family genes at all stages of muscle development^32^, such as *mhc* and *mlc1*. The method *nhDBN* and *ARTIVA* fail to obtain these relationships. Under the settings of known transition time and known transition number, the *nsDBN* still misses such associations. Our algorithm successfully captures these interactions. In our resulting network, gene *gfl* and *twi* are both direct regulators of gene *mhc*. Gene *twi* is inferred to be regulated by gene *mlc1*, and gene *mhc* and *mlc1* can regulate each other. These connections comprise a feedback loop, suggesting that there are intense regulatory effects among these genes.

In addition to the non-stationary directed graph methods, another promising time varying undirected graph method, *htERGMs*^3^, was also applied to this small gene expression dataset. Compared to our method, it also gives lower prediction accuracies in both transition times and network structures (Supplementary Fig. 5).

### Evaluation Using Large-Scale Drosophila Gene Expression Data

**Figure 10:**
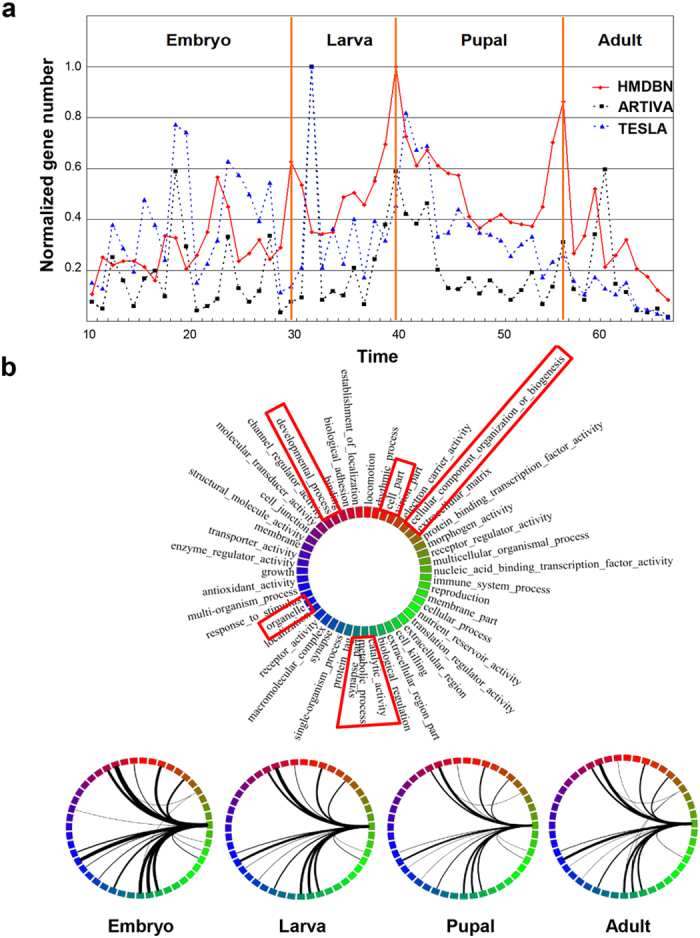
The time-evolving regulatory network for the whole set of *Drosophila* genes. **(a)** The distribution for the predicted transition times over time-points and developmental stages. The number of transition times is normalized via dividing each value by the maximum one. **(b)** The time-evolving networks among gene ontology groups during *Drosophila* development. The 4,028 measured genes are grouped according to 47 ontology groups; the weight of an edge between 2 ontology groups counts the number of connections between genes across these 2 groups. The width of an edge in the visualization is proportional to its weight. The legend for ontology groups is shown on the left of four networks. The ontologies in rectangles are the ontologies, for which the time-evolving target genes are enriched.

After evaluating our method on a small-scale dataset, we next applied our method to the whole expression dataset of 4,028 *Drosophila* genes that has been previously used to validate the methods *TESLA*^4^ and *ARTIVA*. First, similar to *ARTIVA*, we selected the potential parent genes with known transcriptional activity based on Gene Ontology information: Transcription activator activity (GO:0016563), Transcription repressor activity (GO:0016564), Transcription factor activity (GO:0003700) and Transcription cofactor activity (GO:0003712). Next, we reconstructed the network using our *HMDBN* method. Our method predicts 5,708 total edges (Embryo: 5,702, Larva: 5,354, Pupal: 5,218, Adult: 5,319 and edges shared by four stages: 4,555). Out of the 4,028 analyzed genes, 3,992 (99%) were indicated to be involved in the time-varying regulatory networks during the *Drosophila* life-cycle, suggesting that nearly all genes are regulated by transcription factors (including transcription factor themselves), whereas *ARTIVA* only inferred that 1,623 genes (40%) are regulated by 134 transcription factors (137 in total). Figure 10a shows the distribution for the predicted transition times by the three methods, *HMDBN*, *ARTIVA*, and *TESLA*. Significant peaks observed by our method are located at time points 23, 30, 40 and 56. It is known that the time points 30, 40 and 58 correspond to the transition times from embryo to larva, from larva to pupal and from pupal to adult, respectively. Furthermore, mid-embryogenesis (around 20) corresponds to a major morphological change related to a modification of transcriptional regulations^27^. Therefore, compared to the other methods, our result is even closer to the truth.

To assess our prediction, we first refer to the high-quality, manually curated database REDfly^33^, which contains a rich collection of *Drosophila* regulatory relationships. All Transcriptional Factor Binding Sites (TFBS) information given in database REDfly was employed. The REDfly TF-target network has 590 edges, connecting 169 TFs with 213 targets. The enrichment between two networks was defined as the number of interactions that are present in both networks divided by the number of such interactions expected by chance and statistical significance for the enrichment was evaluated using Fisher exact test. Our predicted network showed significant enrichment with the REDfly TF-target network, the edges being 7.86-fold more likely to be present in the REDfly network than expected by chance (p-value = 3.38e–04). Moreover, the physically interacting genes should tend to be co-regulated^34^. Therefore, we also evaluated the enrichment of Protein-Protein Interactions (PPI) for genes that are co-regulated. A set of high confidence PPIs^35^ was utilized, where high-throughput yeast two-hybrid data were excluded. By an analogous enrichment analysis to that performed on the REDfly network, our predicted network also showed a significant enrichment for PPIs (fold-enrichment = 2.56, p-value = 9.01e–03). Next, we compare our predicted network with the PPIs from all other major databases collected by the work^35^. The result also showed a strong enrichment (fold-enrichment = 4.17, p-value = 4.13e–06). Thus, all of these validations support the functional relevance of our inferred networks.

During the developmental stages of *Drosophila*, many genes are expected to play different roles at different times, with various proteins of different functions interacting. According to Flybase (http://flybase.bio.indiana.edu), 4,028 genes in our dataset are divided into 3 primary gene ontology (GO) groups: cellular component, molecular function, and biological process. They can be further divided into 47 secondary gene ontology groups. To further assess the functional relevance of our predicted network, we mapped the time varying edges to 47 secondary gene ontology groups (1,147 time-varying edges for embryo, 799 edges for larva, 663 edges for pupae, and 764 edges for adult; Fig. 10b using software Circos^36^). We selected the parent genes in advance, and accordingly, the regulators in these networks are only related to transcriptional factor binding activity. In contrast to the GO interaction network by *TELSA*, which reflects a large topology change, our predicted network shows a relatively stable topology. Through all of the stages of developmental process, we observed that the time-evolving target genes mainly belong to the following GO groups: ‘Developmental process’, ‘Organelle’, ‘Cell part’, ‘Cellular component organization or biogenesis’ and ‘Metabolic process’. The GO ‘Developmental process’ and ‘Organelle’ show the most significant differentiation. Moreover, progressive changes of regulatory extent can be observed from the temporal patterns between these gene ontology groups, and the GO ‘Developmental process’ and ‘Organelle’ are most active during embryo stage. In addition, the GO ‘Reproduction’ and ‘Transporter activity’ were found to be unique in embryo stage. These observations are all consistent with our expectation about the development, further reinforcing our confidence about the functional relevance of our inferred networks.

## Discussion

In conclusion, our proposed *HMDBN* model together with an improved SEM algorithm addressed the major problems for the existing non-stationary DBN methods: long running time and reliance on manual parameter settings. In addition, one novel non-stationary BIC score: BWBIC was derived based on SEM algorithm. This score, when compared to other traditional scoring criteria, better facilitates the information sharing, thereby largely improving prediction accuracy and reducing over-fitting.

Differing from the traditional BDe and BIC scores, BWBIC score utilized a distributed sample to learn the non-stationary network structure. The advantage of distributed samples over non-distributed samples, to some extent, has been shown from the comparison between soft EM (standard EM)^37^ and hard EM^38^. The hard EM algorithm makes a hard or demarcated choice for the hidden variable (non-distributed), while the soft EM algorithm instead determines the probability of each possible value of hidden variable for each data point, and then uses the probabilities associated with a particular value of hidden variable to compute a weighted average over the entire set of data points (distributed). The hard EM doesn’t give the full conditional likelihood of the hidden parameters, and ends up with reducing the accuracy but saving significant computational time, while the soft EM algorithm ends up with the full conditional likelihood for hidden parameters, and use the information from all samples to better estimate the parameter and obtain more robust results. Furthermore, when applied to HMM, the soft EM and hard EM algorithms correspond to Baum-Welch algorithm^21^ and Viterbi training algorithm^22^, respectively. Briefly, the Baum-Welch algorithm is essentially the soft EM algorithm applied to a HMM and guarantees to converge to at least a local maximum. The Viterbi training algorithm segments the data and then applies the Viterbi algorithm to get the most likely state sequence in the segment; next uses that most likely state sequence to re-estimate the hidden parameters. As discussed in the work^26^, Viterbi training algorithm makes a limited use of the training data, since only observations inside the segments corresponding to a given HMM state are used to re-estimate the parameters of that state, resulting in sharper but less robust models, while Baum-Welch algorithm exhaustively uses all the available data to produce robust and optimal estimates. Finally, when used for learning time-evolving DBNs, the distributed samples also showed consistent advantages over non-distributed samples. On both synthetic and real biological datasets, our work has shown that the traditional non-distributed sample-based BDe or BIC scores might lead to over-fitting, especially when the subsequent transition times are close together, and the network structures must be inferred from short time series segments; while the distributed sample-based BWBIC score can help largely improve the prediction accuracy and reduce over-fitting, and moreover, it can still maintain relatively high performance even with no other information sharing from dependencies among different networks.

In addition to the three major challenges, another relevant one is how to generate long time series data. To overcome this, we will adopt the strategy of combining multiple short time series^39^. It has been shown that data combined from multiple short time series is as informative as a long time series. Moreover, uncertainty quantification of network structures has received significant attention on stationary DBN reconstruction^40^. However, extending these results to non-stationary DBN remains an open problem: in addition to network structures, the distribution of transition times is also needed to characterize. Therefore, in the future, we will focus our work on how to appropriately estimate the uncertainty and the statistical significance for *HMDBNs*.

An extension of *ARTIVA* was recently proposed^15^ that makes promising improvements on both prediction accuracy and parameter inference. In addition, a somewhat related paper^41^ was proposed. Methodologically similar to the existing non-stationary DBN methods, it also estimates parameters via a sampling approach particle filtering. However, it is subject to the online estimation scenario with applications e.g. in tracking. This is different from most systems biology applications, where the regulatory relationship is typically recovered off-line after completing various high-throughput experiments.

Finally, in spite of only a basic framework, *HMDBN* can be easily improved by borrowing experience from abundant existing research achievements for HMM and DBN methods. We believe that *HMDBN*, as introduced here, will serve as a foundational graph theoretic tool for non-stationary directed graphs in many problems of network science and other fields. Microarray data has been used in this paper, however, due to the rapid advances in data collection technologies and deeper understanding about biological mechanisms, the high-dimensional, and feature-rich data from complex dynamic processes will grow progressively. We expect our proposed method *HMDBN* to lead to a rich set of applications and offer deeper understanding in network science, molecular and cell biology and many other fields.

## Additional Information

**Data availability**: A Matlab implementation of the algorithm is available: http://mlg.hit.edu.cn/sjzhu/HMDBN.zip

**How to cite this article**: Zhu, S. and Wang, Y. Hidden Markov induced Dynamic Bayesian Network for recovering time evolving gene regulatory networks. *Sci. Rep.* **5**, 17841; doi: 10.1038/srep17841 (2015).

## Supplementary Material

Supplementary Information

## Figures and Tables

**Table 1:** Running time of four methods on the simulated dataset.

Method	nsDBN	ARTIVA	nhDBN	HMDBN
Time	58 hours	11 hours	23 hours	9 minutes

**Table 2:** Prediction accuracies by four methods on the *Drosophila* muscle-related gene expression dataset.

Method	Precision	Recall	F1-Measure
*nsDBN*	0.45	0.29	0.35
*ARTIVA*	0.50	0.14	0.22
*nhDBN*	0.39	0.29	0.33
***HMDBN***	0.50	0.71	0.59

Both of the directly and indirectly (via only one intermediate gene) validated interactions are treated as correct; the bi-directional edges are counted only once.
