# Supplementary material for: Hidden Markov induced Dynamic Bayesian Network for recovering time evolving gene regulatory networks
Source: Sci Rep. 2015 Dec 18;5:17841. doi: 10.1038/srep17841 (PMC4683538; doi:10.1038/srep17841)
Supplement: Supplementary Information [file srep17841-s1.pdf]

SUPPLEMENTARY MATERIALS

Hidden Markov induced Dynamic Bayesian Network for recovering time evolving gene regulatory networks

Shijia Zhu[1] and Yadong Wang[1, *]

[1]Center for Biomedical Informatics, School of Computer Science and Technology, Harbin Institute of Technology, Harbin, Heilongjiang, 150001, China

[*]Corresponding: ydwang@hit.edu.cn

Contents

Supplementary File 1. Derivation of the BWBIC score and the detailed *HMDBN* learning algorithm.

Supplementary File 2. Prediction results of *ARTIVA* on the simulated dataset and real biological dataset under different parameter settings.

Supplementary File 3. Prediction results of *nhDBN* on the simulated dataset and real biological dataset under different parameter settings.

Supplementary Table 1. Part of required parameter settings for *nsDBN*, *ARTIVA*, and *nhDBN*.

Supplementary Fig. 1. Illustration for the transformation from a stationary DBN to a *HMDBN*.

Supplementary Fig. 2. *P(q(t)|x,HMDBN)* of one dataset generated by a big network.

Supplementary Fig. 3. *P(q(t)|x,HMDBN)* of one simulated dataset recovered by three metrics BWBIC, BIC, and BDe without optimized transition probabilities.

Supplementary Fig. 4. The database FLIGHT-recorded gene interactions.

Supplementary Fig. 5. The *Drosophila* muscle-related gene interaction network predicted by the undirected non-stationary graph method *htEGRMs*.

Supplementary File 1: Derivation of the BWBIC Metric and the Detailed HMDBN Structure Learning Algorithm

Learning HMDBN Using an improved SEM algorithm

Here, we use the SEM algorithm to learn the HMDBN structure. There are three challenges.

First, the SEM algorithm, compared to the standard EM algorithm, defines an objective term involving not only parameters but also network structures. In the Maximization step (M-step), the objective term is very hard to maximize with respect to the network structure, since it is a NP-complete problem. In the Expectation step (E-step), it is still difficult to estimate with respect to a fixed network structure.

Second, the SEM algorithm works with a fixed set of hidden graphs, therefore, an additional approach is required to identify how many hidden graphs to be included in HMDBN. This can be done using a simple loop with different numbers of hidden graphs, however, it is very time-consuming.

Third, even if the number of hidden graphs is determined, we still need to search for the optimal HMDBN among a huge searching space.

To address the first problem, we derive an approximation to the SEM objective function, which is efficient to calculate in the E-step. This approximation is a novel and reasonable evaluation metric for non-stationary networks. Based on this metric, a greedy-climbing search method is used to find the optimal non-stationary network in the M-step. To address the second and the third problems, instead of trying different numbers of hidden graphs and multiple network structures for each hidden graph, we proposed a heuristic approach to directly identify the putative number of hidden graphs and the corresponding network structures. This approach greatly reduces the searching space.

Transition Matrix Re-estimation and BWBIC Metric:

Suppose the observable value, $x = \{x_i(t)\}_{1\le i\le N; 0\le t\le T}$, represents the temporal observation data; the missing value, $q = \{q_i\ (t)\}_{1\le i\le N; 0\le t\le T}$, represents the hidden graph sequence for observation data; $HMDBN = \{\pi_i\ , A_i\ , G_i\ , \theta_i\ \}_{1\le i\le N}$ represents the set of initial hidden graph distribution, hidden graph transition matrix, non-stationary sub-network structure and network parameters, which most likely generate the observation *x* and hidden graph sequence *q*.

The E-step of the SEM algorithm searches for the value of the *Q* function for *HMDBN* model, i.e. the expected value of $\log p(x, q \mid HMDBN)$ with respect to the current estimate of *HMDBN*, represented by $HMDBN'$,

$$Q(HMDBN, HMDBN') = \sum_q \log P(x, q \mid HMDBN) P(q \mid x, HMDBN') \tag{1}$$

We assume that the transition between hidden graphs follows the 1st order Markov chain, and that the current observation is statistically independent of the previous observations. Accordingly, it is easy to prove that

$$\begin{aligned} &P(x, q \mid HMDBN) = P(q \mid HMDBN) P(x \mid q, HMDBN) \\ &= P(q(1)) \prod_{t=2}^{T} P(q(t) \mid q(t-1)) \prod_{t=1}^{T} P(x(t) \mid q(t)) \end{aligned} \tag{2}$$

Additionally, we assume that hidden state q_i for variable X_i is statistically independent of those for other variables. Meanwhile, the hidden graph, which is also a DBN, follows the conditional independence. So, the above formula becomes

$$\begin{aligned} &\prod_{i=1}^{N} P(q_i\ (1)) \prod_{i=1}^{N} \prod_{t=2}^{T} P(q_i\ (t) \mid q_i\ (t-1)) \prod_{i=1}^{N} \prod_{t=1}^{T} P(x_i(t) \mid q_i\ (t)) \\ &= \prod_{i=1}^{N} \left(\pi_i^{qi(1)} \prod_{t=2}^{T} a_i^{qi(t-1)qi(t)} \prod_{t=1}^{T} P\left(x_i(t) \mid G_i^{qi(t)}, \theta_i^{qi(t)}\right) \right) \end{aligned} \tag{3}$$

By introducing the above formula into the *Q* function, we get that

$$Q(HMDBN, HMDBN') = \sum_q \log P(x, q \mid HMDBN) P(q \mid x, HMDBN')$$

$$= \sum_{i=1}^{N}\left(\sum_{q}\log \pi_i^{qi(1)} P\left(q \mid x, HMDBN'\right) + \sum_{q}\sum_{t=2}^{T}\log a_i^{qi(t-1)qi(t)} P\left(q \mid x, HMDBN'\right) + \sum_{q}\sum_{t=1}^{T}\log P\left(x_i(t) \mid G_i^{qi(t)}, \theta_i^{qi(t)}\right) P\left(q \mid x, HMDBN'\right)\right) \tag{4}$$

Then, for each non-stationary sub-network $HMDBN_i$, the Q function takes the following form:

$$Q\left(HMDBN_i, HMDBN_i'\right)$$
$$= \sum_{q}\log \pi_i^{qi(1)} P\left(q \mid x, HMDBN'\right) + \sum_{q}\sum_{t=2}^{T}\log a_i^{qi(t-1)qi(t)} P\left(q \mid x, HMDBN'\right)$$
$$+ \sum_{q}\sum_{t=1}^{T}\log P\left(x_i(t) \mid G_i^{qi(t)}, \theta_i^{qi(t)}\right) P\left(q \mid x, HMDBN'\right) \tag{5}$$

For the first term,

$$\sum_{q}\log \pi_i^{qi(1)} P\left(q \mid x, HMDBN'\right)$$
$$= \sum_{q1(1)}\cdots\sum_{q1(\mathrm{T})}\cdots\sum_{qi-1(1)}\cdots\sum_{qi-1(\mathrm{T})}\sum_{qi(1)}\cdots\sum_{qi(\mathrm{T})}\cdots\sum_{qN(1)}\cdots\sum_{qN(\mathrm{T})}$$
$$\log \pi_i^{qi(1)} P\left(q_1(1),\ldots,q_1(\mathrm{T}),\ldots q_{i-1}(1),\ldots,q_{i-1}(\mathrm{T}) q_i(1),\ldots,q_i(\mathrm{T})\ldots,q_N(1),\ldots,q_N(\mathrm{T}) \mid x, HMDBN'\right)$$
$$= \sum_{q_i(1)}\log \pi_i^{qi(1)} \sum_{q1(1)}\cdots\sum_{q1(\mathrm{T})}\cdots\sum_{qi-1(\mathrm{T})}\sum_{qi(2)}\cdots\sum_{qi(\mathrm{T})}\cdots\sum_{qN(1)}\cdots\sum_{qN(\mathrm{T})}$$
$$P\left(q_1(1),\ldots,q_1(\mathrm{T}),\ldots q_{i-1}(1),\ldots,q_{i-1}(\mathrm{T}) q_i(1),\ldots,q_i(\mathrm{T})\ldots,q_N(1),\ldots,q_N(\mathrm{T}) \mid x, HMDBN'\right)$$
$$= \sum_{qi(1)}\log \pi_i^{qi(1)} P\left(q_i(1) \mid x, HMDBN'\right)$$
$$= \sum_{h=1}^{H_i}\log \pi_i^{h} P\left(q_i(1) = h \mid x, HMDBN'\right) \tag{6}$$

By optimization, it yields

$$\pi_i^{h\prime} = P\left(x, q_i(1) = h \mid HMDBN'\right) \Big/ \sum_{u=1}^{Hi} P\left(x, q_i(1) = u \mid HMDBN'\right) \tag{7}$$

For the second term, a similar process applies

$$\sum_{q}\sum_{t=2}^{T}\log a_i^{qi(t-1)qi(t)} P\left(q \mid x, HMDBN'\right)$$
$$= \sum_{h=1}^{Hi}\sum_{u=1}^{Hi}\sum_{t=2}^{T}\log a_i^{hu} P\left(q_i(t-1) = h, q_i(t) = u \mid x, HMDBN'\right) \tag{8}$$

By optimization, it yields,

$$a_i^{hu\prime} = \sum_{t=2}^{T} P\left(x, q_i(t-1) = h, q_i(t) = u \mid HMDBN'\right) \Big/ \sum_{t=2}^{T} P\left(x, q_i(t-1) = h \mid HMDBN'\right) \tag{9}$$

For the third term,

$$\sum_{q}\sum_{t=1}^{T}\log P\left(x_i(t) \mid G_i^{qi(t)}, \theta_i^{qi(t)}\right) P\left(q \mid x, HMDBN'\right)$$
$$= \sum_{h=1}^{Hi}\sum_{t=1}^{T}\log P\left(x_i(t) \mid G_i^{h}, \theta_i^{h}\right) P\left(q_i(t) = h \mid x, HMDBN'\right) \tag{10}$$

Then, for each hidden graph G_i^h, the expected likelihood is

$$\sum_{t=1}^{T}\log P\left(x_i(t) \mid G_i^{h}, \theta_i^{h}\right) P\left(q_i(t) = h \mid x, HMDBN'\right) = \log \prod_{t=1}^{T} P\left(x_i(t) \mid G_i^{h}, \theta_i^{h}\right)^{P\left(qi(t)=h \mid x, HMDBN'\right)} \tag{11}$$

Next, to obtain $G_i^{h\prime}$, which optimizes $\prod_{t=1}^{T} P\left(x_i(t) \mid G_i^{h}, \theta_i^{h}\right)^{P\left(qi(t)=h \mid x, HMDBN'\right)}$, we first get the marginal likelihood over θ_i^h, and then, solve the following optimization problem,

$$G_i^{h\prime} = \underset{G_i^h}{\arg\max} \log \int \prod_{t=1}^{T} P\left(x_i(t) \mid G_i^{h}, \theta_i^{h}\right)^{P\left(qi(t)=h \mid x, HMDBN'\right)} P\left(\theta_i^h \mid G_i^h\right) d\theta_i^h \tag{12}$$

This term, however, is very hard to maximize directly with respect to the network structure G in the M-step, since it is a NP-complete problem. To address this problem, the SEM algorithm does not maximize the marginal likelihood at each iteration, but attempts to find a better network structure that progressively improves the marginal likelihood. This is a generalized EM algorithm, which still guarantees to converge to a local maximum. However, the marginal likelihood is also difficult to calculate with respect to a network structure. To get an efficient way, we derived an asymptotic

approximation to this integral using a large-sample approximation technique, Laplace approximation. The basic idea is that, as the sample size increases, the above integral can be approximated around its point of maximum using a multivariate Gaussian distribution.

First, for simplicity, we let $l\left(\theta_i^h\right)=\log\prod_{t=1}^{T}P\left(x_i(t)\mid G_i^h,\theta_i^h\right)^{P\left(qi(t)=h|x,HMDBN'\right)}$, and then, the above term becomes $\log\int\exp\{l\left(\theta_i^h\right)\}p\left(\theta_i^h\mid G_i^h\right)d\theta_i^h$.

Also, we define $\theta_i^{h\prime}$ to be the configuration of θ_i^h that maximizes $l\left(\theta_i^h\right)$ and makes the first derivative $l'\left(\theta_i^{h\prime}\right)=0$. It is known as Maximization Likelihood Estimation (MLE). Using a second degree Taylor expansion of $l\left(\theta_i^h\right)$ around $\theta_i^{h\prime}$, we get

$$l\left(\theta_i^h\right)\approx l\left(\theta_i^{h\prime}\right)+\frac{1}{2}\left(\theta_i^h-\theta_i^{h\prime}\right)^T l''\left(\theta_i^{h\prime}\right)\left(\theta_i^h-\theta_i^{h\prime}\right) \tag{13}$$

where $\left(\theta_i^h-\theta_i^{h\prime}\right)^T$ is the transpose of vector $\left(\theta_i^h-\theta_i^{h\prime}\right)$, and $l''\left(\theta_i^{h\prime}\right)$ is the Hessian matrix of $l\left(\theta_i^h\right)$ evaluated at $\theta_i^{h\prime}$. We use A_h to denote $-l''\left(\theta_i^{h\prime}\right)$. By assuming the smoothness of $p(\theta_i^h\mid G_i^h)$ in the neighborhood of $\theta_i^{h\prime}$, we can obtain $p(\theta_i^h\mid G_i^h)\approx p(\theta_i^{h\prime}\mid G_i^h)$ in a small neighborhood $nb\left(\theta_i^{h\prime}\right)$. Then, by introducing the Taylor expansion into the integral, we get that

$$\begin{aligned}&\int\exp\{l\left(\theta_i^h\right)\}p\left(\theta_i^h\mid G_i^h\right)d\theta_i^h\\ &\approx\exp\left\{l\left(\theta_i^{h\prime}\right)\right\}P\left(\theta_i^{h\prime}\mid G_i^h\right)\times\int_{nb(\theta_i^{h\prime})}\exp\left\{-\frac{1}{2}\left(\theta_i^h-\theta_i^{h\prime}\right)^T A_h\left(\theta_i^h-\theta_i^{h\prime}\right)\right\}d\theta_i^h\end{aligned} \tag{14}$$

Now, there remains only one integral over θ_i^h. We can approximate it using the integral for a multivariate Gaussian distribution. Variable θ_i^h is composed of d_i^h independent variables, where $d_i^h=g_i^h\left(r_i-1\right)$. $\theta_i^{h\prime}$ and A_h are treated as the mean and covariance matrix of Gaussian distribution, respectively. Then, the density function of Gaussian distribution is

$$\frac{1}{\sqrt{(2\pi)^{d_i^h}\left|A_h\right|^{-1}}}\exp\left\{-\frac{1}{2}\left(\theta_i^h-\theta_i^{h\prime}\right)^T A_h\left(\theta_i^h-\theta_i^{h\prime}\right)\right\} \tag{15}$$

So, we get that

$$\int_{nb(\theta_i^{h\prime})}\exp\left\{-\frac{1}{2}\left(\theta_i^h-\theta_i^{h\prime}\right)^T A_h\left(\theta_i^h-\theta_i^{h\prime}\right)\right\}d\theta_i^h\approx\sqrt{(2\pi)^{d_i^h}\left|A_h\right|^{-1}} \tag{16}$$

Then, we can obtain that

$$\log\int_{\theta_i^h}\exp\{l\left(\theta_i^h\right)\}P\left(\theta_i^h\mid G_i^h\right)d\theta_i^h\approx l\left(\theta_i^{h\prime}\right)-\frac{1}{2}\log\left|A_h\right|+\log P\left(\theta_i^{h\prime}\mid G_i^h\right)+\frac{d_i^h}{2}\log\left(2\pi\right) \tag{17}$$

$\left|A_h\right|$ represents the determinant of Hessian matrix A_h. $\log\left|A_h\right|$ can be approximated by $d_i^h\log\left(m_i^h\right)$, where m_i^h is the sample size. Here, we let m_i^h be the expected sample size, i.e., $m_i^h=\sum_{t=1}^{T}P\left(q_i(t)=h\mid x,HMDBN'\right)$. The last two items do not rely on the sample size m_i^h, so, we ignore them. Then, we get that

$$\log\int\exp\{l\left(\theta_i^h\right)\}p\left(\theta_i^h\mid G_i^h\right)d\theta_i^h\approx l\left(\theta_i^{h\prime}\right)-\frac{d_i^h}{2}\log m_i^h \tag{18}$$

Finally, we will show the expression of $l\left(\theta_i^{h\prime}\right)$. Suppose θ_i^h are multinomially distributed; r_i and g_i^h are the numbers of discrete states of variable X_i and its parent set G_i^h, respectively. Then, we can get that

$$l\left(\theta_i^h\right)=\sum_{j=1}^{g_i^h}\sum_{k=1}^{ri}\sum_{t=1}^{T}P\left(q_i(t)=h\mid x,HMDBN'\right)\chi\left(i,j,k:x(t)\right)\log\theta_{i,jk}^h\ , \tag{19}$$

where $\theta_{i,jk}^{h} = P\left(X_i = k \mid G_i^h = j\right)$, and $\chi\left(i,j,k:x(t)\right) = \begin{cases} 1, if \quad x_i(t) = k \quad and \quad G_i^h = j, in \quad x(t) \\ 0, otherwise \end{cases}$

According to the MLE, $l\left(\theta_i^h\right)$ reaches its maximum at

$$\theta_{i,jk}^{h}{}' = \frac{\sum_{t=1}^{T} \chi\left(i,j,k:x(t)\right) P\left(q_i(t) = h \mid x, HMDBN'\right)}{\sum_{k=1}^{ri} \sum_{t=1}^{T} \chi\left(i,j,k:x(t)\right) P\left(q_i(t) = h \mid x, HMDBN'\right)} \tag{20}$$

This approximation can be used as a scoring function to evaluate a non-stationary network. We call this novel metric *Baum-Welch Bayesian Information Criterion* (BWBIC). Its first term $l\left(\theta_i^{h'}\right)$ is the maximized value of the likelihood function of the model; the second term $\frac{d_i^h}{2}\log m_i^h$ is the penalty for the model complexity, including the number of edges and the number of hidden graphs. With the number of edges increased, more penalties are placed on the candidate non-stationary DBN; likewise, with the number of hidden graphs increased, the number of edges increases, and more penalties are also placed consequently.

Compared to the traditional metrics, BIC and BDe, the SEM-derived metric BWBIC better facilitates the information sharing across different time segments. It does not allocate one sample to a hidden graph totally, but instead with the probability $P\left(q_i(t) \mid x, HMDBN\right)$. Thus, the information from one sample could be shared by different hidden graphs at the same time step, and the information from all samples could be borrowed to evaluate each hidden graph in one candidate non-stationary network. To be specific, in BWBIC score, the information sharing or distributed sample could be used to better estimate the optimal network parameters $\theta_i^{h'}$ in the maximized likelihood term, and the expected sample size m_i^h in the penalty term. Accordingly, our metric more reasonably evaluates one candidate non-stationary network than the existing extended non-stationary scores, and to some extent helps reduce over-fitting.

The Structural EM algorithm incorporating a heuristic approach

Figure 2 shows the flow chart of our proposed algorithm for learning $HMDBN_i$. The steps are listed as follows:

1. set a stationary network for X_i without parent nodes as the initial network;

2. on the basis of former stationary network, select an operation from the set {add a parent node and delete a parent node} to generate a new stationary network for X_i;

3. treat parent nodes in the stationary DBN as the possible parent node set of $HMDBN_i$, and next, identify the accurate $HMDBN_i$, which may generate the observation data most likely;

3.1 identify putative hidden graphs;

3.2 set initial values for π, A and $P\left(q_i(t) \mid x, HMDBN\right)$, and furthermore, set initial values for θ using Eq. (20);

3.3 iteratively re-estimate transition parameters using Eq. (7 and 9), refine $P\left(q_i(t) \mid x, HMDBN\right)$, and furthermore, re-estimate parameter θ using Eq. (20);

4. calculate the BWBIC score using Eq. (18); keep this non-stationary network, if its BWBIC score is higher than that for the former; otherwise, give it up;

5. repeat step 2-4, until the BWBIC score cannot be improved.

Except the step (3), this approach is similar to the traditional greedy hill-climbing method for learning a stationary DBN. It searches for the optimal network among stationary networks instead of non-stationary networks. Differing from the stationary DBN learning method, step (3) transforms the stationary network into its corresponding non-stationary network based on the observation data, and directly identifies the number of hidden graphs. This approach searches among only $2^{(N-1)}$ stationary networks. It can greatly reduce the computation complexity and speed up the proposed method. The step (1-2, 4-5) is easy to implement, so, we will only show details of the step (3) in the following.

Starting with a simple case, a stationary sub-network for variable X_i, in which X_i has only one parent node X_j, can be easily transformed into a non-stationary network $HMDBN_i$. This is because the corresponding non-stationary network $HMDBN_i$ has at most two hidden graphs, one without parent nodes and the other with one parent node X_j. As shown in

Supplementary Fig. 1a, the graph on the left is the stationary sub-network for X_i; the graph in the middle is the corresponding $HMDBN_i$, which has two hidden graphs (the red and blue ones). Next, based on the observation dataset, step (3.2-3.3) identify the transition time between these two hidden graphs. Since we have no prior knowledge about which hidden graph generates each sample, the same initial values of the probability $P\left(q_i(t) \mid x, HMDBN\right)$ are placed on two hidden graphs for the observations at each time point. As shown in the middle of **Supplementary Fig. 1a**, two $P\left(q_i(t) \mid x, HMDBN\right)$ curves correspond to the hidden graphs with the same color, respectively. Furthermore, based on such two distributions, Eq. (7) and Eq. (9) were used to iteratively re-estimate the transition parameter π and A, and next, based on the re-estimated transition parameters, Baum-Welch algorithm was used to update $P\left(q_i(t) \mid x, HMDBN\right)$. The final optimized $P\left(q_i(t) \mid x, HMDBN\right)$ is obtained until the transition parameters cannot be improved. From $P\left(q_i(t) \mid x, HMDBN\right)$ (the right of **Supplementary Fig. 1a**), we can identify the transition times. Note that, after decoding, the probability $P\left(q_i(t) \mid x, HMDBN\right)$ on one hidden graph might be bigger than that on the other at all time points. This suggests that only one of the two putative hidden graphs really exists.

In the above simple case, the probability $P\left(q_i(t) \mid x, HMDBN\right)$ reflects the extent of dependence of X_i on X_j. As illustrated on the right of **Supplementary Fig. 1a**, before time t, the red $P\left(q_i(t) = G_{ij}^1 \mid x, HMDBN\right)$ is bigger than the blue $P\left(q_i(t) = G_{ij}^2 \mid x, HMDBN\right)$, suggesting that X_j is not the parent node of X_i before time t, while after time t, the blue $P\left(q_i(t) = G_{ij}^2 \mid x, HMDBN\right)$ is bigger than the red $P\left(q_i(t) = G_{ij}^1 \mid x, HMDBN\right)$, suggesting that X_j might be the parent node of X_i after time t. Next, motivated by these observations, we transform a stationary network for variable X_i into a *HMDBN*, where X_i has more than one parent node.

As shown in **Supplementary Fig. 1b**, an example was taken to illustrate the transformation. In the stationary sub-network (the blank graph in **Supplementary Fig. 1b**), variable X_i has two parent nodes X_j and X_k. To transform it into a *HMDBN*, we consider two $P\left(q_i(t) \mid x, HMDBN\right)$ distributions for $HMDBN_{ij}$ and $HMDBN_{ik}$, where X_j and X_k are respectively the parent nodes of X_i. There are three steps:

1) Identification of putative hidden graphs. First, we use the similar procedure in **Supplementary Fig. 1a** to obtain two distributions $P\left(q_i(t) \mid x, HMDBN\right)$ for $HMDBN_{ij}$ and $HMDBN_{ik}$, which have only one single parent node X_j and X_k, respectively. The resulting distributions are shown at the bottom of **Supplementary Fig. 1b**, marked by red and blue. Next, we aligned these two groups of distributions, combined the hidden graphs in each segment, and identify the putative hidden graphs with multiple parent nodes. As shown in **Supplementary Fig. 1b**, before time t_1, the red ones are bigger than the blue ones in both $P\left(q_i(t) \mid x, HMDBN_{ij}\right)$ and $P\left(q_i(t) \mid x, HMDBN_{ik}\right)$, suggesting that both X_j and X_k are not the parents of X_i before timestep t_1, and accordingly, in the resulting hidden graph G_i^1 (the green graph on the top of **Supplementary Fig. 1b**), there is only one node X_i and no edges. Next, between time t_1 and t_2, the blue one in $P\left(q_i(t) \mid x, HMDBN_{ij}\right)$ is bigger than the red one, suggesting that X_j is the parent of X_i, while the red one in $P\left(q_i(t) \mid x, HMDBN_{ik}\right)$ is bigger than the blue one, suggesting X_k is not the parent of X_i, and therefore, in the resulting hidden graph G_i^2 between t_1 and t_2 (the dark green graph on the top of **Supplementary Fig. 1b**), there are two nodes (X_i and X_j) and one edge ($X_j \rightarrow X_i$). After time t_2, the blue ones are always bigger than the red ones in both $P\left(q_i(t) \mid x, HMDBN_{ij}\right)$ and $P\left(q_i(t) \mid x, HMDBN_{ik}\right)$, suggesting that both X_j and X_k are the parents of X_i after time t_2, and accordingly, in the resulting hidden graph G_i^3 (the purple graph on the top of **Supplementary Fig. 1b**), there are three nodes (X_i, X_j and X_k) and two edge ($X_j \rightarrow X_i$ and $X_k \rightarrow X_i$). Thus, three hidden graphs G_i^1, G_i^2 and G_i^3 were obtained, which were constructed by (G_{ij}^1, G_{ik}^1), (G_{ij}^2, G_{ik}^1) and (G_{ij}^2, G_{ik}^2), respectively. Moreover, two transition times t_1 and t_2 were determined (dash lines in **Supplementary Fig. 1b**). Notably, X_k alone as parent is not considered, because $P\left(q_i(t) \mid x, HMDBN_{ij}\right)$ and $P\left(q_i(t) \mid x, HMDBN_{ik}\right)$ do not overlap, when the red one is bigger than the blue one in $P\left(q_i(t) \mid x, HMDBN_{ij}\right)$ and the blue one is bigger than the red one in $P\left(q_i(t) \mid x, HMDBN_{ik}\right)$.

2) Set initial values for $P\left(q(t) \mid x, HMDBN\right)$, transition parameters and network parameters. The initial values of transition parameters are very important for the convergence rate of re-estimation. Here, we set the transition probability

from one hidden graph to another as *1/m*, and the probability from each hidden graph to itself as *1-h/m*, where *m* is the size of samples and *h* is the number of time segments. Additionally, we used the $P\left(q(t)\,|\,x, HMDBN\right)$ of *HMDBN* with one parent to build the initial $P\left(q_i(t)\,|\,x, HMDBN\right)$ of *HMDBN* with multiple parents. In the above example, G_i^3 is constructed from G_{ij}^2 of *HMDBN*$_{ij}$ and G_{ik}^2 of *HMDBN*$_{ik}$, so, to build its initial values, we multiplied $P\left(q_i(t)=G_{ij}^2\,|\,x, HMDBN_{ij}\right)$ and $P\left(q_i(t)=G_{jk}^2\,|\,x, HMDBN_{ij}\right)$ for each sample (two blue curves in **Supplementary Fig. 1b**), and treated it as the initial value of $P\left(q_i(t)=G_i^3\,|\,x, HMDBN_i\right)$ (purple curve in **Supplementary Fig. 1b**). Likewise, we respectively obtained $P\left(q_i(t)=G_i^1\,|\,x, HMDBN_i\right)$ and $P\left(q_i(t)=G_i^2\,|\,x, HMDBN_i\right)$. Furthermore, we normalize them by dividing each of them by their sum. Next, based on the resulting $P\left(q_i(t)\,|\,x, HMDBN_i\right)$, we use Eq. (20) to estimate the values of network parameter θ;

3) Further refinement of $P\left(q(t)\,|\,x, HMDBN\right)$. It is worth noting that the obtained hidden graphs and transition times are not the final ones. The initial values of $P\left(q_i(t)\,|\,x, HMDBN_i\right)$ for hidden graphs G_i^1, G_i^2 and G_i^3 have been obtained above; furthermore, the transition parameters are re-estimated using the Eq. (7 and 9), and next, based on the updated transition parameters, $P\left(q_i(t)\,|\,x, HMDBN_i\right)$ are recalculated. Meanwhile, the transition times are further refined. After decoding, the probability $P\left(q_i(t)\,|\,x, HMDBN_i\right)$ for a specific hidden graph might be also always smaller than other hidden graphs. This suggests that this putative hidden graph does not really exist.

Likewise, the above procedure is used to transform a stationary network with more parent nodes into a *HMDBN*. The probabilities $P\left(q_i(t)\,|\,x, HMDBN_i\right)$ for networks with one parent are used repeatedly. Therefore, we calculate the probability $P\left(q_i(t)\,|\,x, HMDBN_i\right)$ for all networks with only one parent in advance.

Compared to the existing time-varying DBN methods, our method infers all parameters from observation datasets, including the transition number, transition matrix and hidden graph structures as well. Additionally, our method does not use a sampling method but instead a search method to recover the non-stationary DBN; on this basis, it incorporates a heuristic strategy to further reduce the searching space, therefore, it can fast converge to a local optimal solution.

Finally, note that our approach reduces the searching space for non-stationary DBN to the identical one with a stationary DBN. However, different from learning a stationary DBN, which only optimizes network structures, learning a non-stationary DBN needs to estimate the transition times as well. Our method iteratively re-estimates transition probabilities, and further, uses Viterbi algorithm to update transition times. This resembles the RJMCMC-based non-stationary DBN methods, which iteratively sample the local shift for transition times. Thus, our method reduces the searching space, although learning an accurate non-stationary DBN by our method still cannot be as time-efficient as a stationary DBN.

Supplementary Table 1 Required parameter settings for *nsDBN*, *ARTIVA*, and *nhDBN*.

Method	Hyper Priors and Parameters
nsDBN	*N* and *T*: Known (*K*) or Unknown (*U*) transition number (*N*) and transition times (*T*);<br>λ_m and λ_s : the penalty coefficients for the number of edges and transitions;<br>More parameters refer to the work [1, 2] .
ARTIVA	α_{CP} , β_{CP} , α_{Edges} *and* β_{Edges} : parameters for sampling the transition number and edges;<br>*cCP* and *cEdges*: maximal probabilities to propose the change-point birth (resp. death) and the edge move (birth or death of an edge);<br>More parameters refer to the work [3] .
nhDBN	*p* and *k*: parameters of the negative binomial distribution underlying the point process prior for change-points.

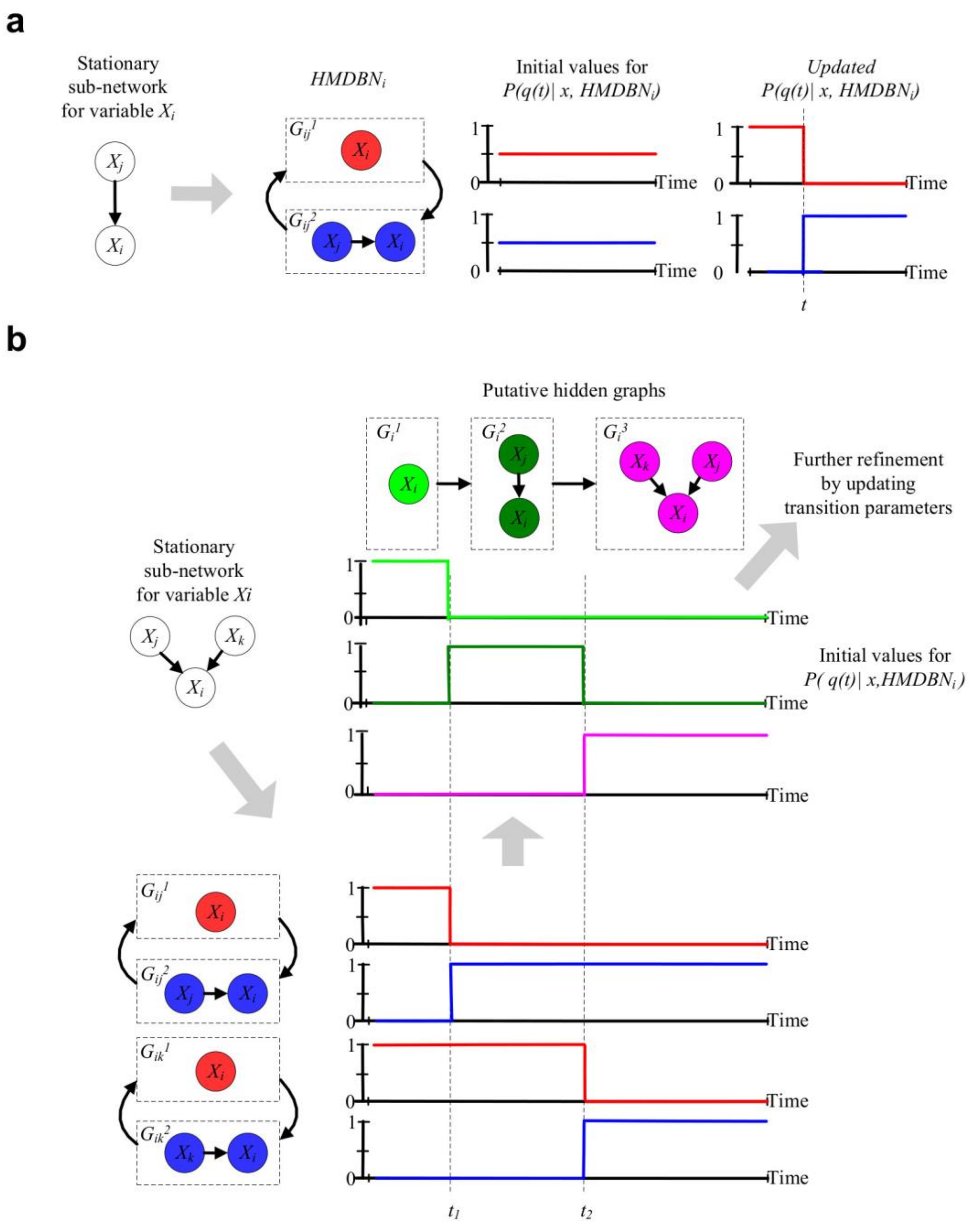

Supplementary Fig. 1. Transformation from a stationary DBN to a *HMDBN*: the horizontal axis represents the temporal observation sequence; the vertical axis represents the probability density. The curves on the right of vertical axis represent the probabilities $P(O,q_t|HMDBN_i)$ for the hidden graphs with same colors on the left. **(a)** Transformation for the network with only one parent node. **(b)** Transformation for the network with multiple parent nodes.

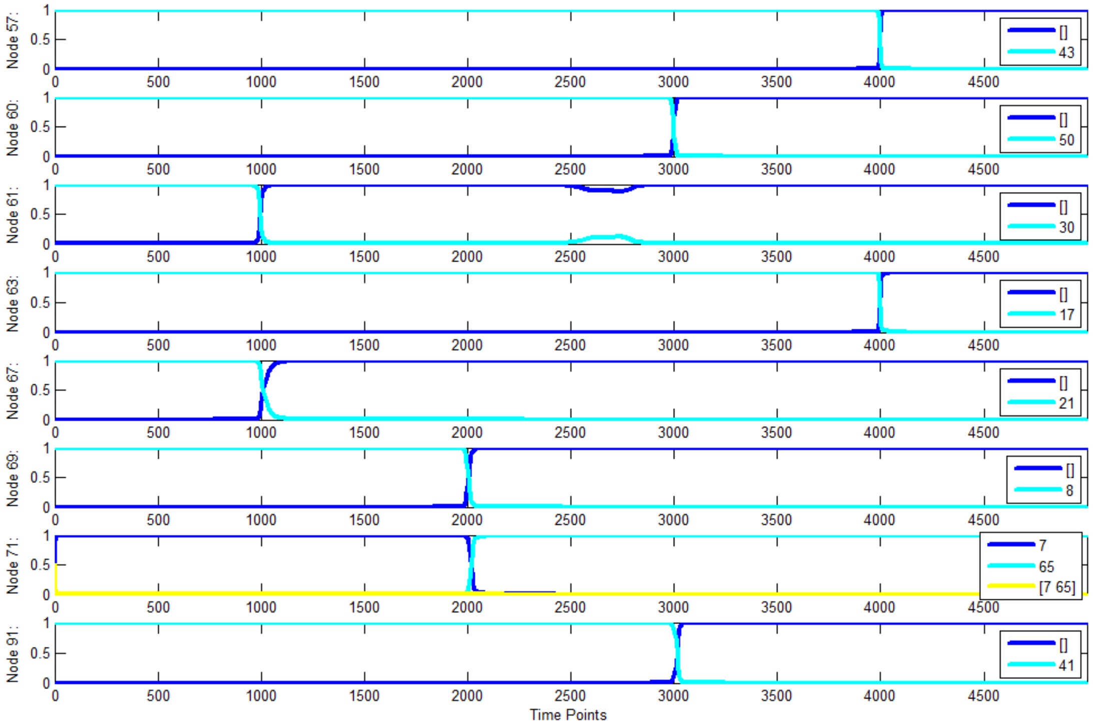

Supplementary Fig. 2. The $P(O,q_t|HMDBN)$ of one dataset simulated by a big network. The horizontal axis denotes time points; the vertical axis represents the probability density. The curves in different colors correspond to the $P(O,q_t|HMDBN)$ for different hidden graphs, respectively, whose parent nodes are given in the rectangles. The big network has 100 nodes with 50 edges over five time segments, and two edges changing between each epoch. The simulated dataset spans 5000 time points with transition times located at 1001, 2001, 3001, and 4001. Using *HMDBN*, we recovered all 50 true edges, and predicted 10 exceptional ones. In this figure, only the nodes are demonstrated, whose parent nodes evolve over time. It is shown that all of the predicted transition times are correct.

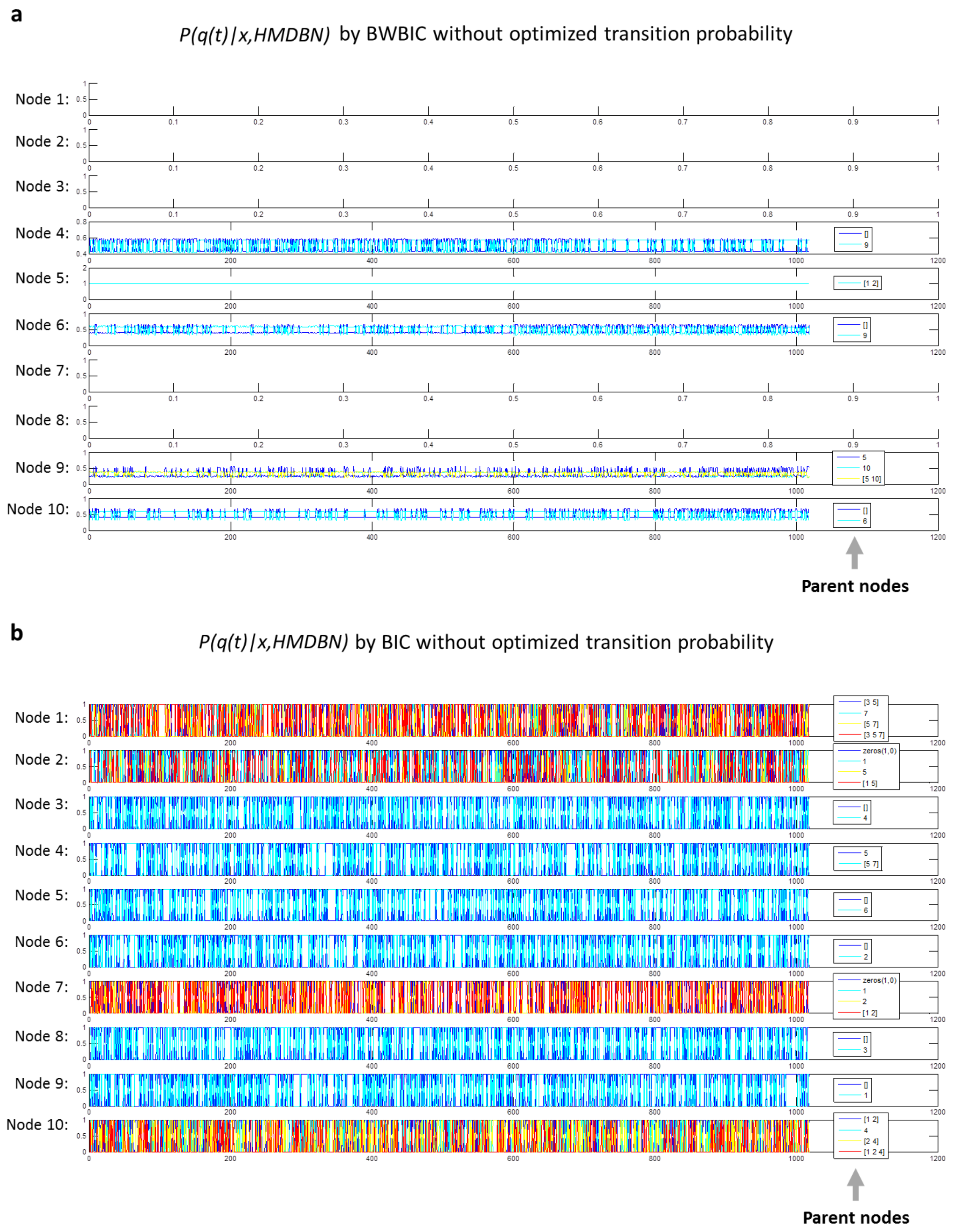
a
P(q(t)|x,HMDBN) by BWBIC without optimized transition probability
Node 1:
Node 2:
Node 3:
Node 4:
[]
9
Node 5:
[1 2]
Node 6:
[]
9
Node 7:
Node 8:
Node 9:
5
10
[5 10]
Node 10:
[]
6
Parent nodes
b
P(q(t)|x,HMDBN) by BIC without optimized transition probability
Node 1:
[3 5]
7
[5 7]
[3 5 7]
Node 2:
zeros(1,0)
1
5
[1 5]
Node 3:
[]
4
Node 4:
5
[5 7]
Node 5:
[]
6
Node 6:
[]
2
Node 7:
zeros(1,0)
1
2
[1 2]
Node 8:
[]
3
Node 9:
[]
1
Node 10:
[1 2]
4
[2 4]
[1 2 4]
Parent nodes

c

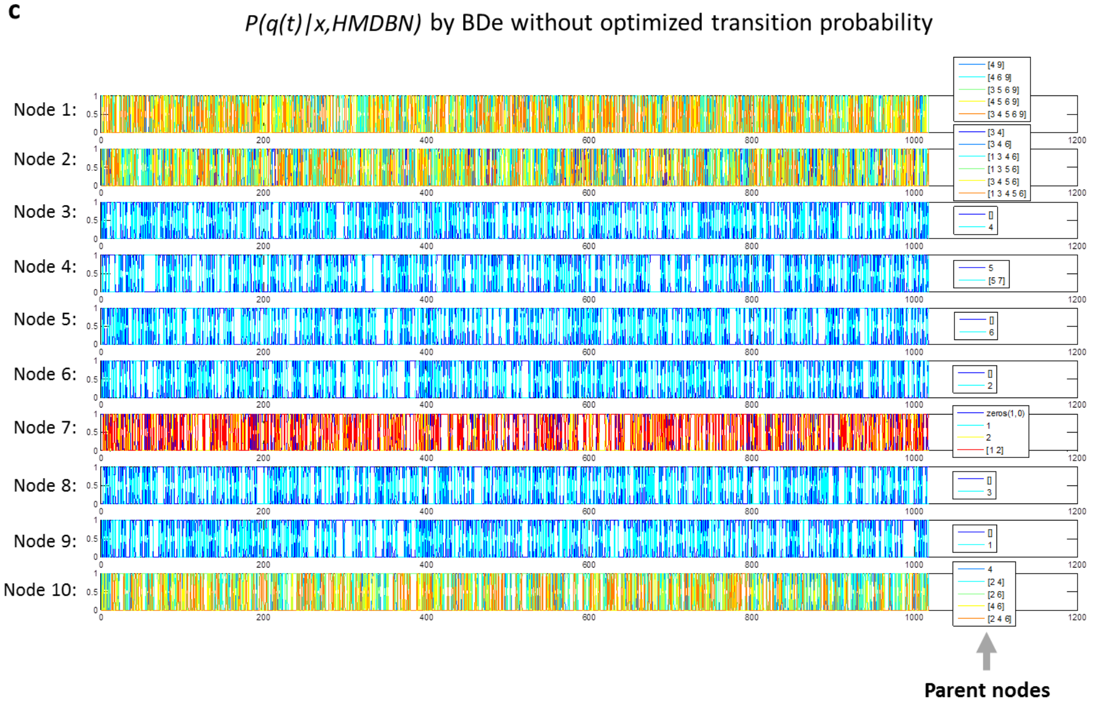

Supplementary Fig. 3. The *P(q(t)|x,HMDBN)* of one simulated dataset recovered by three metrics BWBIC, BIC and BDe without optimized transition probabilities. The horizontal axis denotes time points; the vertical axis represents the probability density. The curves in different colors correspond to the *P(q(t)|x,HMDBN)* for different hidden graphs, respectively, whose parent nodes are given in the rectangles. **(a)** *P(q(t)|x,HMDBN)* recovered by BWBIC without optimized transition probabilities. **(b)** *P(q(t)|x,HMDBN)* recovered by BIC without optimized transition probabilities. **(c)** *P(q(t)|x,HMDBN)* recovered by BDe without optimized transition probabilities. Due to no iterative re-estimation of transition probabilities, we did not predict clear transition times using all BWBIC, BIC and BDe scores. Therefore, the curves in different colors mix together in **(a-c)**. However, as shown in the rectangles of **(a-c)**, most of edges predicted by BWBIC are correct (**a**), while most of edges predicted by both BIC and BDe are false (**b-c**). This fact suggests that the BWBIC score can help obtain higher and more stable prediction accuracy when compared to BIC and BDe scores.

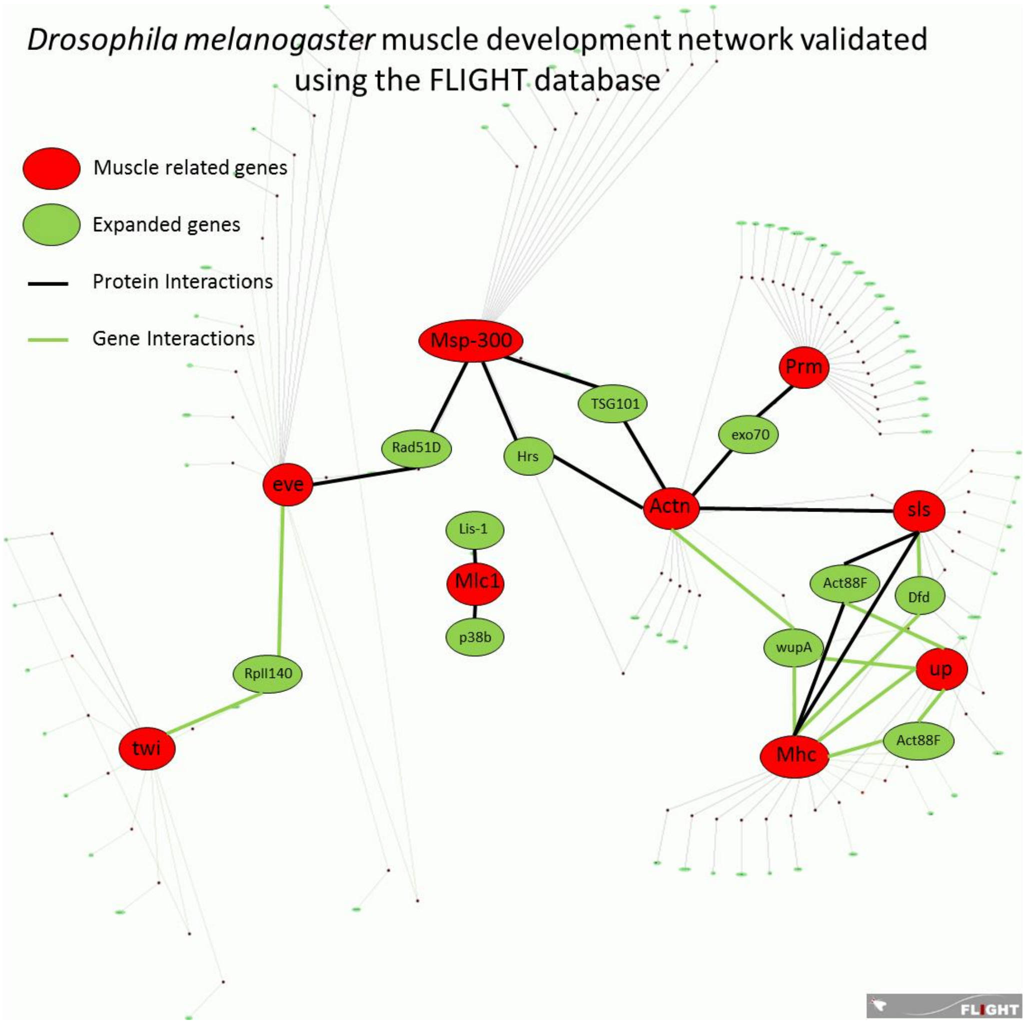

Supplementary Fig. 4. The FLIGHT-recorded gene interactions among *Drosophila* muscle-related genes, which are validated by existing biological experiments. To build this gold standard network, we first transformed 11 gene symbols (eve, gfl/lmd, twi, mlc1, sls, mhc, prm, actn, up, myo61f and msp300) into CG gene identifiers (CG2328, CG4677, CG2956, CG5596, CG1915, CG17927, CG5939, CG4376, CG7107, CG9155 and CG18251), and next, directly submitted them to the FLIGHT database (http://flight.icr.ac.uk/search/search_interactions.jsp) to search for both physical and genetic interactions. The background is the screen shot from FLIGHT database. For cleairity, the muscle-realted genes and intermediate genes were enlarged, and the edges between these genes were thickened.

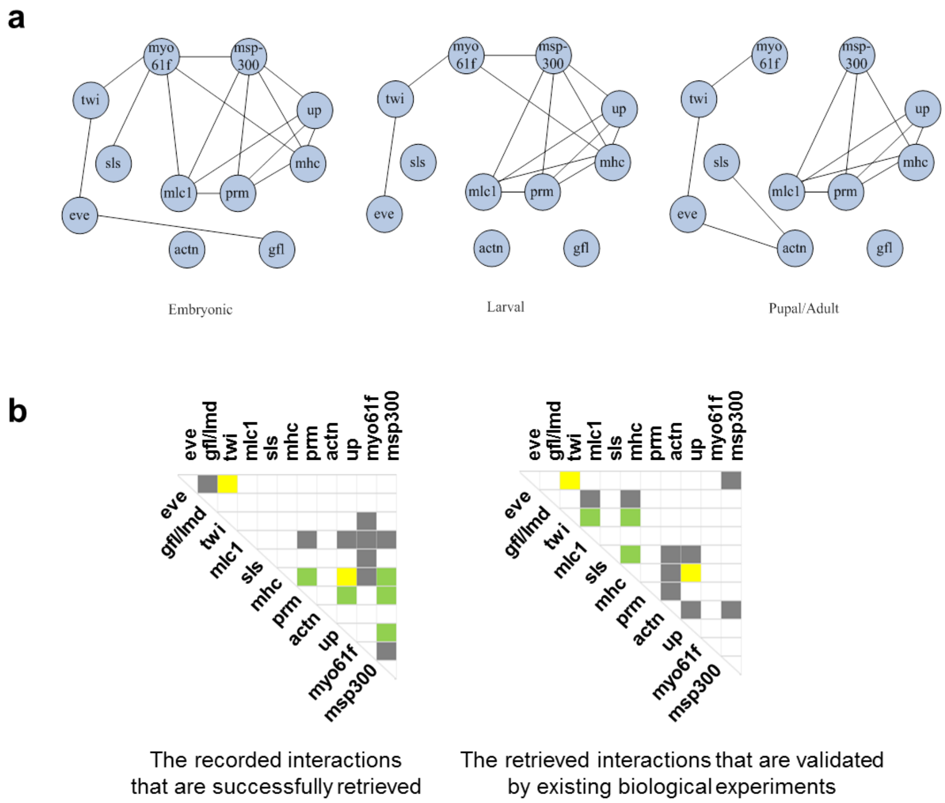

Supplementary Fig. 5. The predicted *Drosophila* muscle-related gene interaction network by the undirected non-stationary graph method *htEGRMs* [4]. **(a)** The time-varying gene interaction networks with 3 time segments predicted by *htERGMs*. **(b)** The retrieved interactions by *htEGRMs* and the interactions validated by existing biological experiments.

Reference:

1. Robinson J, Hartemink A. Non-Stationary Dynamic Bayesian Networks. Neural Information Processing Systems 2008 (NIPS 2008). 2008, 1369-1376.
2. Robinson JW, Hartemink AJ. Learning Non-Stationary Dynamic Bayesian Networks, Journal of Machine Learning Research 2010;11:3647-3680.
3. Lebre S, Becq J, Devaux F et al. Statistical inference of the time-varying structure of gene-regulation networks, BMC Syst Biol 2010;4:130.
4. Guo F, Hanneke S, Fu W et al. Recovering temporally rewiring networks: A model-based approach. Proceedings of the 24th International Conference on Machine Learning (ICML 2007). 2007, 321-328.

Supplementary File 2-3:

Prediction results of *ARTIVA* and *nhDBN*.

The prediction by *nsDBN* for both simulated and real biological datasets directly refers to the original paper.

Supplementary File 2. Prediction results of *ARTIVA* on the simulated dataset and real biological gene expression dataset under different parameter settings. The networks are the original output by the *ARTIVA* package.

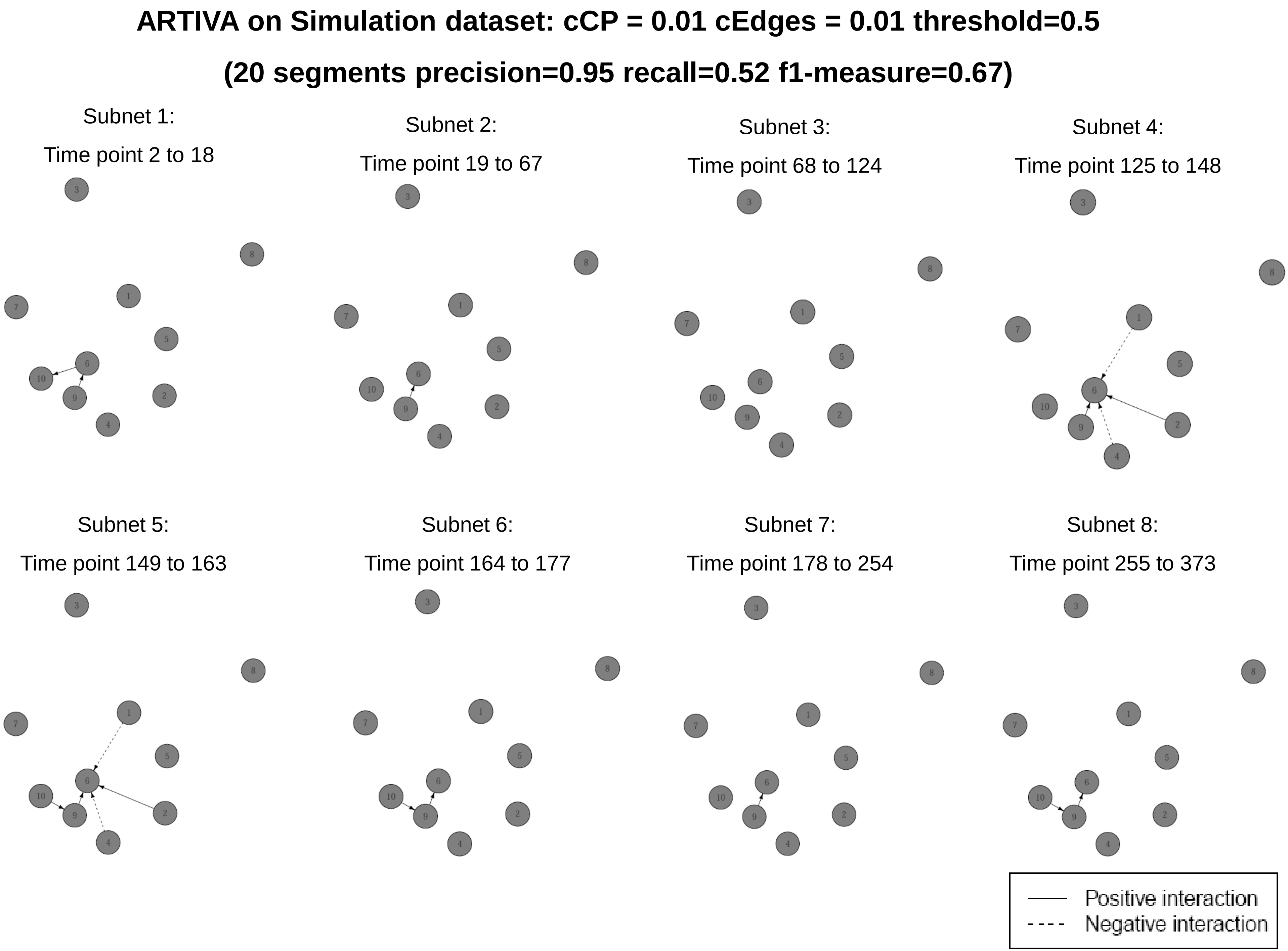
ARTIVA on Simulation dataset: cCP = 0.01 cEdges = 0.01 threshold=0.5
(20 segments precision=0.95 recall=0.52 f1-measure=0.67)
Subnet 1:
Time point 2 to 18
Subnet 2:
Time point 19 to 67
Subnet 3:
Time point 68 to 124
Subnet 4:
Time point 125 to 148
Subnet 5:
Time point 149 to 163
Subnet 6:
Time point 164 to 177
Subnet 7:
Time point 178 to 254
Subnet 8:
Time point 255 to 373
Positive interaction
Negative interaction

ARTIVA on Simulation dataset: cCP = 0.01 cEdges = 0.01 threshold=0.5

(20 segments precision=0.95 recall=0.52 f1-measure=0.67)

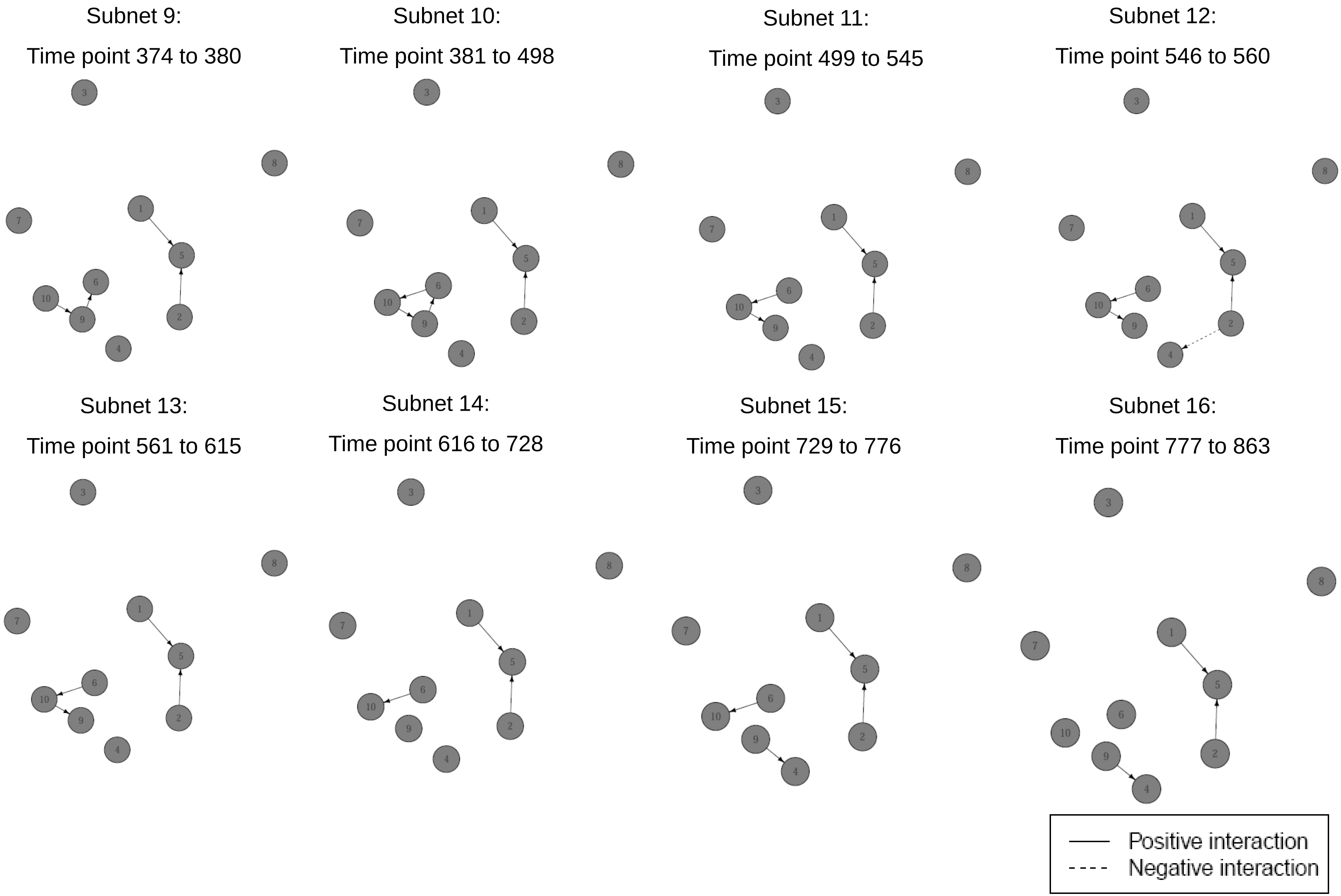

ARTIVA on Simulation dataset: cCP = 0.01 cEdges = 0.01 threshold=0.5

(20 segments precision=0.95 recall=0.52 f1-measure=0.67)

Subnet 17:

Time point 864 to 884

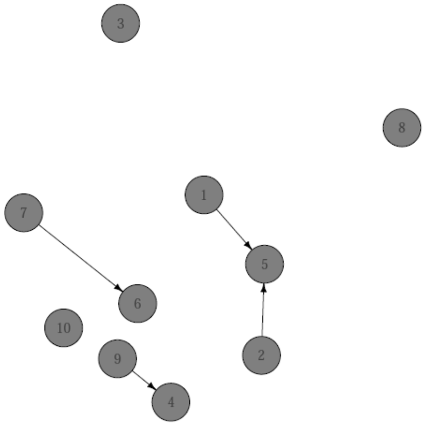

Subnet 18:

Time point 885 to 952

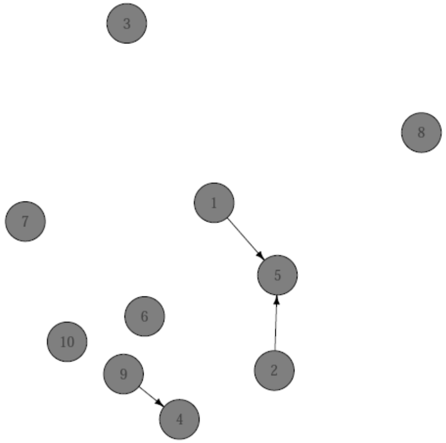

Subnet 19:

Time point 953 to 976

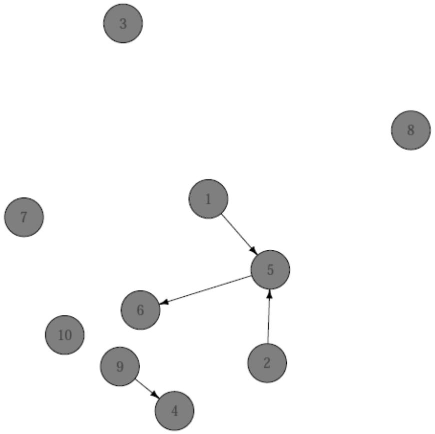

Subnet 20:

Time point 954 to 1019

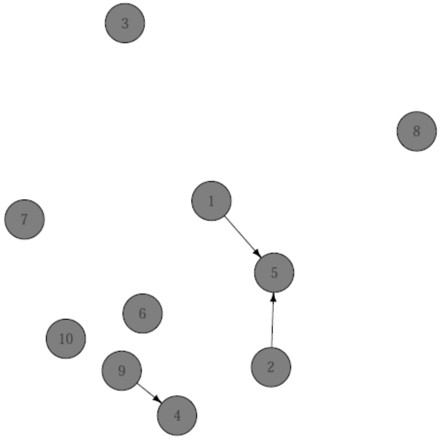

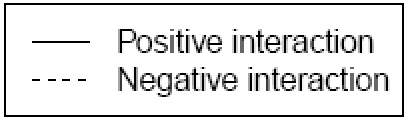

ARTIVA on Simulation dataset: cCP = 0.1 cEdges = 0.1 threshold=0.5

(6 segments precision=0.99 recall=0.66 f1-measure=0.79)

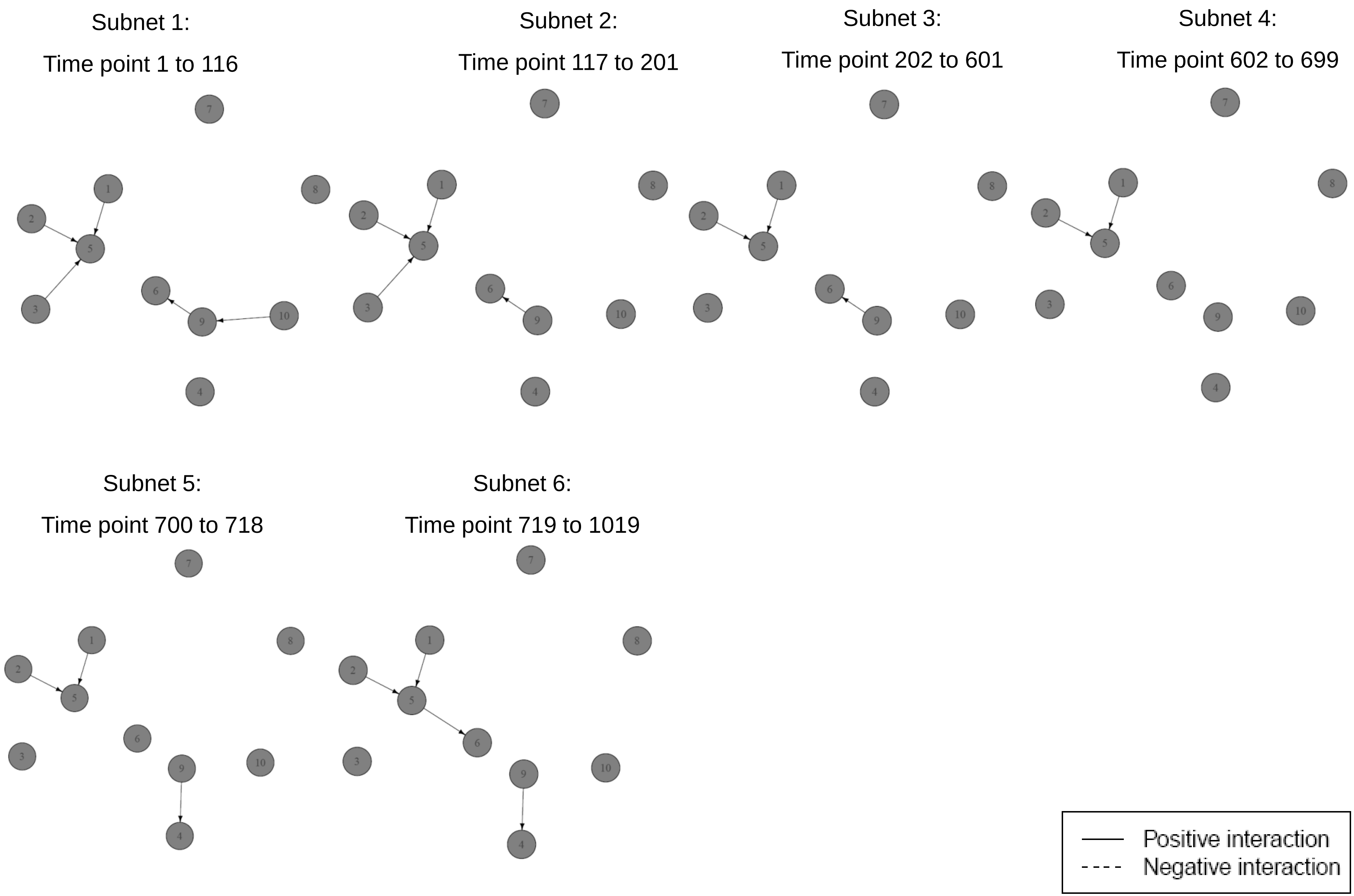

ARTIVA on Simulation dataset: cCP = 0.5 cEdges = 0.5 threshold=0.5

(7 segments precision=0.99 recall=0.97 f1-measure=0.98)

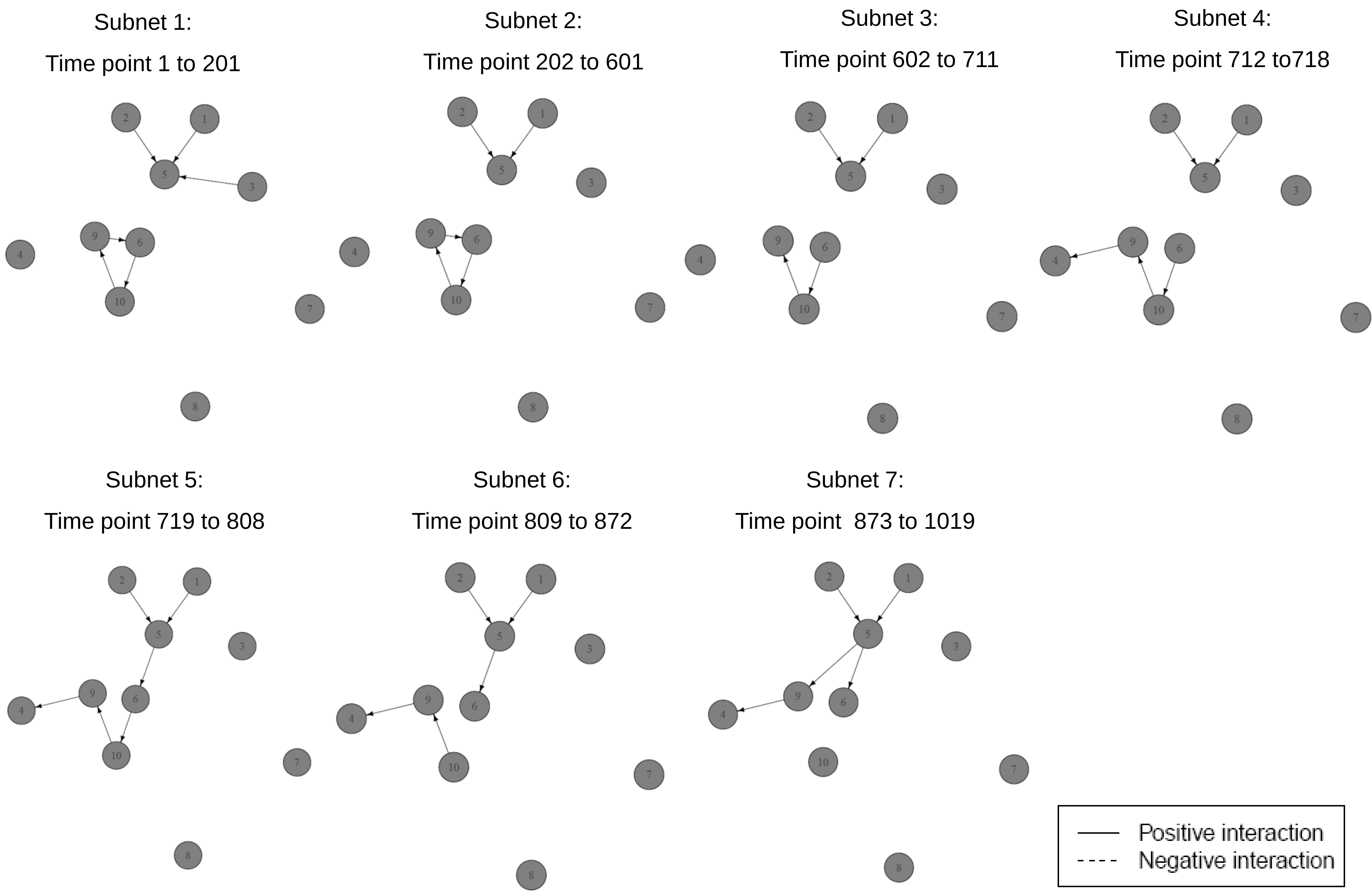

ARTIVA on Simulation dataset: cCP = 0.8 cEdges = 0.8 threshold=0.5

(7 segments precision=0.99 recall=0.97 f1-measure=0.98)

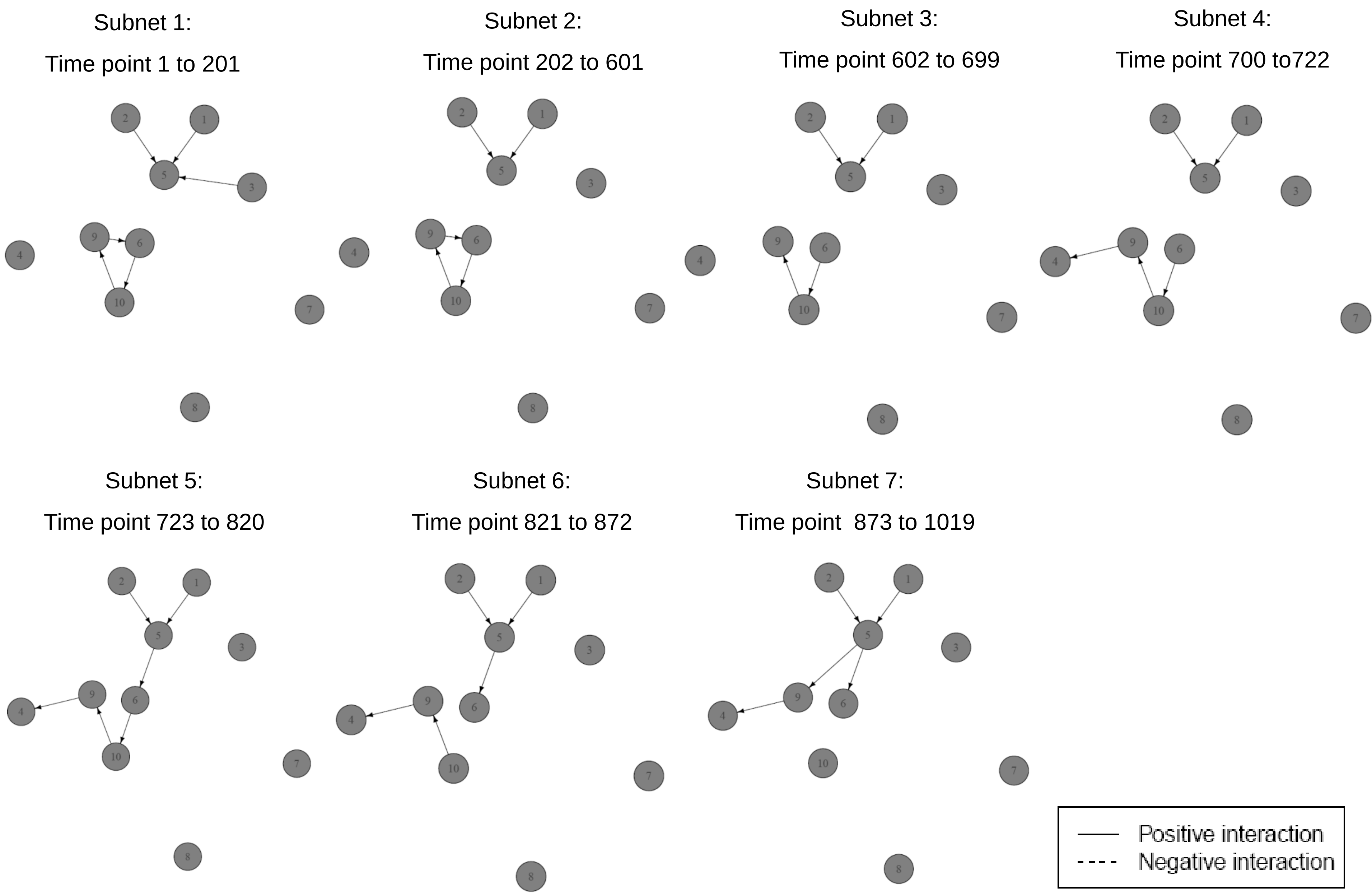

ARTIVA on Simulation dataset: cCP = 0.9 cEdges = 0.5 threshold=0.5

(7 segments precision=0.48 recall=0.99 f1-measure=0.65)

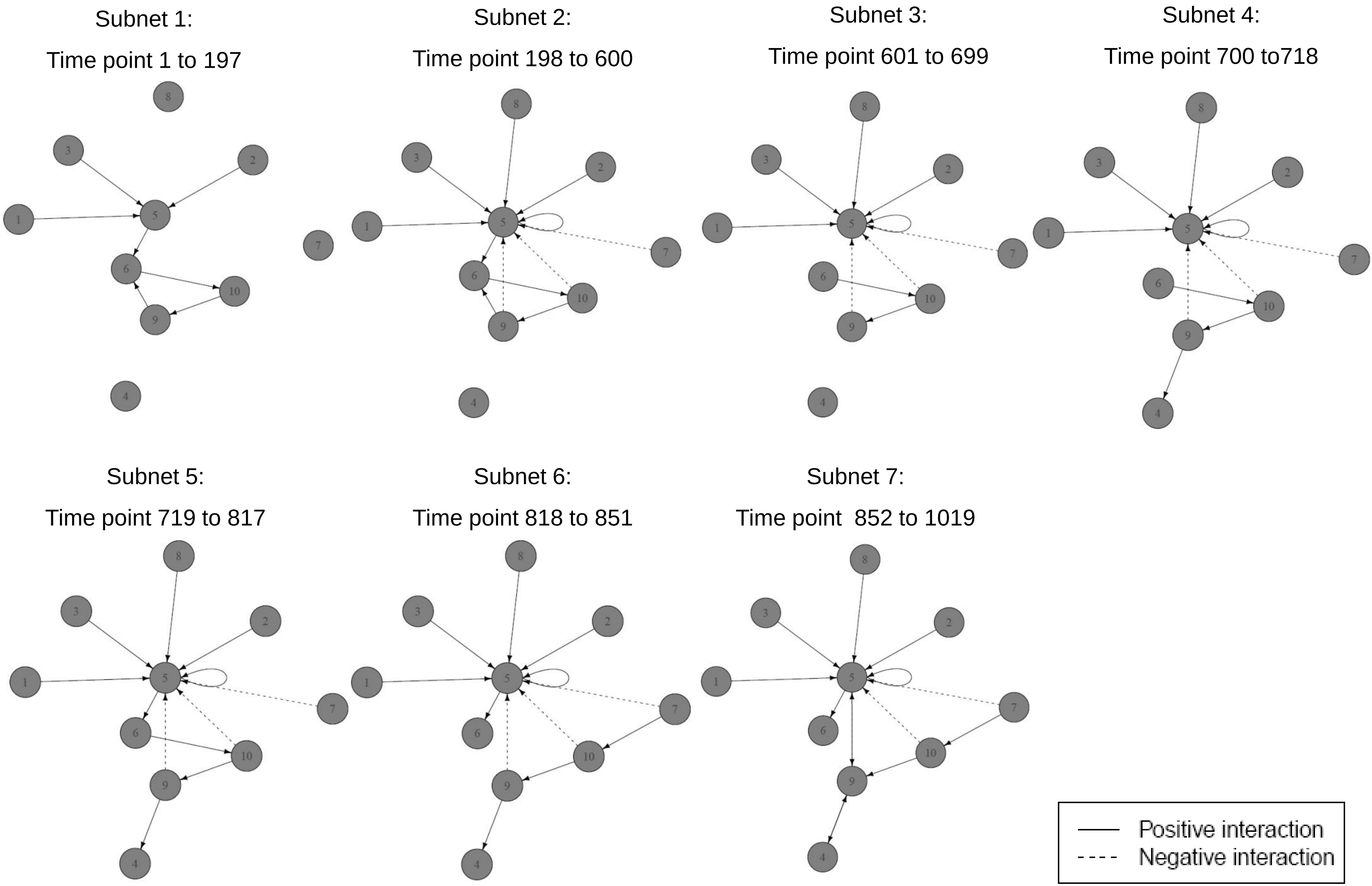

ARTIVA on Simulation dataset: cCP = 1 cEdges = 1 threshold=0.5

(10 segments precision=0.41 recall=0.97 f1-measure=0.58)

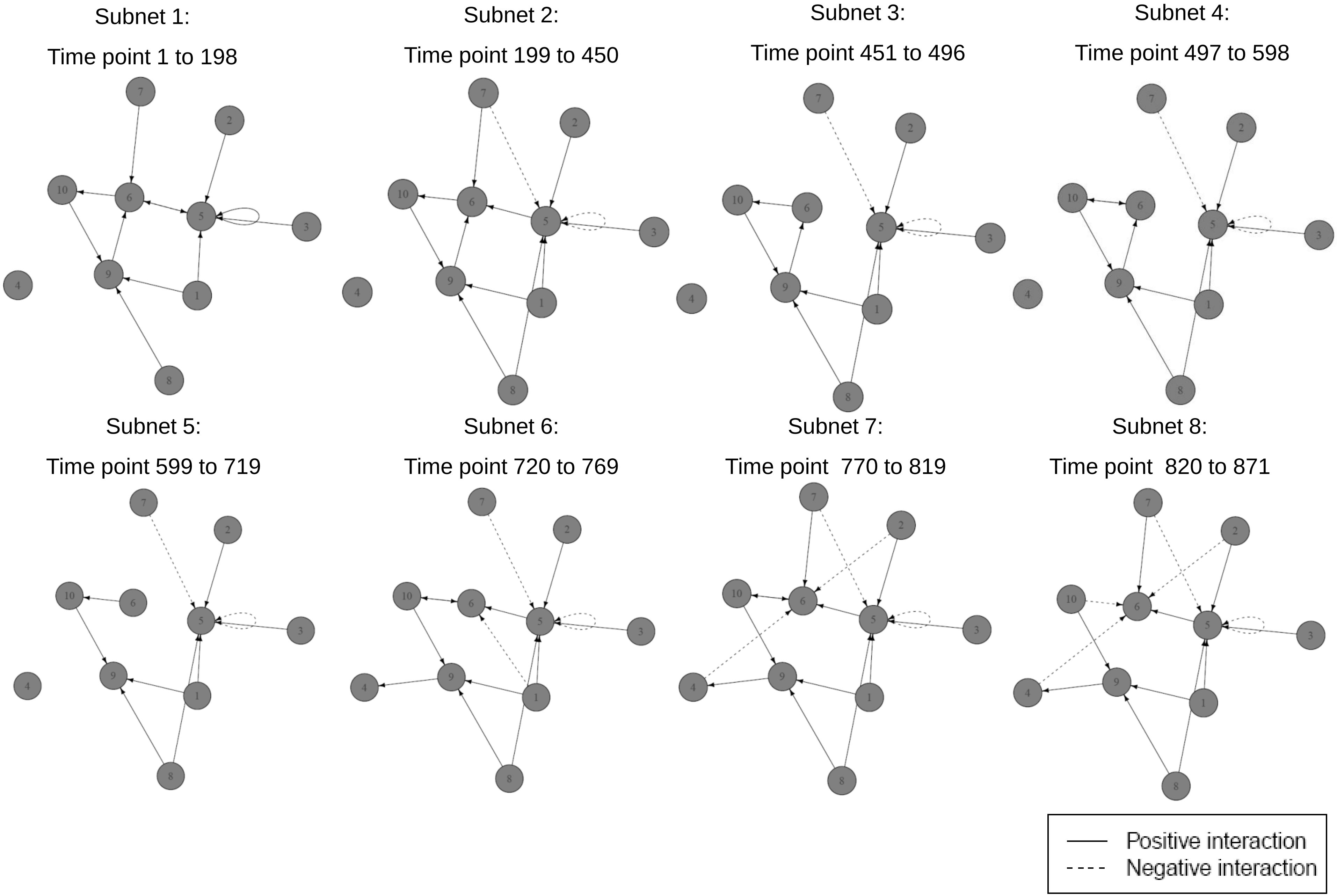

ARTIVA on Simulation dataset: cCP = 1 cEdges = 1 threshold=0.5

(10 segments precision=0.41 recall=0.97 f1-measure=0.58)

Subnet 9:

Time point 872 to 1006

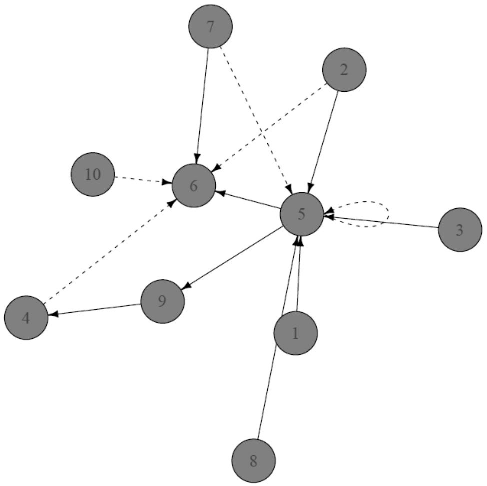

Subnet 10:

Time point 1007 to 1019

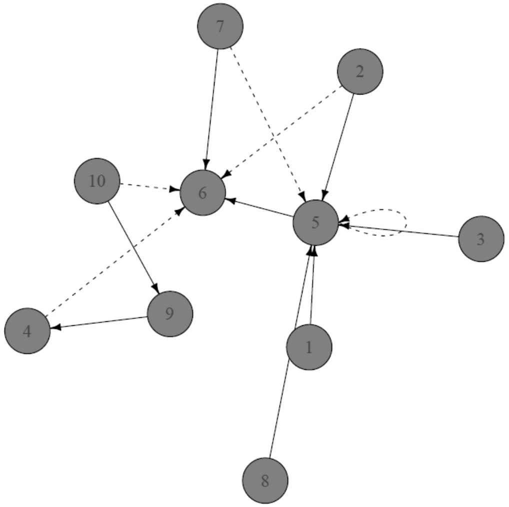

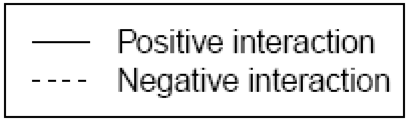

ARTIVA on Drosophila muscle-related discrete gene expression dataset

cCP = 0.1 cEdges = 0.1 threshold=0.5

Subnet 1:

Time point 1 to 24

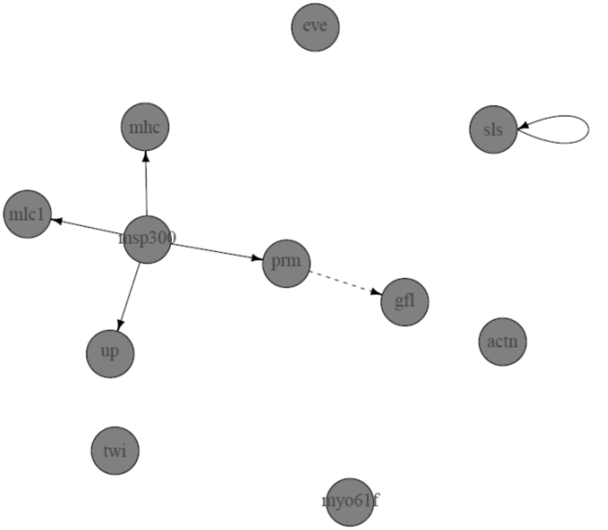

Subnet 2:

Time point 25 to 26

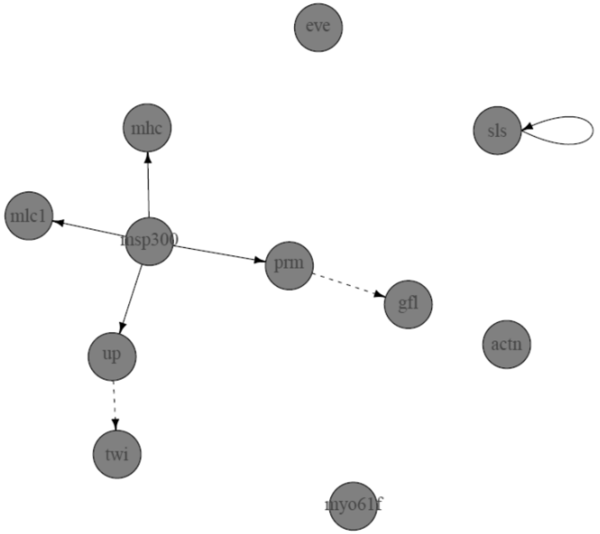

Subnet 3:

Time point 27 to 37

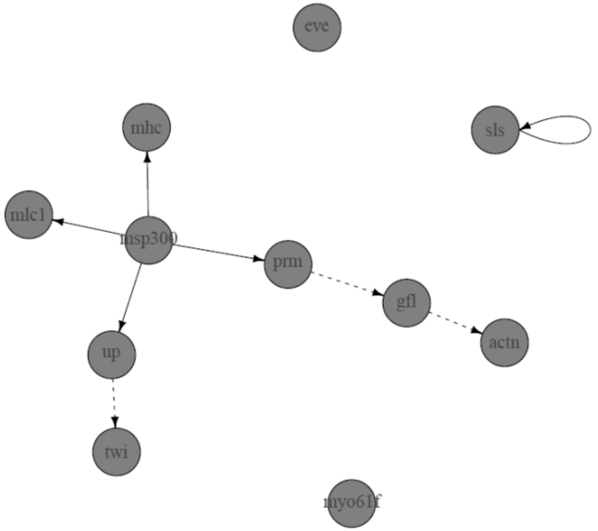

Subnet 4:

Time point 38 to 40

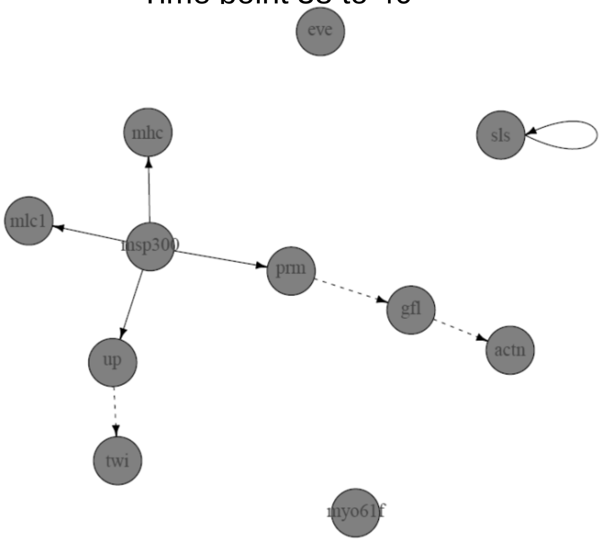

Subnet 5:

Time point 41 to 41

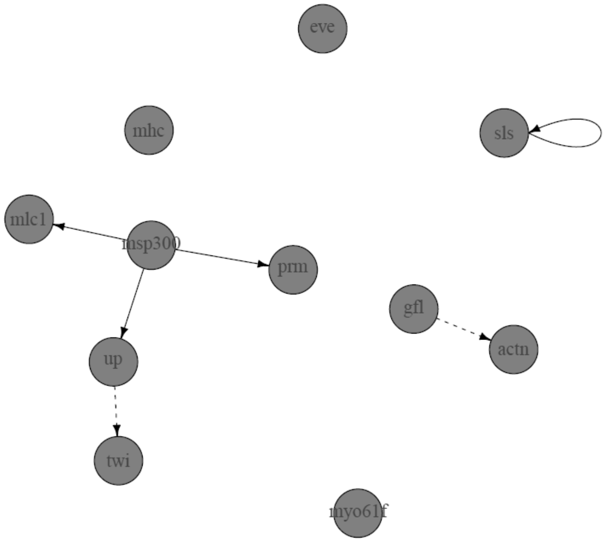

Subnet 6:

Time point 42 to 48

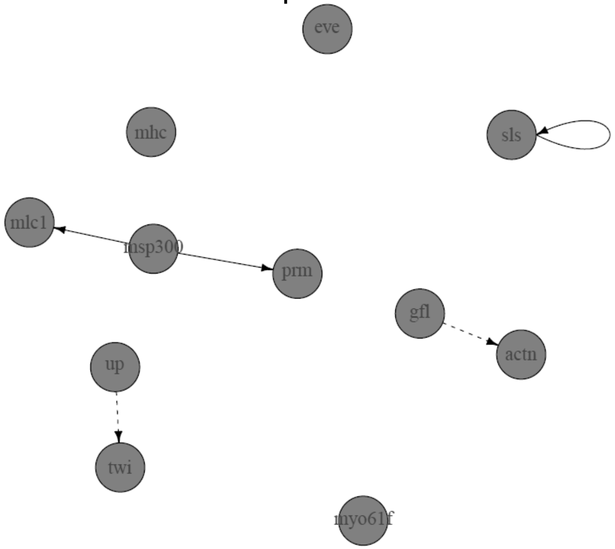

ARTIVA on Drosophila muscle-related discrete gene expression dataset

cCP = 0.1 cEdges = 0.1 threshold=0.5

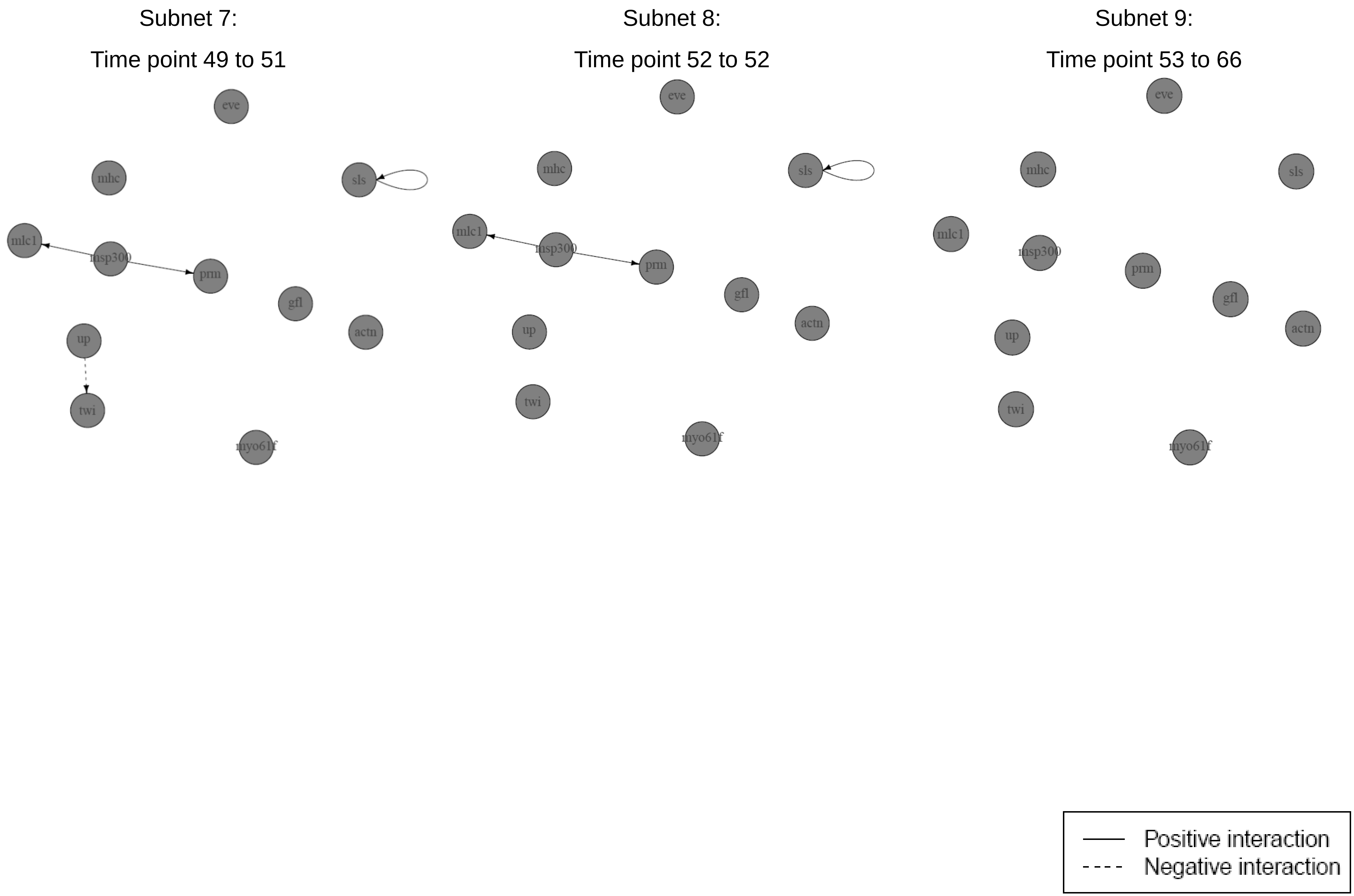

ARTIVA on Drosophila muscle-related discrete gene expression dataset

cCP = 0.2 cEdges = 0.2 threshold=0.5

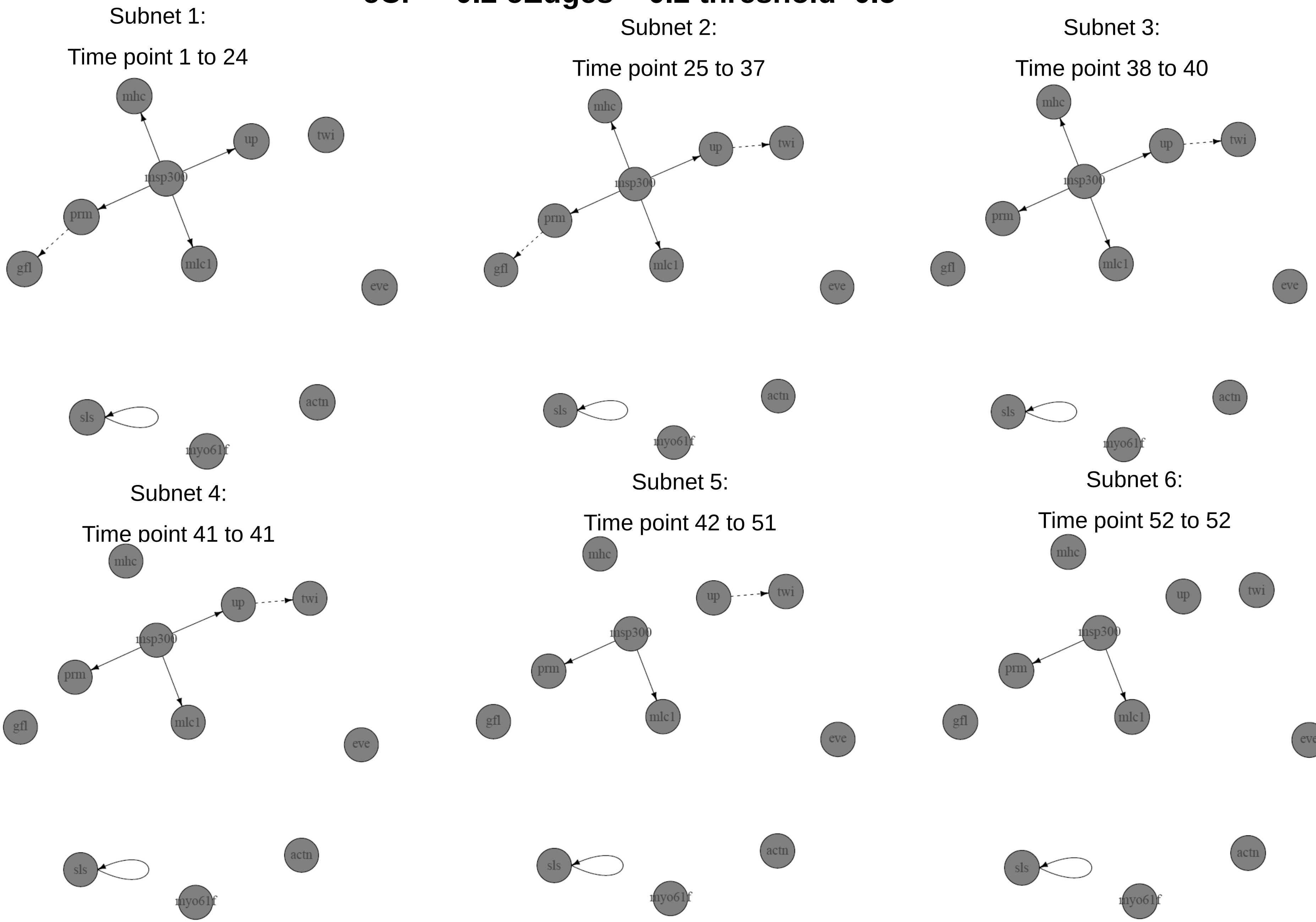

ARTIVA on Drosophila muscle-related discrete gene expression dataset

cCP = 0.2 cEdges = 0.2 threshold=0.5

Subnet 7:

Time point 53 to 66

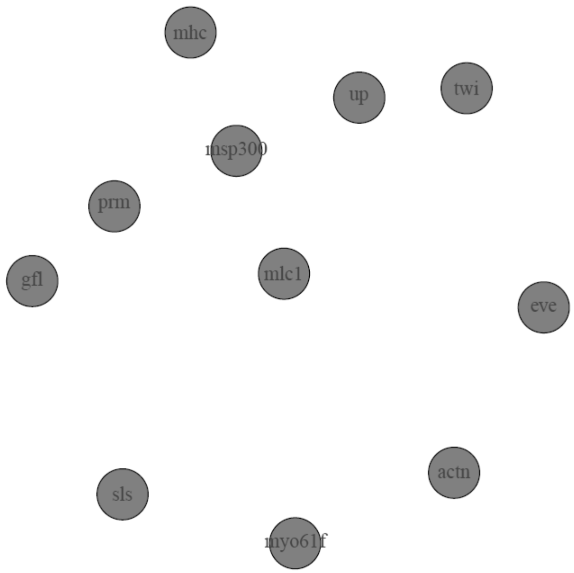

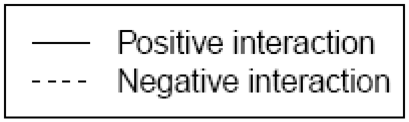

ARTIVA on Drosophila muscle-related discrete gene expression dataset

cCP = 0.3 cEdges = 0.3 threshold=0.5

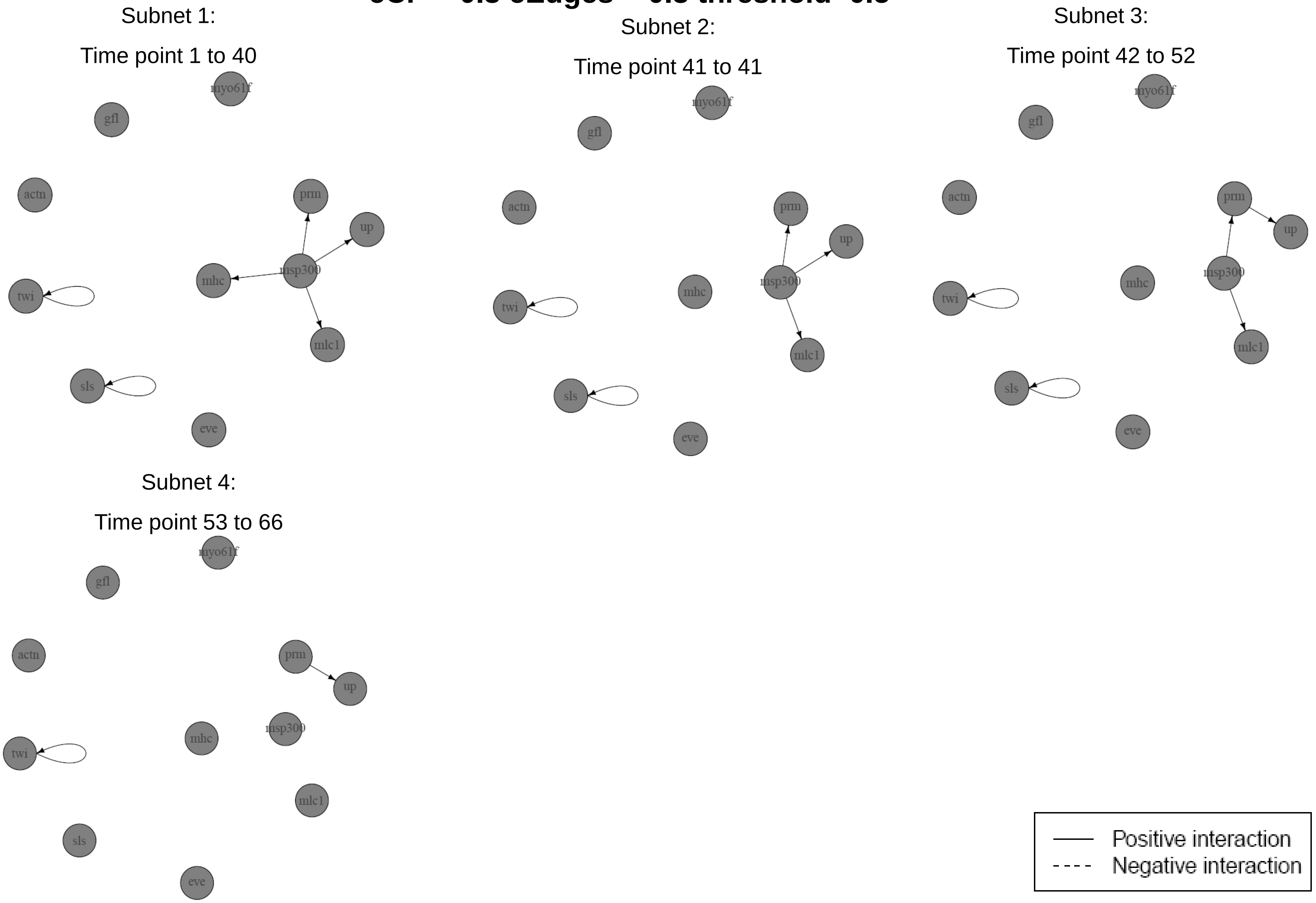

ARTIVA on Drosophila muscle-related discrete gene expression dataset

cCP = 0.4 cEdges = 0.4 threshold=0.5

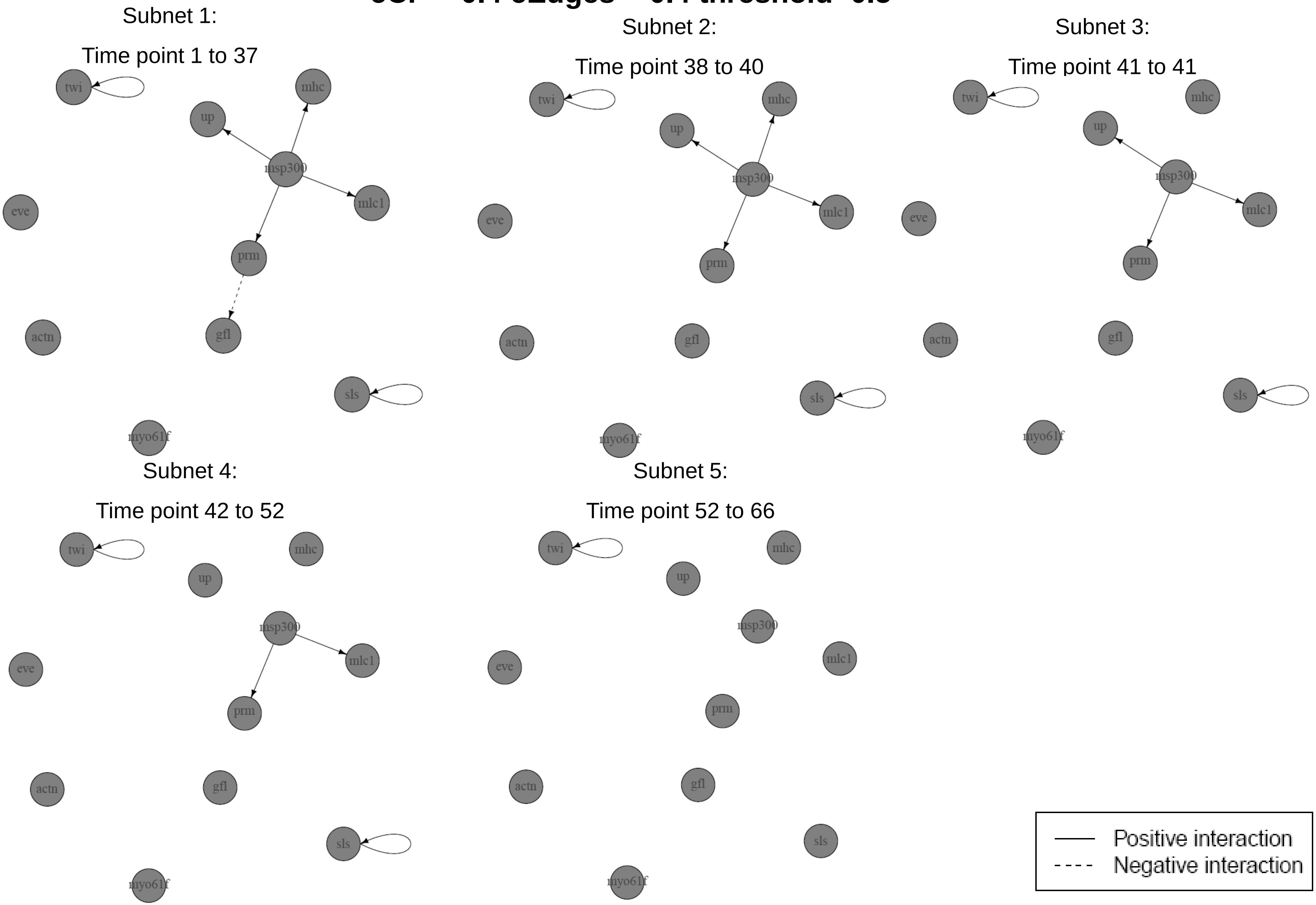

ARTIVA on Drosophila muscle-related discrete gene expression dataset

cCP = 0.5 cEdges = 0.5 threshold=0.5

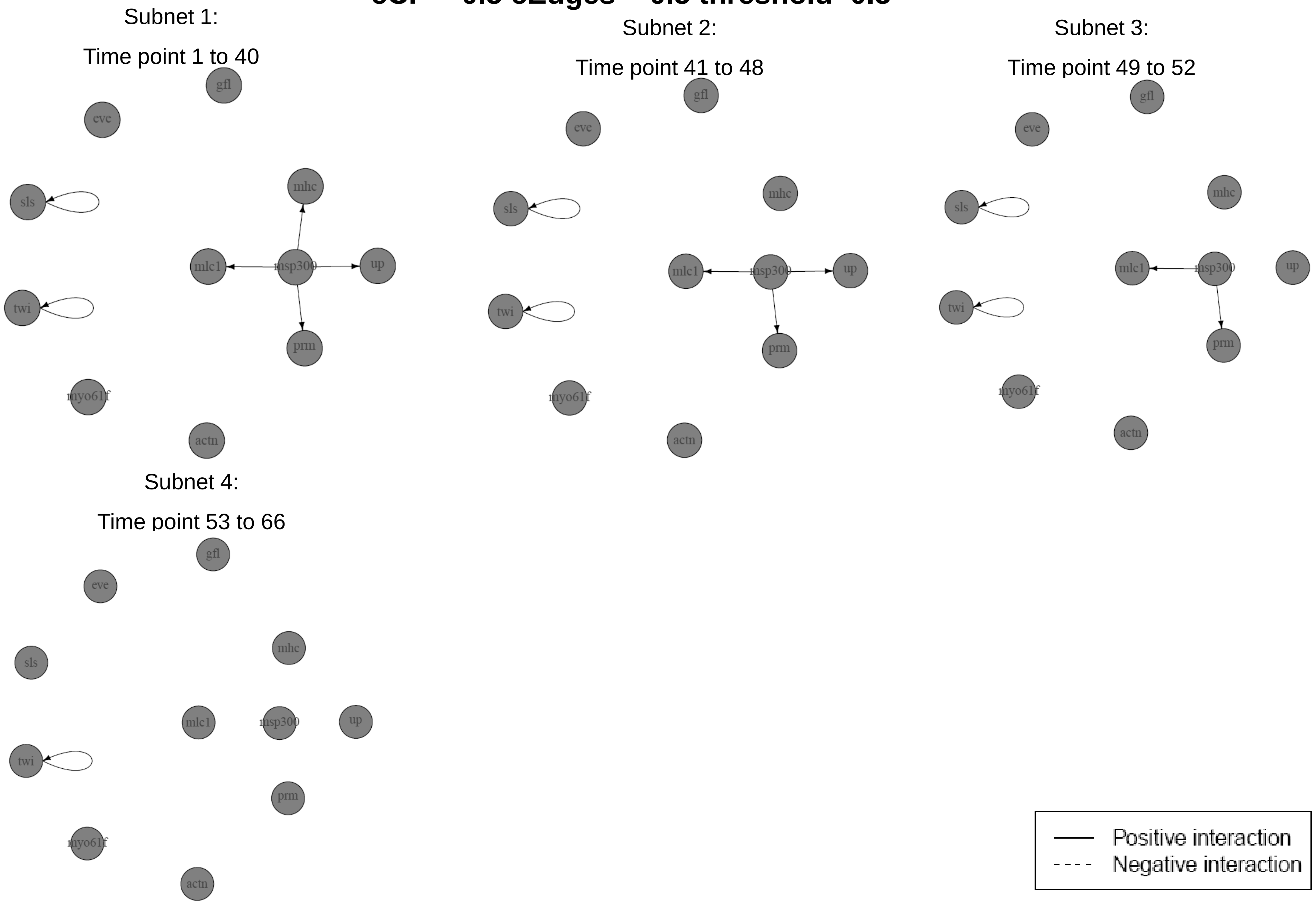

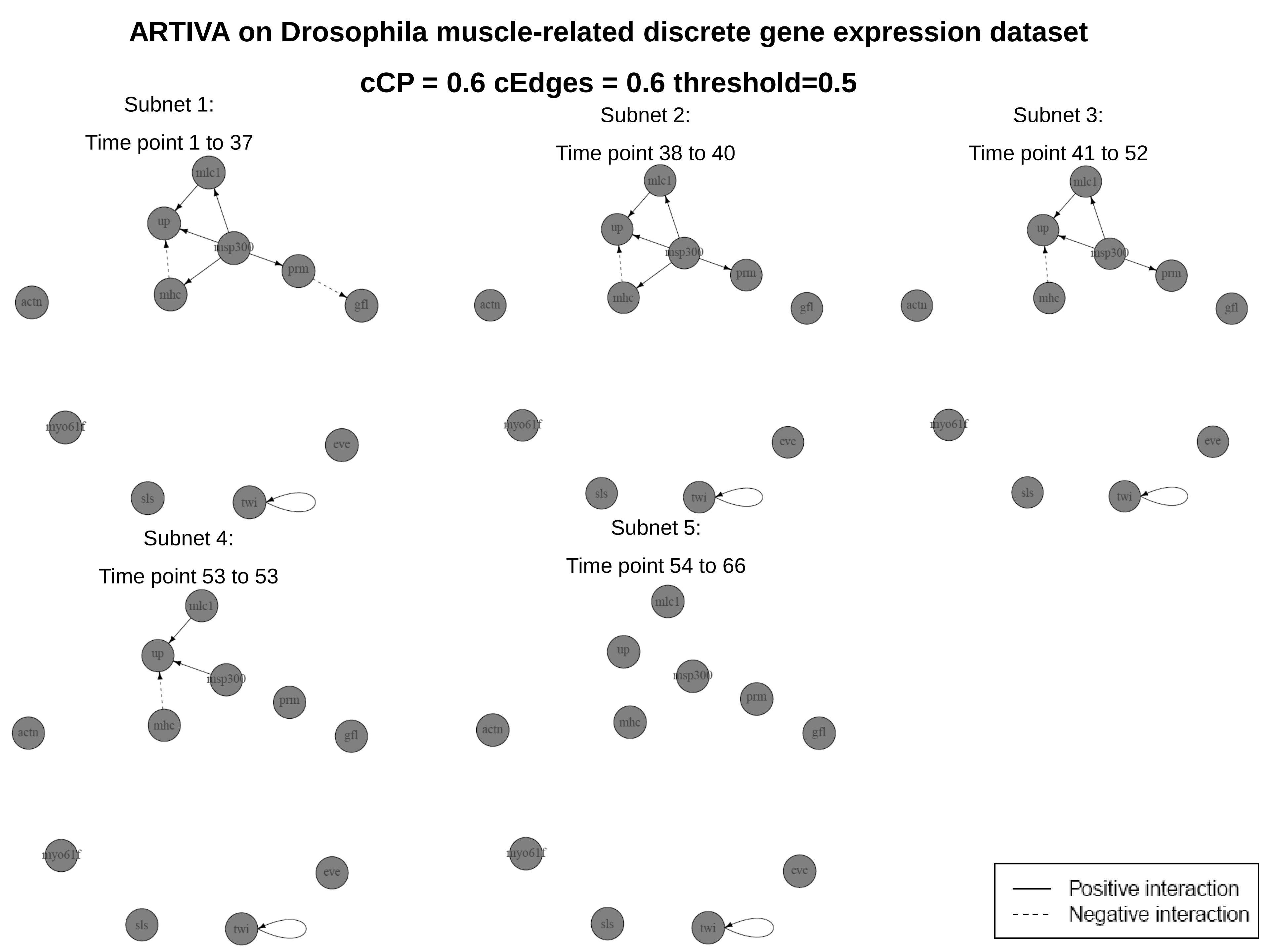

ARTIVA on Drosophila muscle-related discrete gene expression dataset
cCP = 0.6 cEdges = 0.6 threshold=0.5
Subnet 1:
Time point 1 to 37
Subnet 2:
Time point 38 to 40
Subnet 3:
Time point 41 to 52
Subnet 4:
Time point 53 to 53
Subnet 5:
Time point 54 to 66
mlc1
up
msp300
prm
mhc
gfl
actn
myo61f
eve
sls
twi
Positive interaction
Negative interaction

ARTIVA on Drosophila muscle-related discrete gene expression dataset

cCP = 0.7 cEdges = 0.7 threshold=0.5

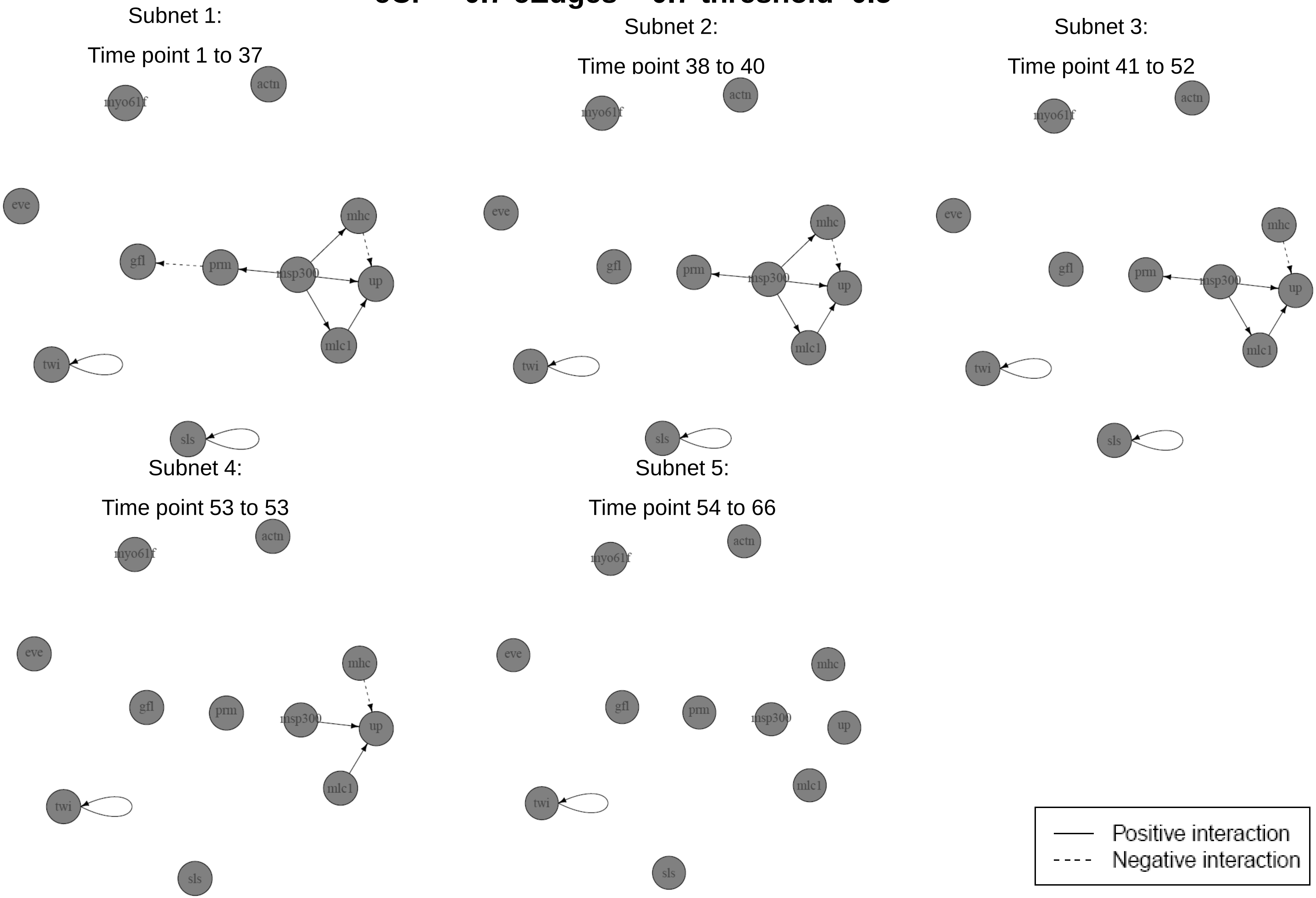

ARTIVA on Drosophila muscle-related discrete gene expression dataset

cCP = 0.8 cEdges = 0.8 threshold=0.5

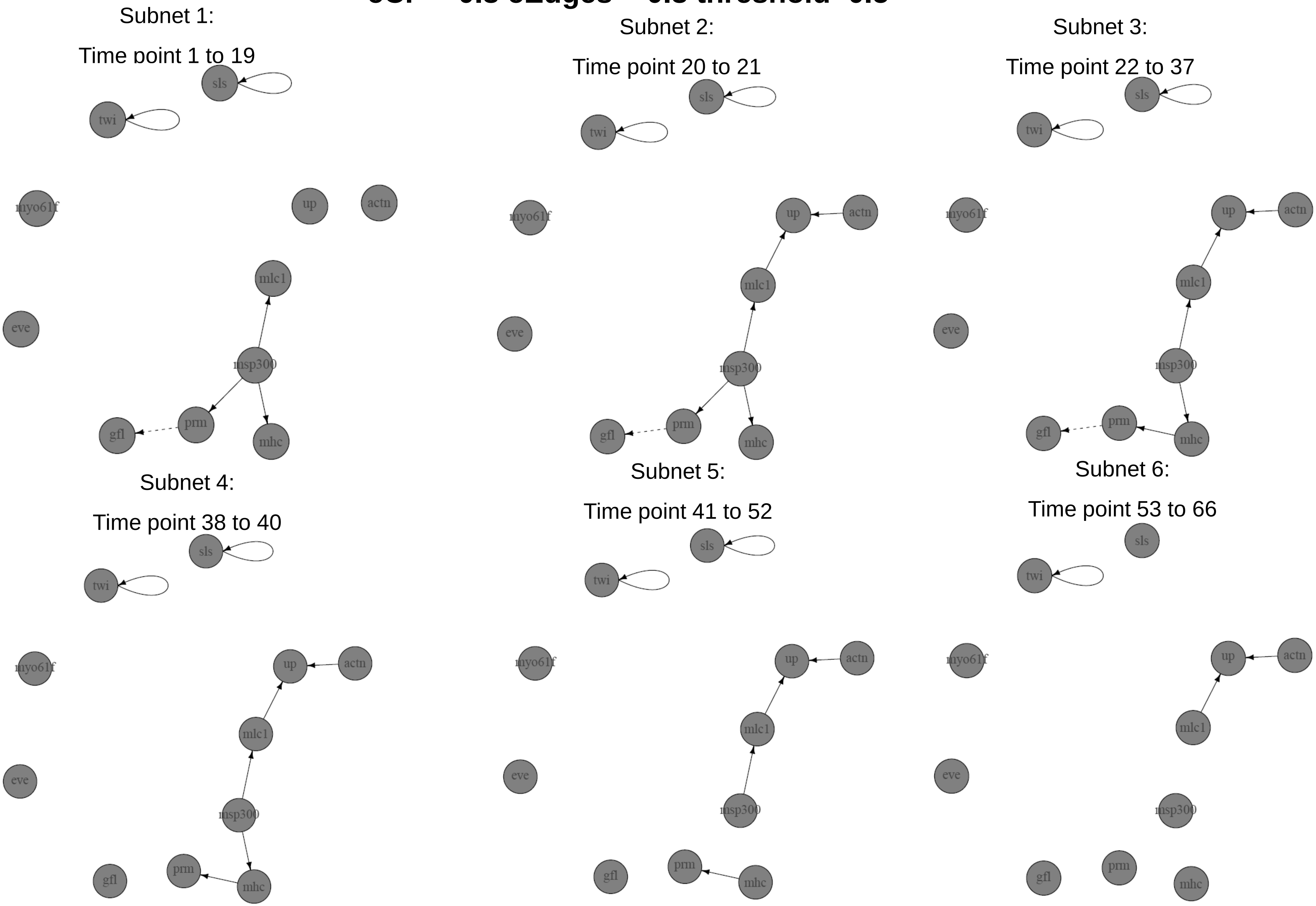

ARTIVA on Drosophila muscle-related discrete gene expression dataset

cCP = 0.9 cEdges = 0.9 threshold=0.5

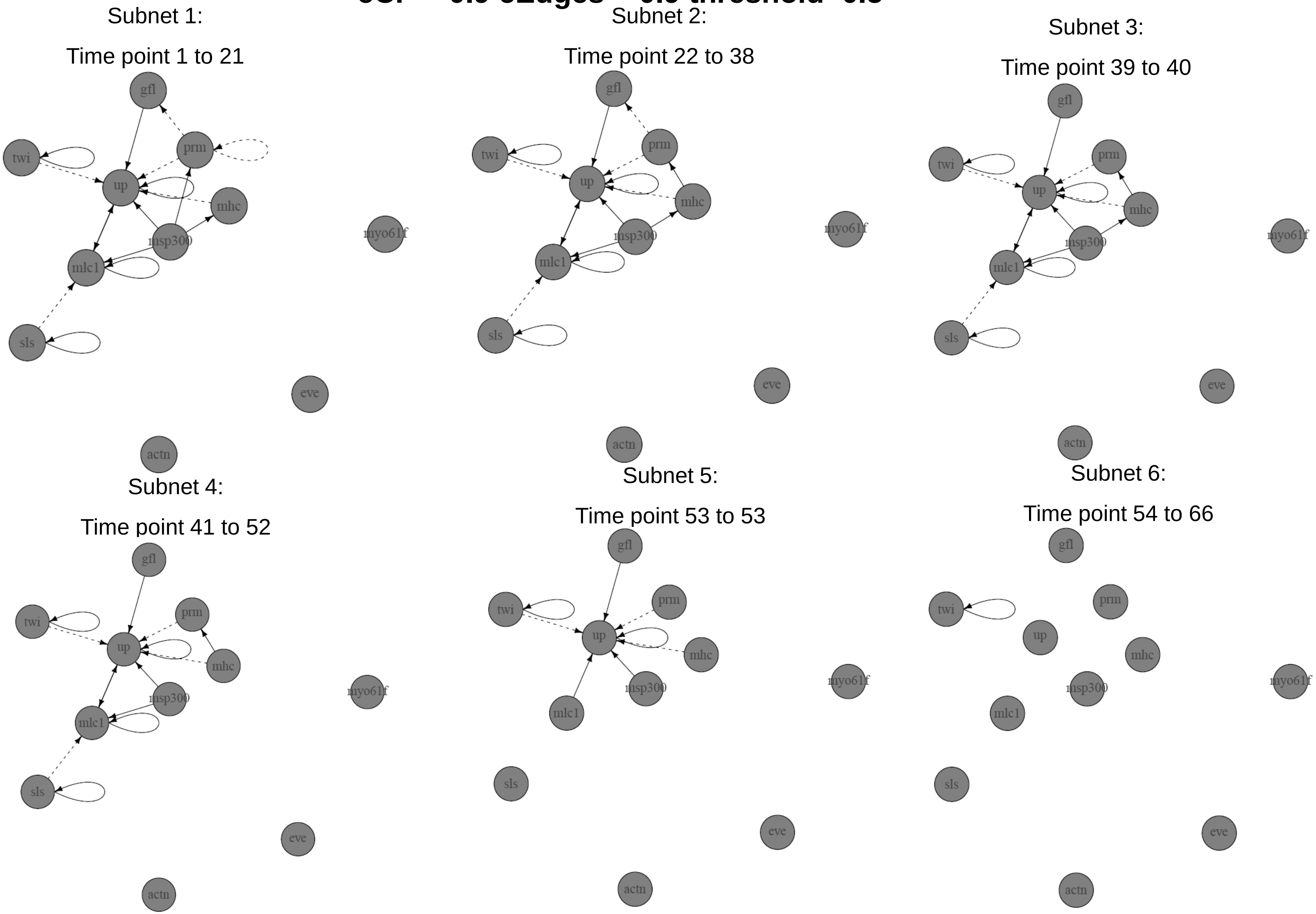

ARTIVA on Drosophila muscle-related continuous gene expression dataset

cCP = 0.1 cEdges = 0.1 threshold=0.5

Subnet 1:

Time point 1 to 66

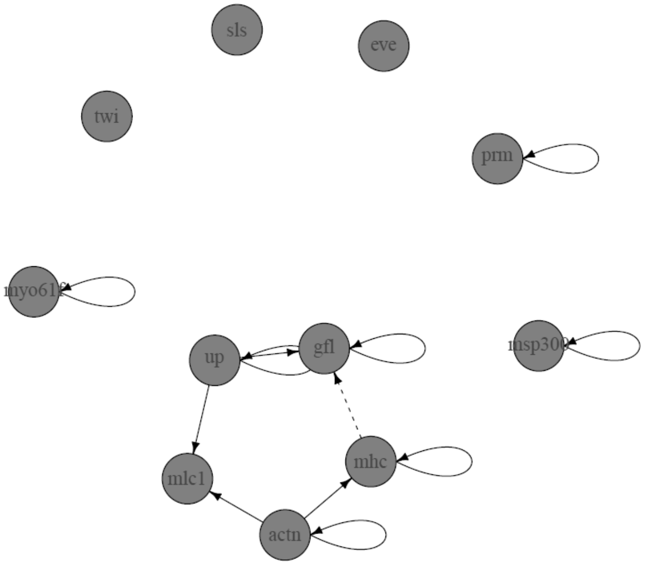

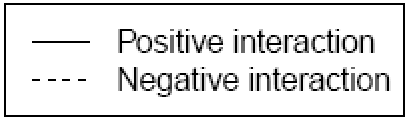

ARTIVA on Drosophila muscle-related continuous gene expression dataset

cCP = 0.2 cEdges = 0.2 threshold=0.5

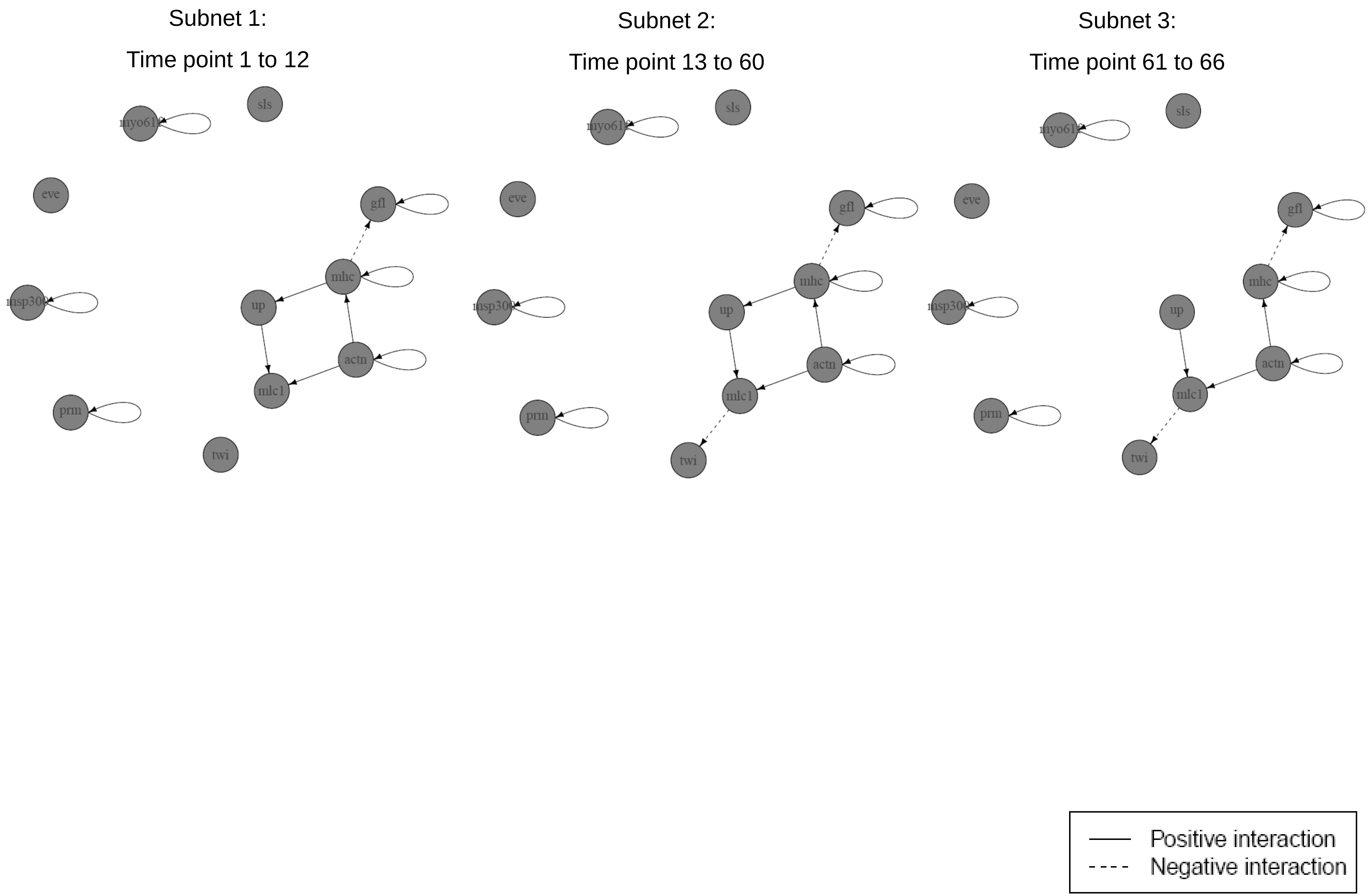

ARTIVA on Drosophila muscle-related continuous gene expression dataset

cCP = 0.3 cEdges = 0.3 threshold=0.5

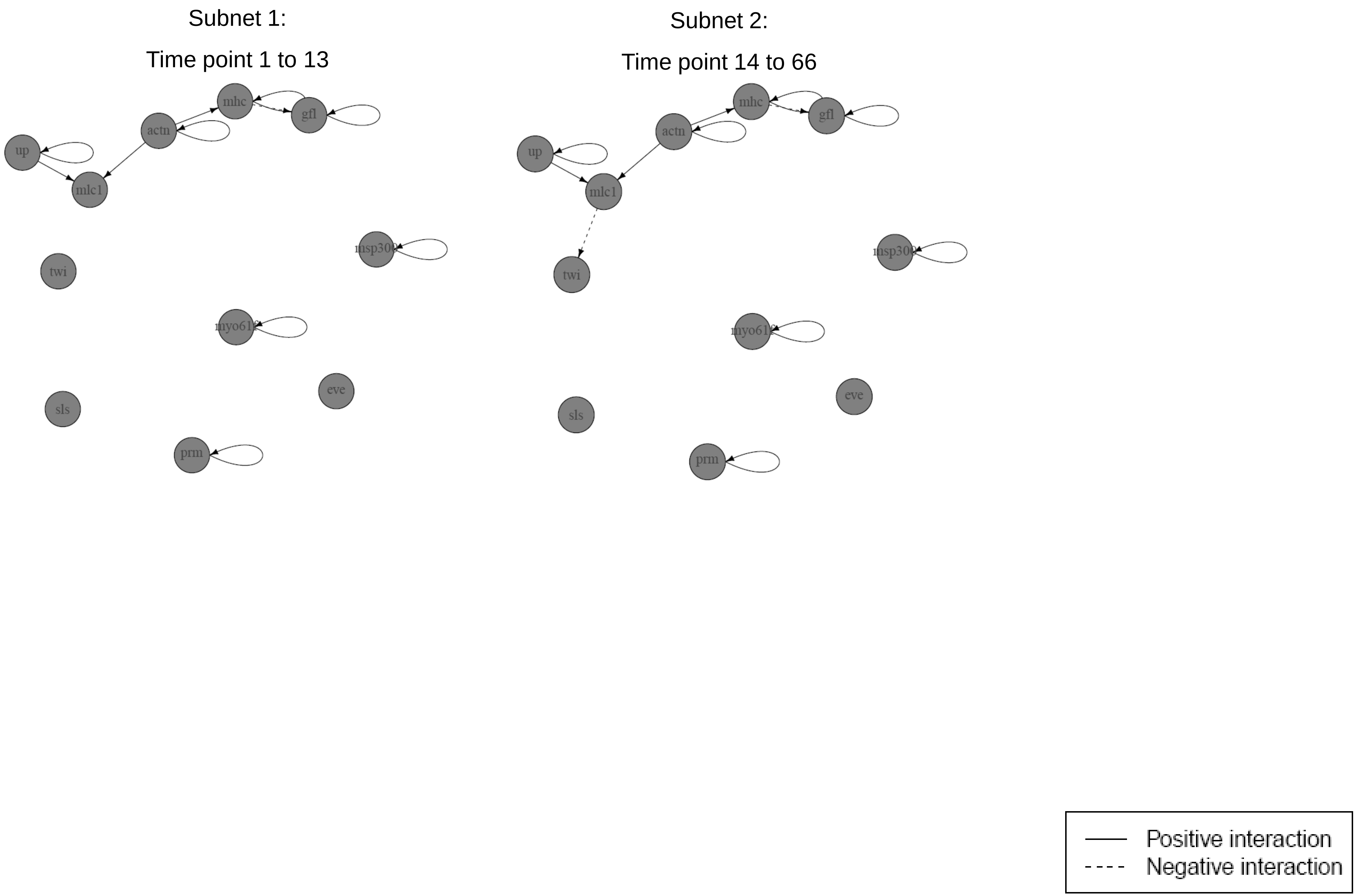

ARTIVA on Drosophila muscle-related continuous gene expression dataset

cCP = 0.4 cEdges = 0.4 threshold=0.5

Subnet 1: Time point 1 to 12

Subnet 2: Time point 13 to 66

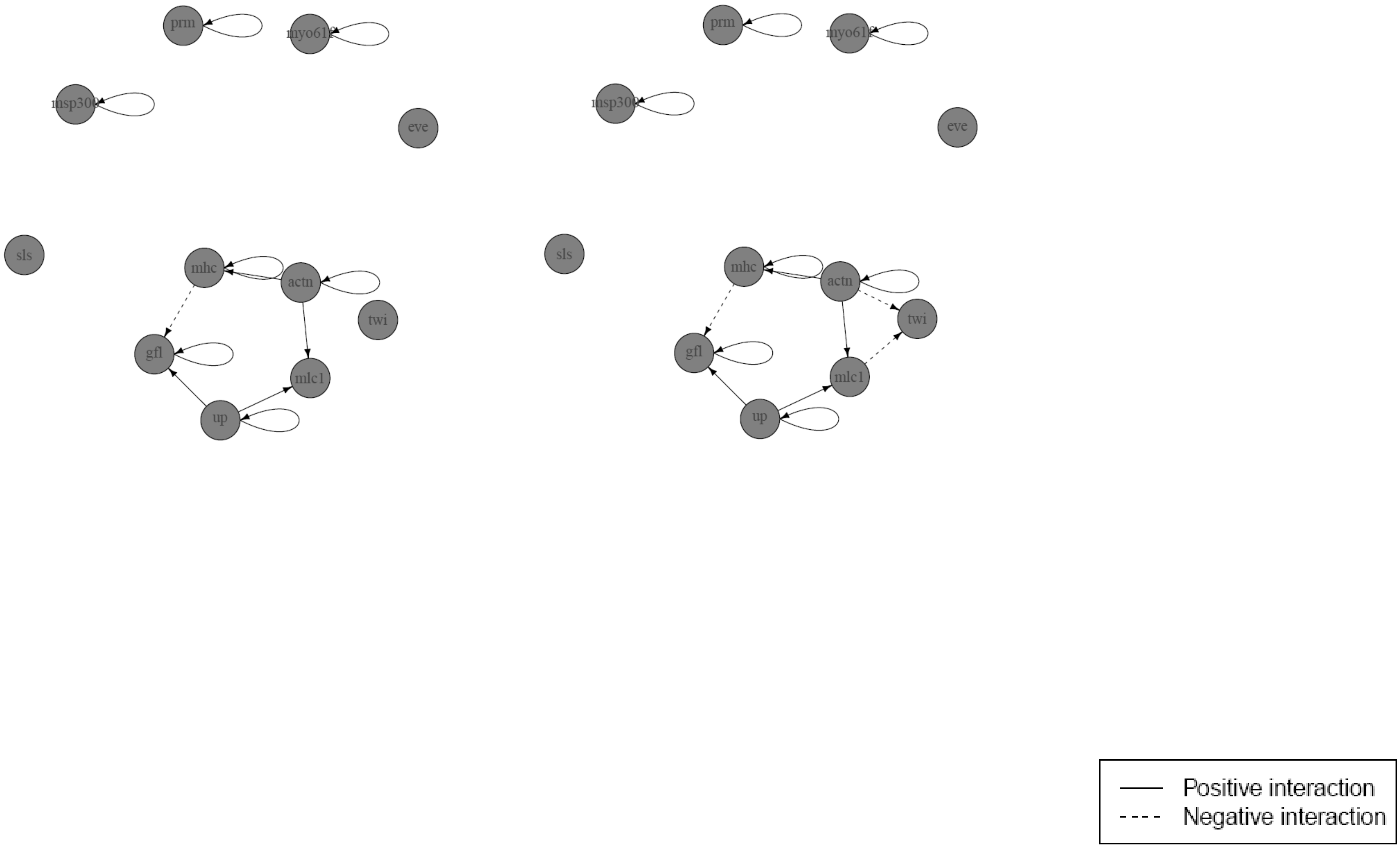

ARTIVA on Drosophila muscle-related continuous gene expression dataset

cCP = 0.5 cEdges = 0.5 threshold=0.5

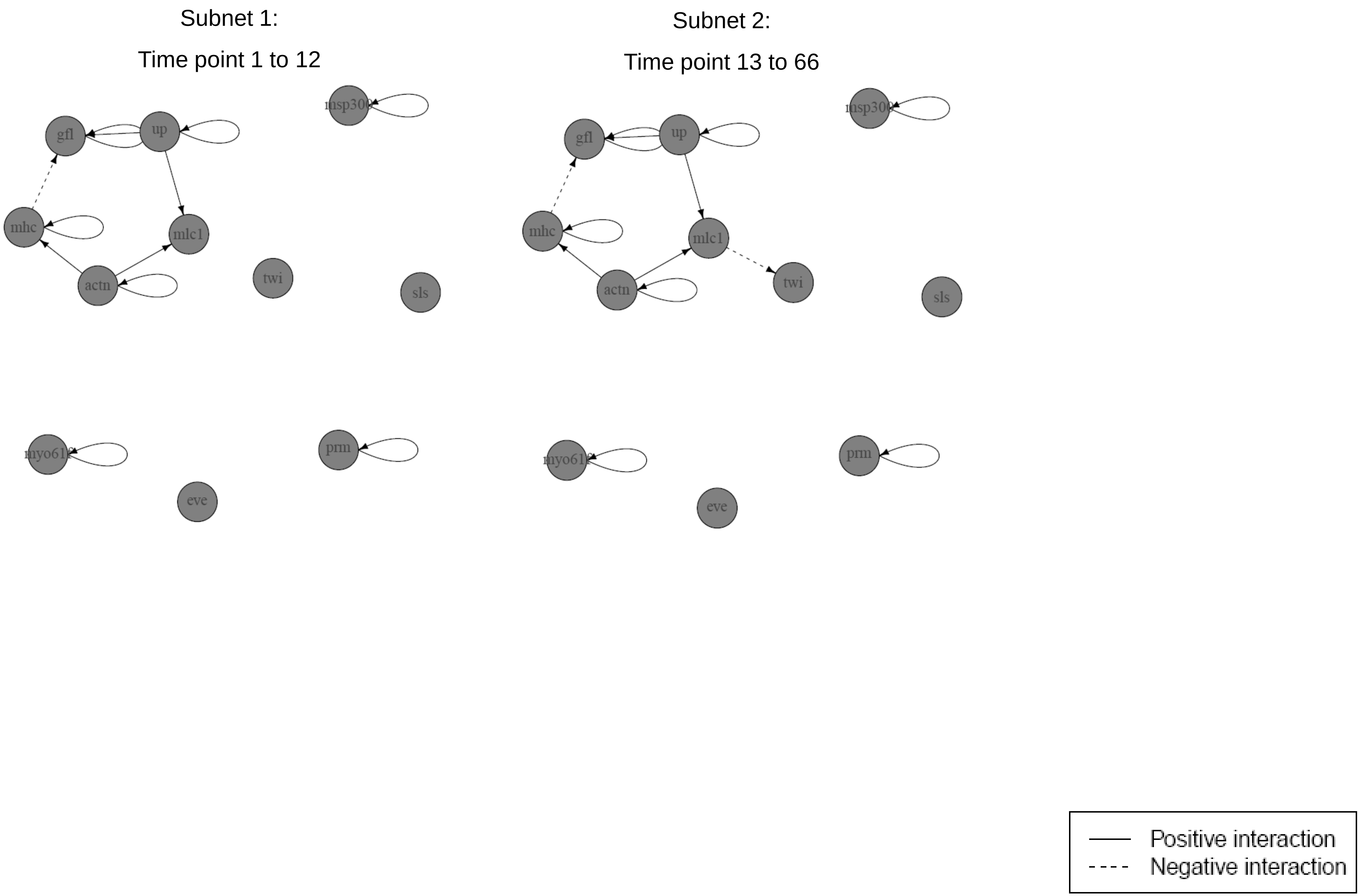

ARTIVA on Drosophila muscle-related continuous gene expression dataset

cCP = 0.6 cEdges = 0.6 threshold=0.5

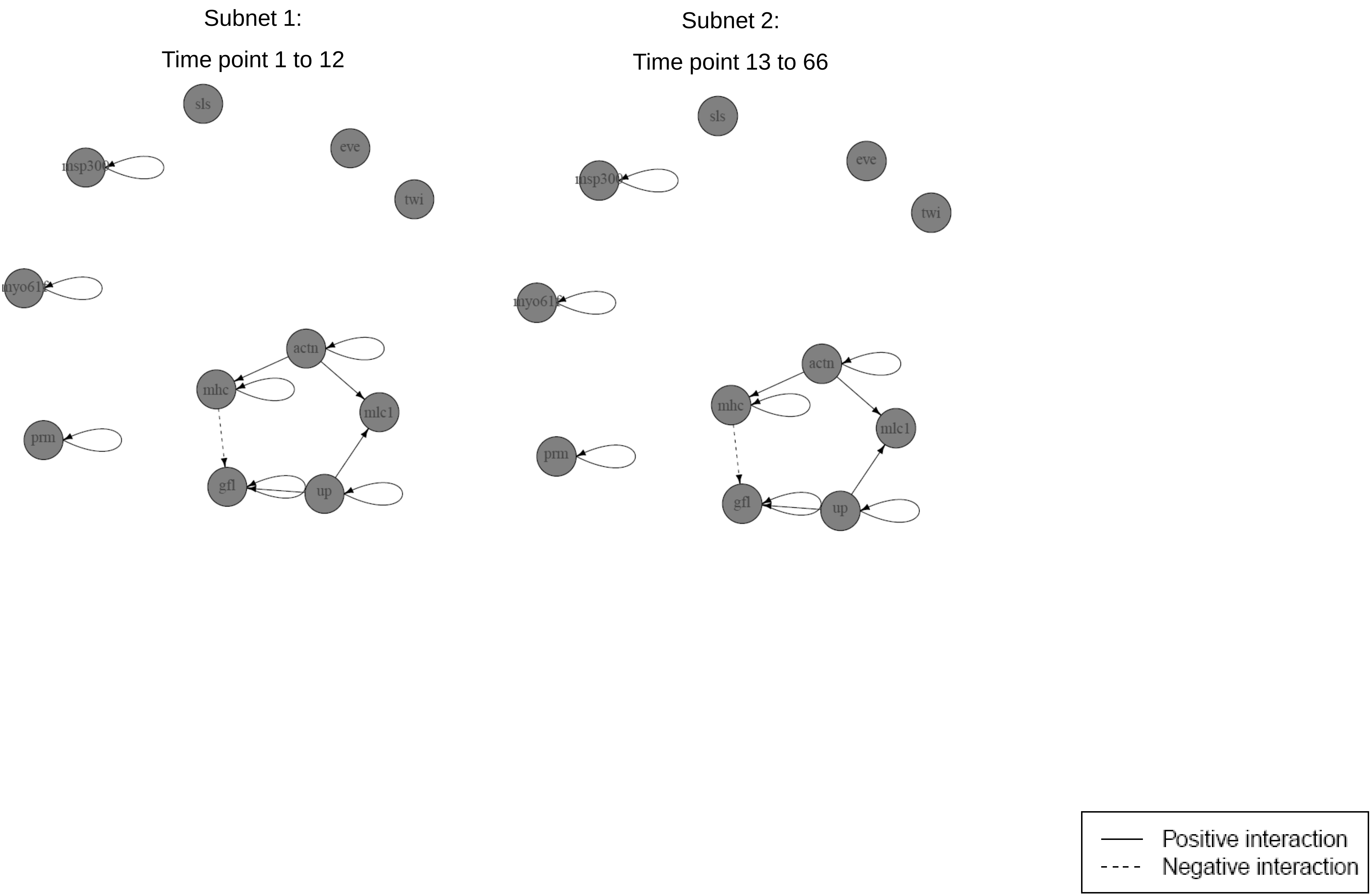

ARTIVA on Drosophila muscle-related continuous gene expression dataset

cCP = 0.7 cEdges = 0.7 threshold=0.5

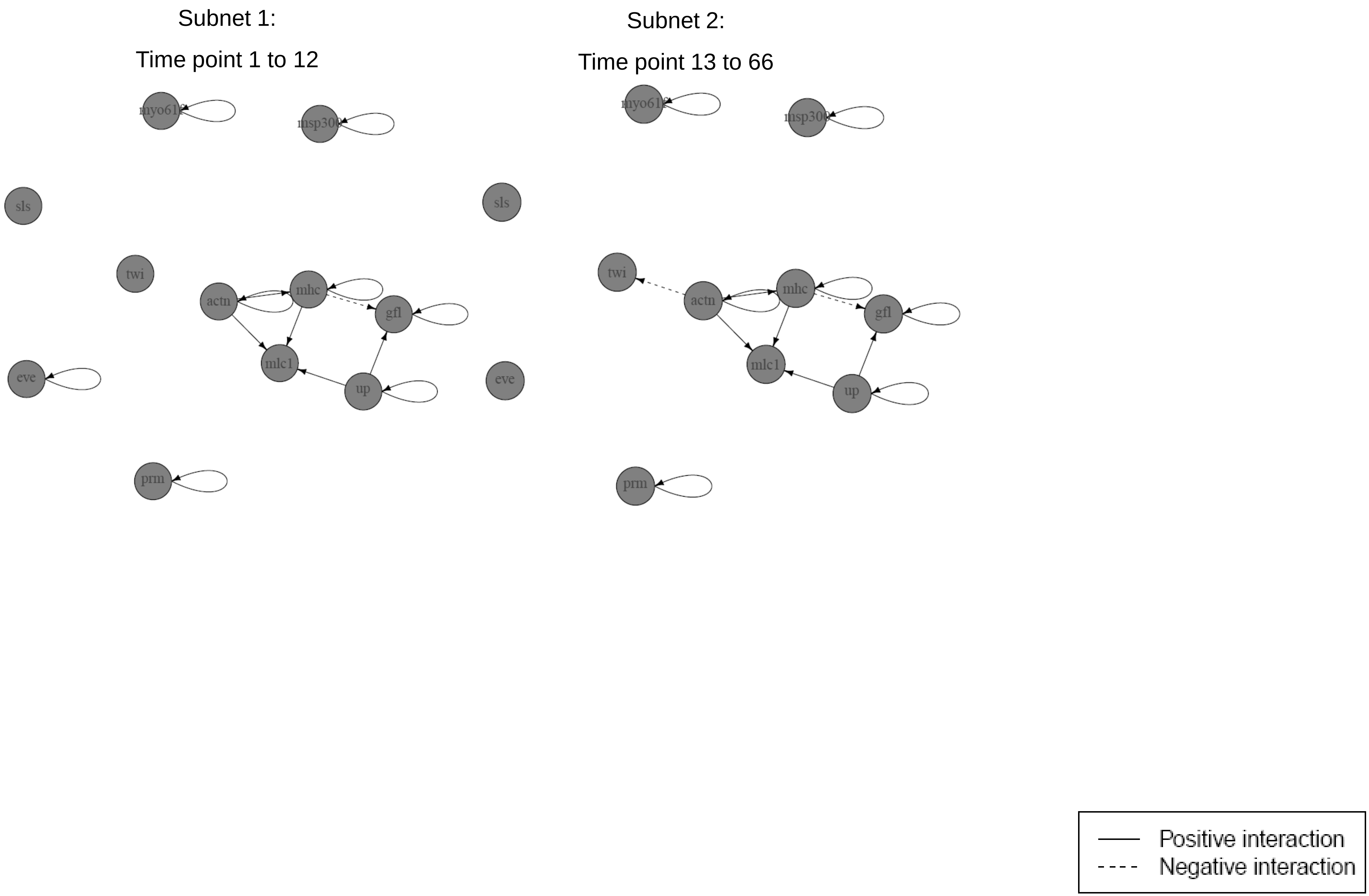

ARTIVA on Drosophila muscle-related continuous gene expression dataset

cCP = 0.8 cEdges = 0.8 threshold=0.5

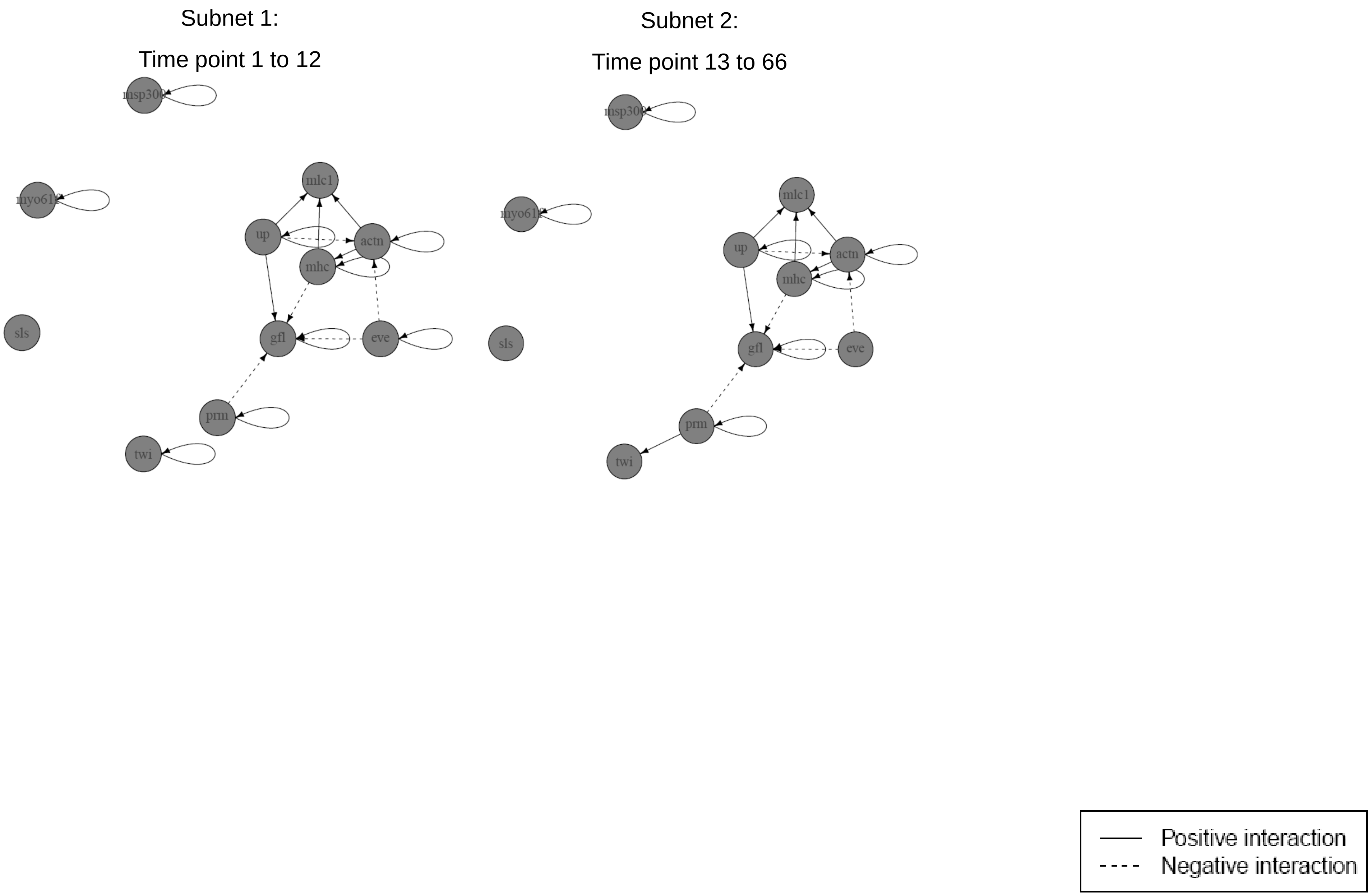

ARTIVA on Drosophila muscle-related continuous gene expression dataset

cCP = 0.9 cEdges = 0.9 threshold=0.5

Subnet 1:

Time point 1 to 12

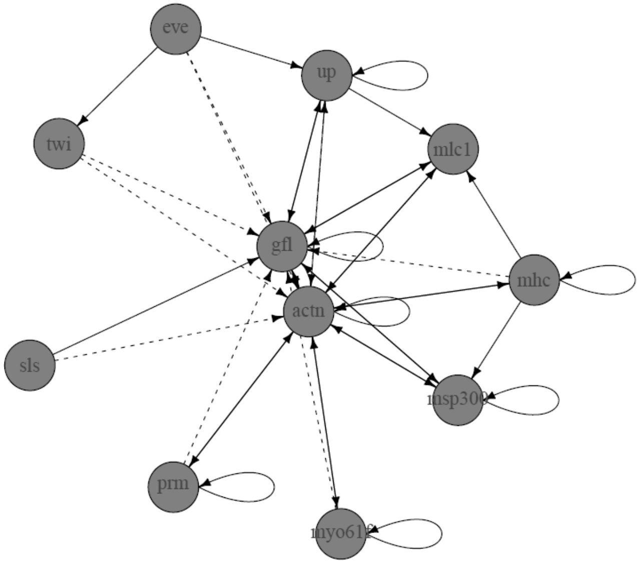

Subnet 2:

Time point 13 to 66

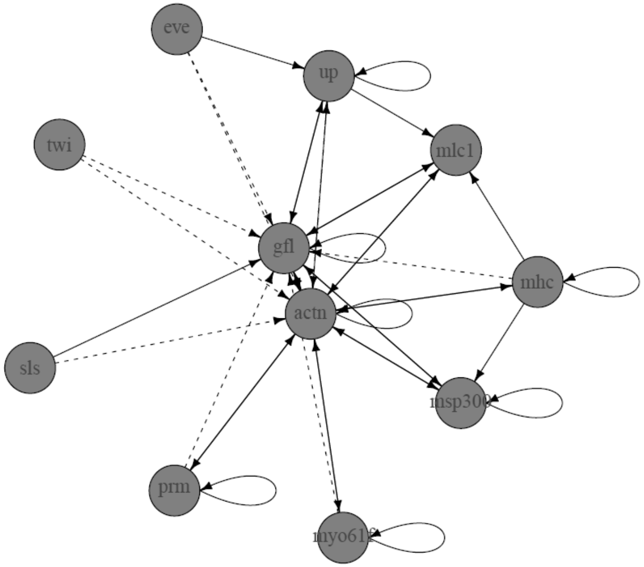

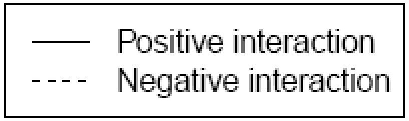

Supplementary File 3. Prediction results of *nhDBN* on the simulated dataset and real biological gene expression dataset under different parameter settings. The connectivity matrix for nodes and time points are the original output by the *nhDBN* package. The grey shading is used to indicate the connectivity probabilities between time points (black = 0 and white = 1). The *nhDBN* method did not recover any transition time for the simulated data. Therefore, the connectivity probability plots for the simulated data are all white, that is, all values in the connectivity matrix are 1, suggesting that there is only one epoch.

nhDBN on Simulation dataset: k = 2; p = 1e-4

Connectivity probabilities between nodes

	1	2	3	4	5	6	7	8	9	10
**1**	0	0	0	0.2	1	0	0	0	0.1	0.05
**2**	0	0	0	0.25	1	0	0	0	0	0.1
**3**	0	0	0	0.25	1	0.05	0	0	0	0.05
**4**	0	0	0	0.3	0	0.15	0	0	0.4	0
**5**	0	0	0	0	0	1	0	0	0.25	0.9
**6**	0	0	0	0.05	0	0.05	0	0	0.15	1
**7**	0	0	0	0.1	0	0	0	0	0.05	0
**8**	0	0	0	0.1	0	0.15	0	0	0.1	0.2
**9**	0	0	0	1	0	1	0	0	0.5	0.1
**10**	0	0	0	0	0	0.05	0	0	1	0

* The color yellow marks the edge whose probability is bigger than 0.5

Connectivity probabilities between time points

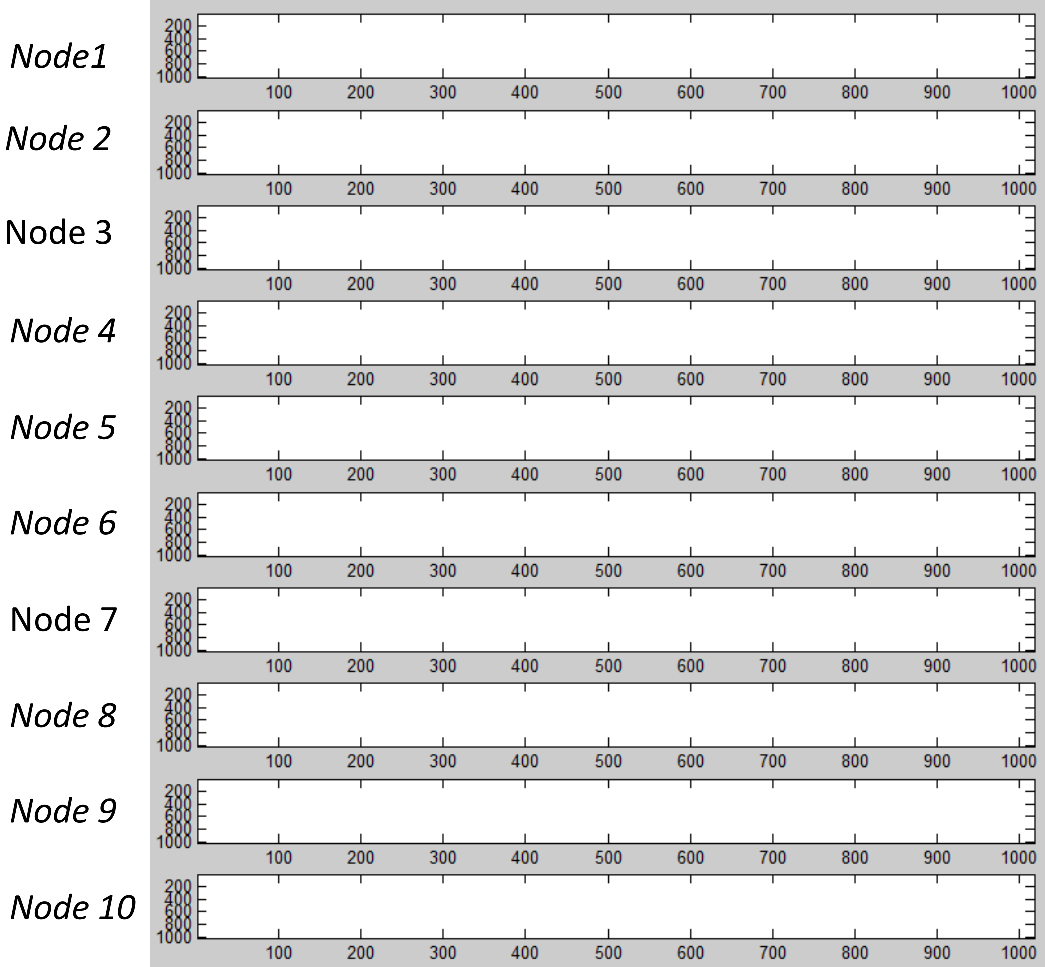

* The grey shading is used to indicate the connectivity probabilities between time points (black=0 and white=1)

# nhDBN on Simulation dataset: k = 2; p = 1e-3

Connectivity probabilities between nodes

	1	2	3	4	5	6	7	8	9	10
**1**	0	0	0	0.05	1	0.05	0	0	0.05	0.05
**2**	0	0	0	0.05	1	0.05	0	0	0	0.15
**3**	0	0	0	0.2	1	0	0	0	0.05	0.1
**4**	0	0	0	0.2	0	0.15	0	0	0.25	0
**5**	0	0	0	0.15	0	1	0	0	0.25	0.85
**6**	0	0	0	0.15	0	0.05	0	0	0.45	1
**7**	0	0	0	0.1	0	0.1	0	0	0	0.1
**8**	0	0	0	0.1	0	0.05	0	0	0	0.2
**9**	0	0	0	1	0	1	0	0	0.5	0.05
**10**	0	0	0	0.15	0	0.1	0	0	1	0

* The color yellow marks the edge whose probability is bigger than 0.5

Connectivity probabilities between time points

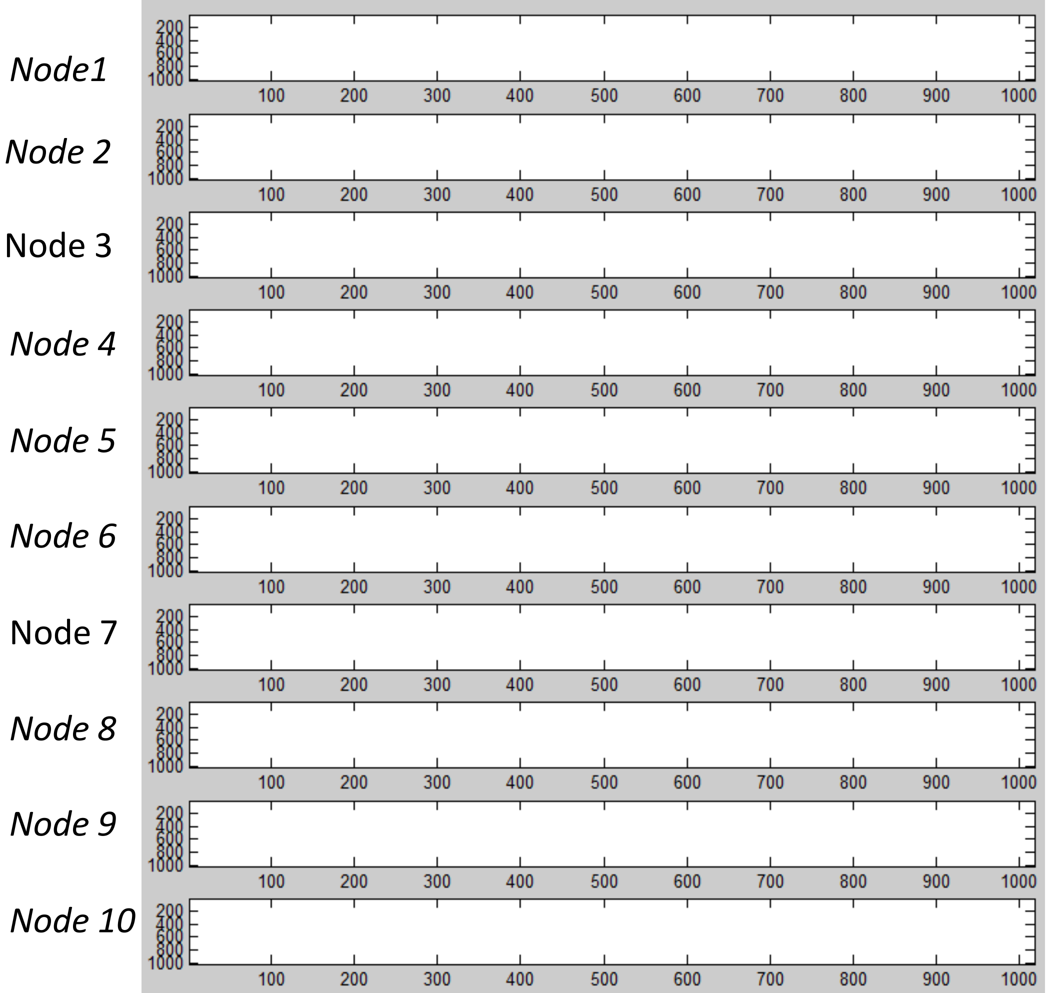

* The grey shading is used to indicate the connectivity probabilities between time points (black=0 and white=1)

# nhDBN on Simulation dataset: k = 2; p = 1e-2

Connectivity probabilities between nodes

	1	2	3	4	5	6	7	8	9	10
**1**	0	0	0	0.05	1	0.05	0	0	0.05	0.05
**2**	0	0	0	0.05	1	0.05	0	0	0	0.15
**3**	0	0	0	0.2	1	0	0	0	0.05	0.1
**4**	0	0	0	0.2	0	0.15	0	0	0.25	0
**5**	0	0	0	0.15	0	1	0	0	0.25	0.85
**6**	0	0	0	0.15	0	0.05	0	0	0.45	1
**7**	0	0	0	0.1	0	0.1	0	0	0	0.1
**8**	0	0	0	0.1	0	0.05	0	0	0	0.2
**9**	0	0	0	1	0	1	0	0	0.5	0.05
**10**	0	0	0	0.15	0	0.1	0	0	1	0

* The color yellow marks the edge whose probability is bigger than 0.5

Connectivity probabilities between time points

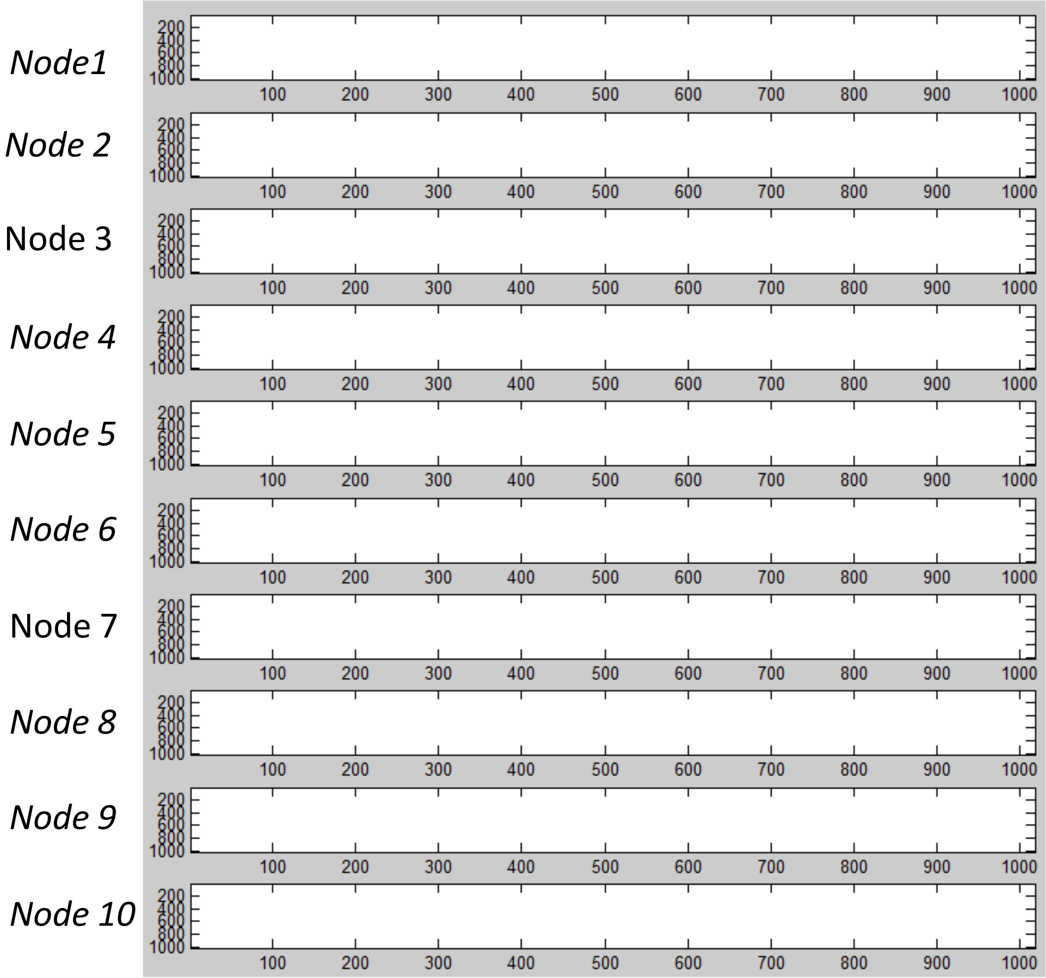

* The grey shading is used to indicate the connectivity probabilities between time points (black=0 and white=1)

# nhDBN on Simulation dataset: k = 2; p = 0.1

Connectivity probabilities between nodes

	1	2	3	4	5	6	7	8	9	10
**1**	0	0	0	0.05	1	0.05	0	0	0.05	0.05
**2**	0	0	0	0.05	1	0.05	0	0	0	0.15
**3**	0	0	0	0.2	1	0	0	0	0.05	0.1
**4**	0	0	0	0.2	0	0.15	0	0	0.25	0
**5**	0	0	0	0.15	0	1	0	0	0.25	0.85
**6**	0	0	0	0.15	0	0.05	0	0	0.45	1
**7**	0	0	0	0.1	0	0.1	0	0	0	0.1
**8**	0	0	0	0.1	0	0.05	0	0	0	0.2
**9**	0	0	0	1	0	1	0	0	0.5	0.05
**10**	0	0	0	0.15	0	0.1	0	0	1	0

* The color yellow marks the edge whose probability is bigger than 0.5

Connectivity probabilities between time points

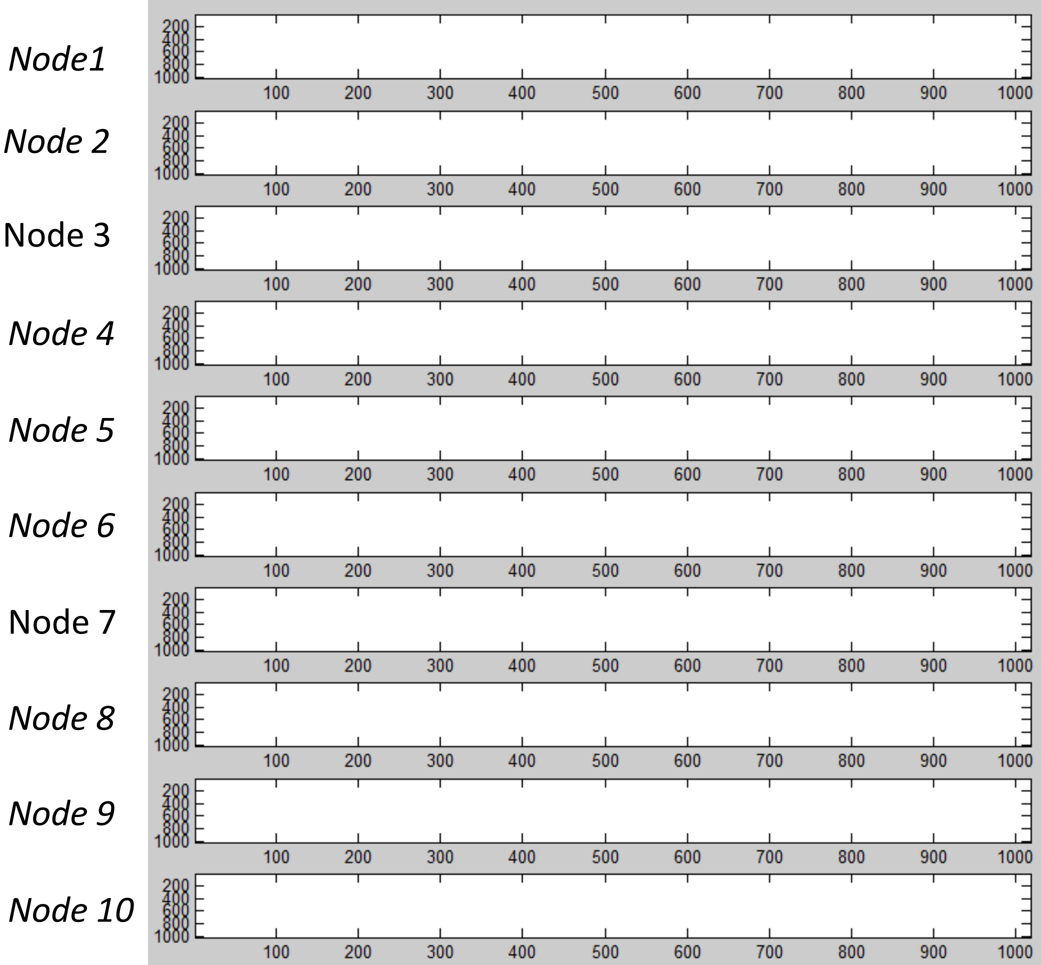

* The grey shading is used to indicate the connectivity probabilities between time points (black=0 and white=1)

# nhDBN on Simulation dataset: k = 2; p = 0.5

Connectivity probabilities between nodes

	1	2	3	4	5	6	7	8	9	10
**1**	0	0	0	0.05	1	0.05	0	0	0.05	0.05
**2**	0	0	0	0.05	1	0.05	0	0	0	0.15
**3**	0	0	0	0.2	1	0	0	0	0.05	0.1
**4**	0	0	0	0.2	0	0.15	0	0	0.25	0
**5**	0	0	0	0.15	0	1	0	0	0.25	0.85
**6**	0	0	0	0.15	0	0.05	0	0	0.45	1
**7**	0	0	0	0.1	0	0.1	0	0	0	0.1
**8**	0	0	0	0.1	0	0.05	0	0	0	0.2
**9**	0	0	0	1	0	1	0	0	0.5	0.05
**10**	0	0	0	0.15	0	0.1	0	0	1	0

* The color yellow marks the edge whose probability is bigger than 0.5

Connectivity probabilities between time points

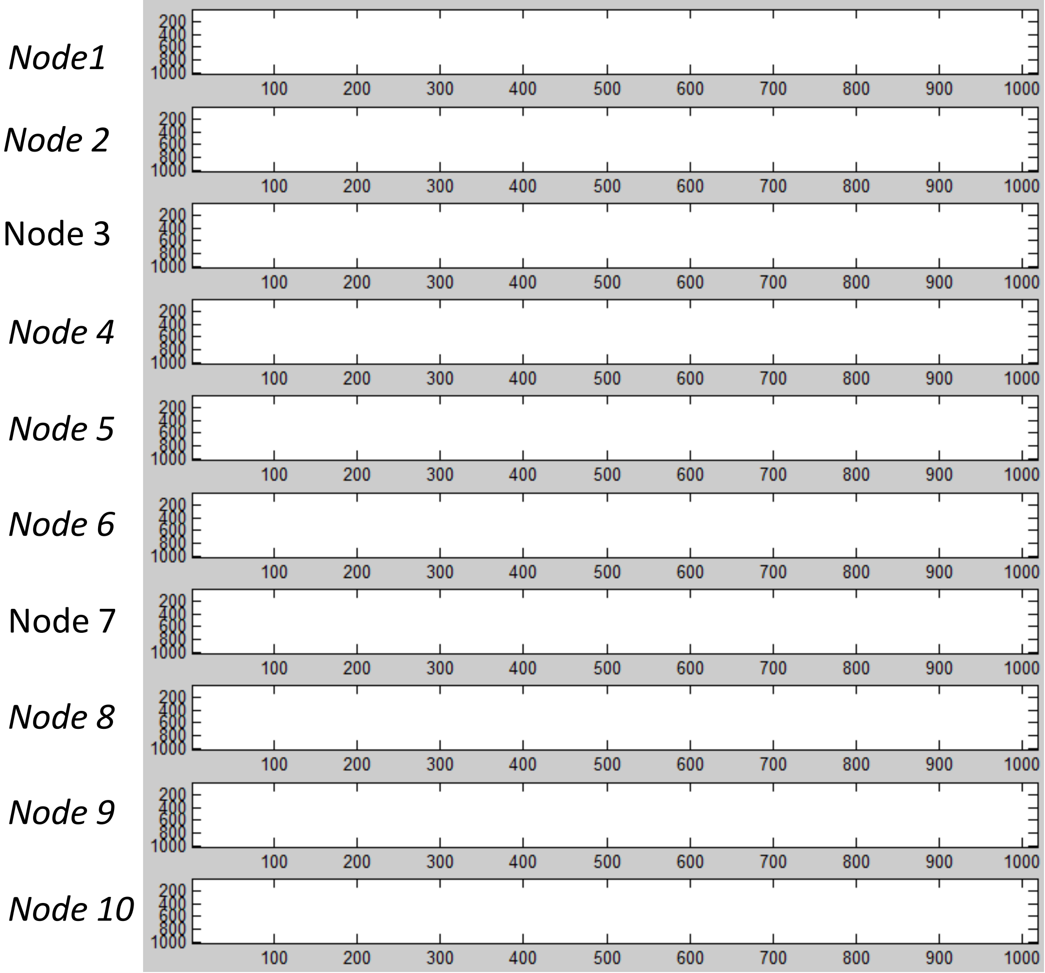

* The grey shading is used to indicate the connectivity probabilities between time points (black=0 and white=1)

# nhDBN on Simulation dataset: k = 2; p = 1

Connectivity probabilities between nodes

	1	2	3	4	5	6	7	8	9	10
**1**	0	0	0	0.05	1	0.05	0	0	0.05	0.05
**2**	0	0	0	0.05	1	0.05	0	0	0	0.15
**3**	0	0	0	0.2	1	0	0	0	0.05	0.1
**4**	0	0	0	0.2	0	0.15	0	0	0.25	0
**5**	0	0	0	0.15	0	1	0	0	0.25	0.85
**6**	0	0	0	0.15	0	0.05	0	0	0.45	1
**7**	0	0	0	0.1	0	0.1	0	0	0	0.1
**8**	0	0	0	0.1	0	0.05	0	0	0	0.2
**9**	0	0	0	1	0	1	0	0	0.5	0.05
**10**	0	0	0	0.15	0	0.1	0	0	1	0

* The color yellow marks the edge whose probability is bigger than 0.5

Connectivity probabilities between time points

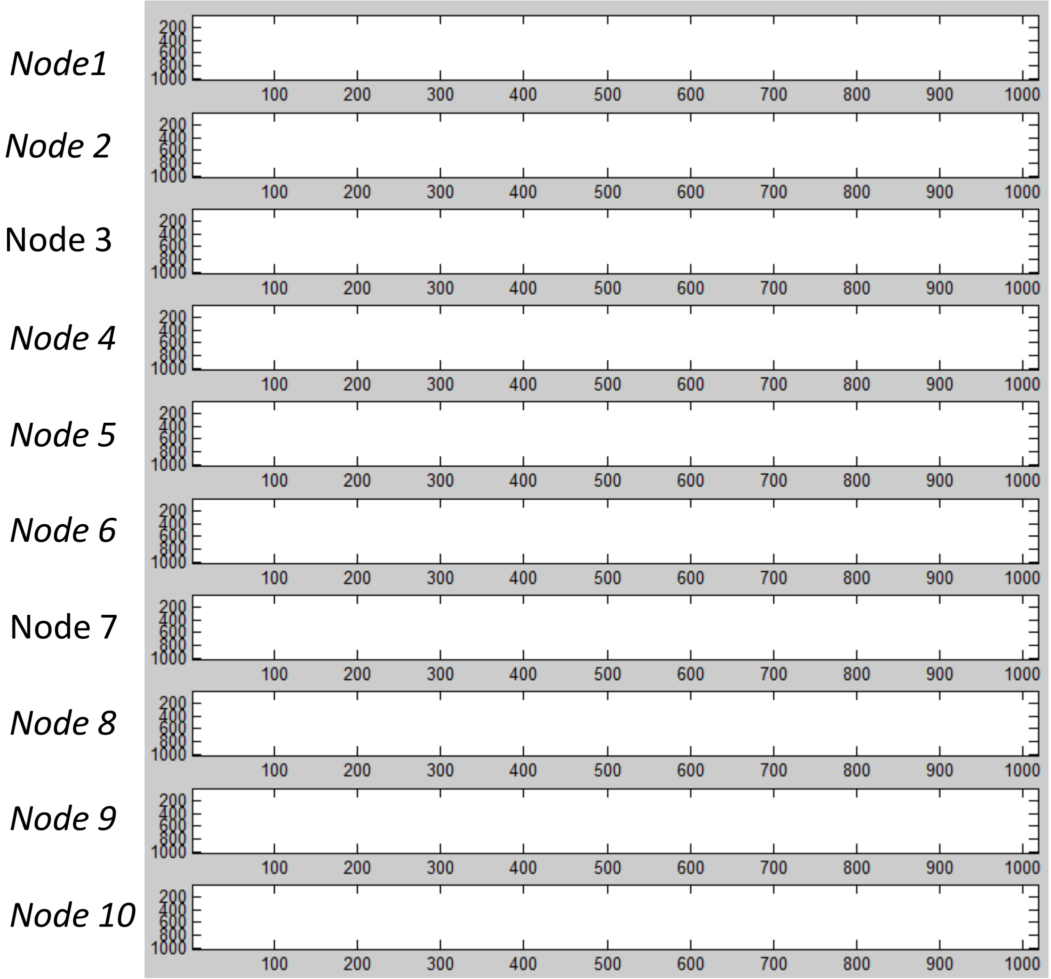

* The grey shading is used to indicate the connectivity probabilities between time points (black=0 and white=1)

# nhDBN on Simulation dataset: k = 10; p = 1

Connectivity probabilities between nodes

	1	2	3	4	5	6	7	8	9	10
**1**	0	0	0	0.05	1	0.05	0	0	0.05	0.05
**2**	0	0	0	0.05	1	0.05	0	0	0	0.15
**3**	0	0	0	0.2	1	0	0	0	0.05	0.1
**4**	0	0	0	0.2	0	0.15	0	0	0.25	0
**5**	0	0	0	0.15	0	1	0	0	0.25	0.85
**6**	0	0	0	0.15	0	0.05	0	0	0.45	1
**7**	0	0	0	0.1	0	0.1	0	0	0	0.1
**8**	0	0	0	0.1	0	0.05	0	0	0	0.2
**9**	0	0	0	1	0	1	0	0	0.5	0.05
**10**	0	0	0	0.15	0	0.1	0	0	1	0

* The color yellow marks the edge whose probability is bigger than 0.5

Connectivity probabilities between time points

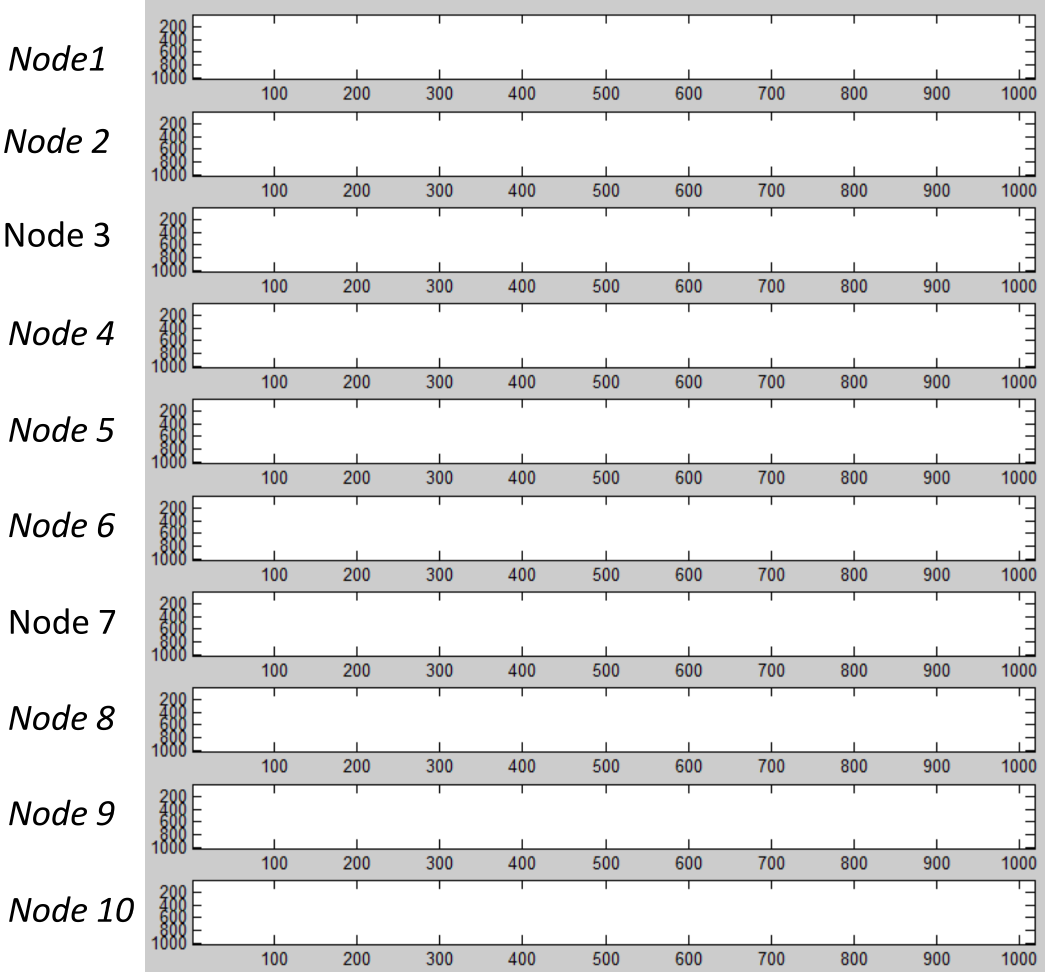

* The grey shading is used to indicate the connectivity probabilities between time points (black=0 and white=1)

# nhDBN on Simulation dataset: k = 50; p = 1

Connectivity probabilities between nodes

	1	2	3	4	5	6	7	8	9	10
**1**	0	0	0	0.05	1	0.05	0	0	0.05	0.05
**2**	0	0	0	0.05	1	0.05	0	0	0	0.15
**3**	0	0	0	0.2	1	0	0	0	0.05	0.1
**4**	0	0	0	0.2	0	0.15	0	0	0.25	0
**5**	0	0	0	0.15	0	1	0	0	0.25	0.85
**6**	0	0	0	0.15	0	0.05	0	0	0.45	1
**7**	0	0	0	0.1	0	0.1	0	0	0	0.1
**8**	0	0	0	0.1	0	0.05	0	0	0	0.2
**9**	0	0	0	1	0	1	0	0	0.5	0.05
**10**	0	0	0	0.15	0	0.1	0	0	1	0

* The color yellow marks the edge whose probability is bigger than 0.5

Connectivity probabilities between time points

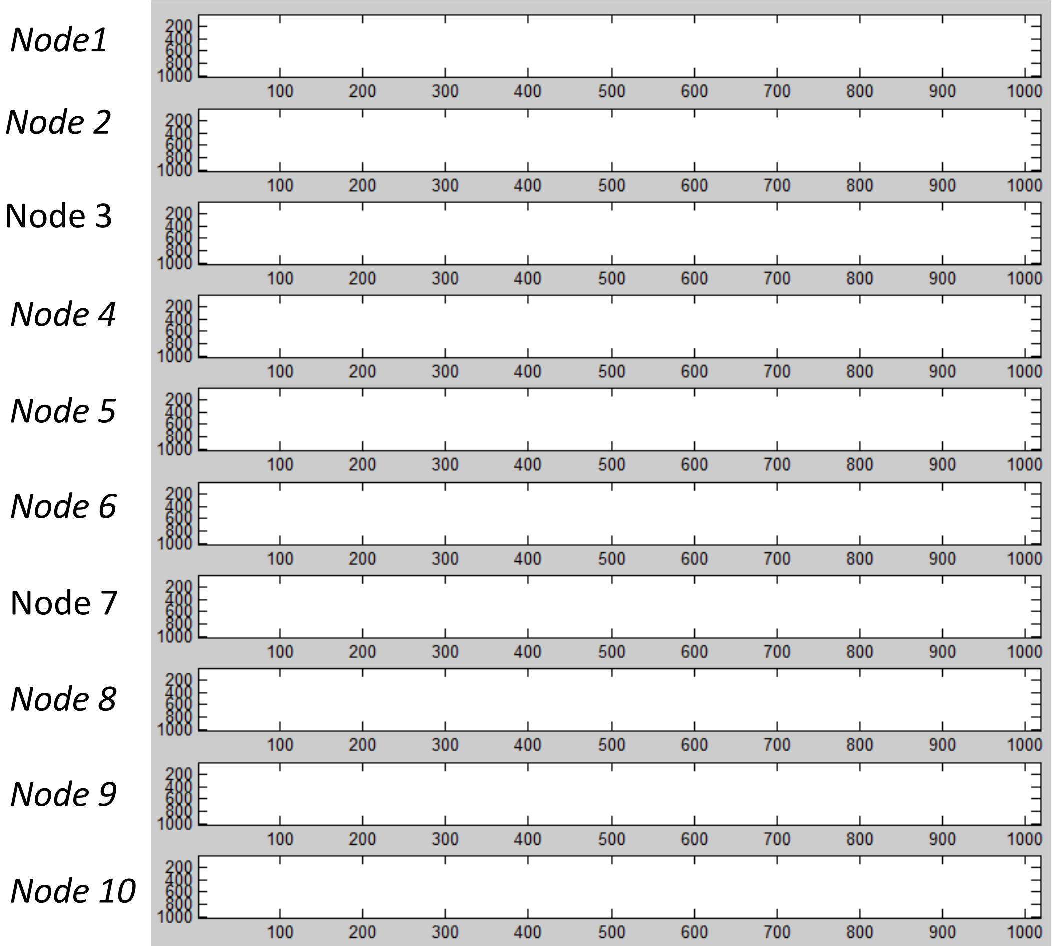

* The grey shading is used to indicate the connectivity probabilities between time points (black=0 and white=1)

# nhDBN on Simulation dataset: k = 100; p = 1

Connectivity probabilities between nodes

	1	2	3	4	5	6	7	8	9	10
**1**	0	0	0	0.05	1	0.05	0	0	0.05	0.05
**2**	0	0	0	0.05	1	0.05	0	0	0	0.15
**3**	0	0	0	0.2	1	0	0	0	0.05	0.1
**4**	0	0	0	0.2	0	0.15	0	0	0.25	0
**5**	0	0	0	0.15	0	1	0	0	0.25	0.85
**6**	0	0	0	0.15	0	0.05	0	0	0.45	1
**7**	0	0	0	0.1	0	0.1	0	0	0	0.1
**8**	0	0	0	0.1	0	0.05	0	0	0	0.2
**9**	0	0	0	1	0	1	0	0	0.5	0.05
**10**	0	0	0	0.15	0	0.1	0	0	1	0

* The color yellow marks the edge whose probability is bigger than 0.5

Connectivity probabilities between time points

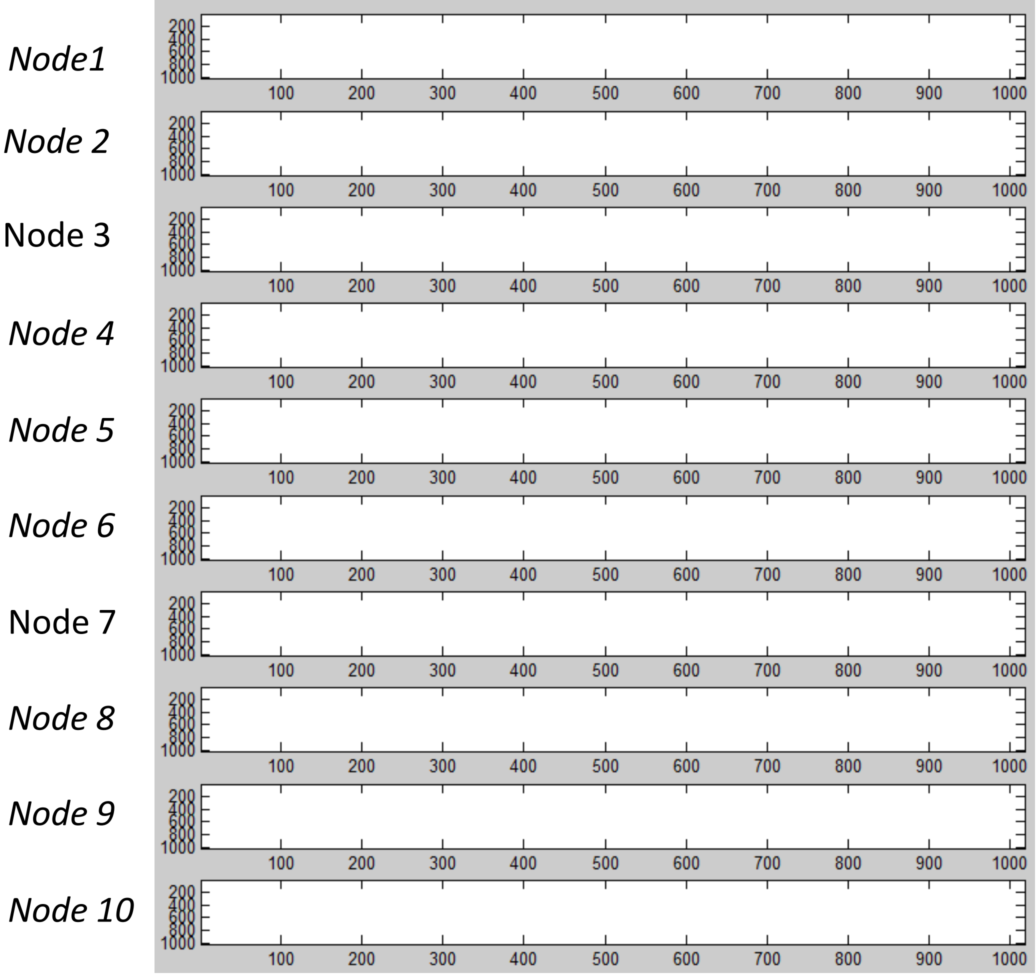

* The grey shading is used to indicate the connectivity probabilities between time points (black=0 and white=1)

# nhDBN on Simulation dataset: k = 100; p = 1e-5

Connectivity probabilities between nodes

	1	2	3	4	5	6	7	8	9	10
**1**	0	0	0	0.05	1	0.05	0	0	0.05	0.05
**2**	0	0	0	0.05	1	0.05	0	0	0	0.15
**3**	0	0	0	0.2	1	0	0	0	0.05	0.1
**4**	0	0	0	0.2	0	0.15	0	0	0.25	0
**5**	0	0	0	0.15	0	1	0	0	0.25	0.85
**6**	0	0	0	0.15	0	0.05	0	0	0.45	1
**7**	0	0	0	0.1	0	0.1	0	0	0	0.1
**8**	0	0	0	0.1	0	0.05	0	0	0	0.2
**9**	0	0	0	1	0	1	0	0	0.5	0.05
**10**	0	0	0	0.15	0	0.1	0	0	1	0

* The color yellow marks the edge whose probability is bigger than 0.5

Connectivity probabilities between time points

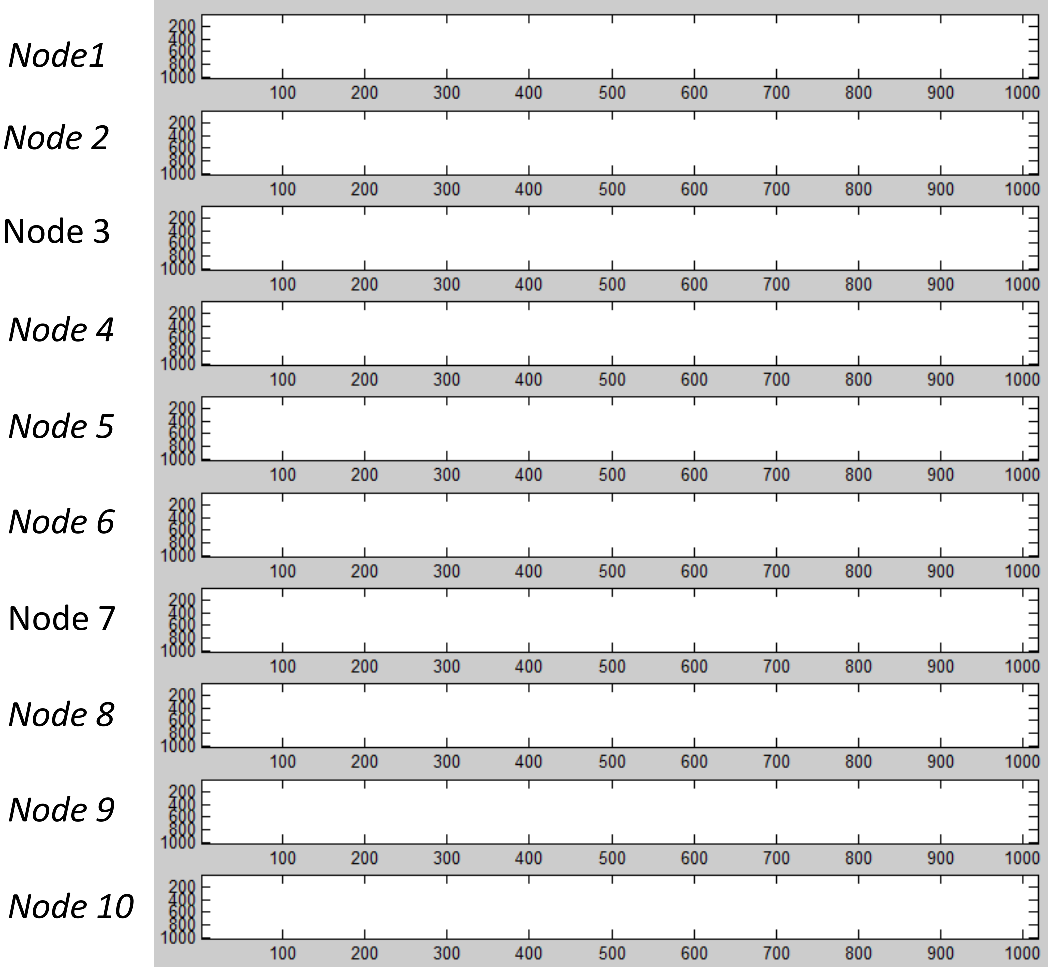

* The grey shading is used to indicate the connectivity probabilities between time points (black=0 and white=1)

# nhDBN on Drosophila muscle-related discrete gene expression dataset: k = 2; p = 1e-6

Connectivity probabilities between genes

	eve	gfl	twi	mlc1	sls	mhc	prm	actn	up	myo61f	msp300
eve	0.9	0.1	0.1	0.2	0.1	0.05	0.2	0.15	0	0	0.25
gfl/lmd	0.1	1	0.15	0.7	0.05	0.05	0	0.05	0	0	0.05
twi	0.25	0.2	1	0	0.75	0.1	0.25	0.7	0.2	0.05	0.25
mlc1	0.2	0.25	0.35	0.4	0.1	0.05	0.05	0.15	1	0.15	0.05
sls	0.15	0	0.15	0	1	0.05	0.05	0.15	0.45	0.35	0
mhc	0.2	0.05	0.1	0.45	0.35	0.2	0.05	0.1	0.1	0.05	0.1
prm	0.2	0.55	0.15	0.05	0	0.05	0	0.05	0.05	1	0
actn	0.05	0.4	0	0.05	0.05	0.2	0.05	1	0.05	0.1	0.1
up	0.15	0	0.45	0.1	0.35	0.6	0.3	0.1	0	0.05	0.95
myo61f	0.1	0.05	0.2	0	0.1	0.6	1	0.4	0.15	1	0.2
msp300	0.15	0.15	0.1	1	0.15	1	1	0.05	1	0.1	1

* The color yellow marks the edge whose probability is bigger than 0.5

Connectivity probabilities between time points

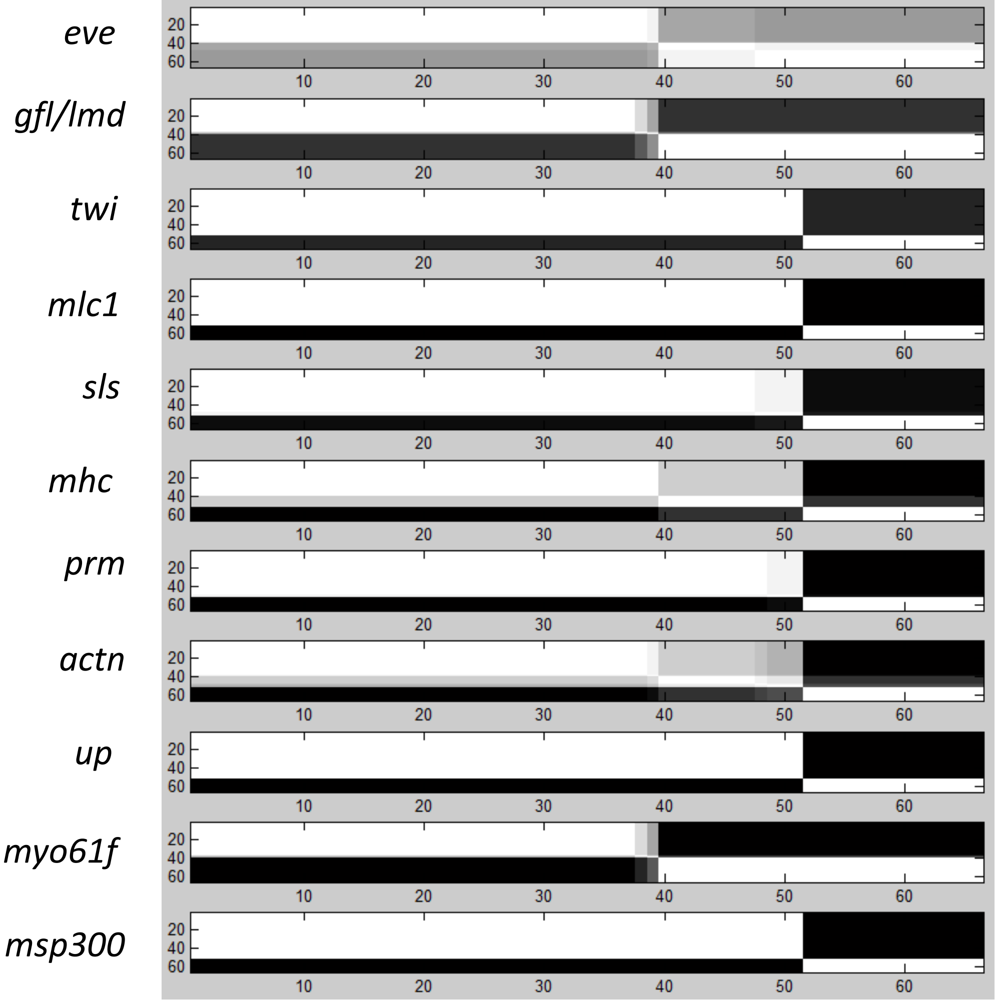

* The grey shading is used to indicate the connectivity probabilities between time points (black=0 and white=1)

# nhDBN on Drosophila muscle-related discrete gene expression dataset: k = 2; p = 1e-5

Connectivity probabilities between genes

	eve	gfl	twi	mlc1	sls	mhc	prm	actn	up	myo61f	msp300
eve	0.45	0.2	0.15	0.1	0	0	0.2	0.1	0.1	0	0.15
gfl/lmd	0.3	1	0.2	0.75	0	0	0.05	0	0.05	0.1	0.05
twi	0.9	0.05	1	0.05	0.85	0	0.45	0.7	0.2	0	0.2
mlc1	0.2	0.1	0.2	0.45	0.05	0	0	0.2	0.95	0.1	0
sls	0.25	0.1	0.1	0	0.95	0.05	0	0	0.15	0.35	0
mhc	0.15	0.1	0.25	0.55	0.25	0	0.05	0.15	0.25	0.1	0.05
prm	0.1	0.15	0.35	0.05	0.05	0.05	0	0.15	0.2	1	0.05
actn	0.1	0.35	0.2	0	0.1	1	0.05	1	0	0.1	0.25
up	0.25	0.25	0.15	0	0.3	0.6	0.2	0	0	0.05	0.95
myo61f	0.1	0	0.2	0	0.2	0.3	0.95	0.3	0.1	1	0.25
msp300	0.1	0.3	0.05	1	0.2	1	1	0.2	1	0.05	1

* The color yellow marks the edge whose probability is bigger than 0.5

Connectivity probabilities between time points

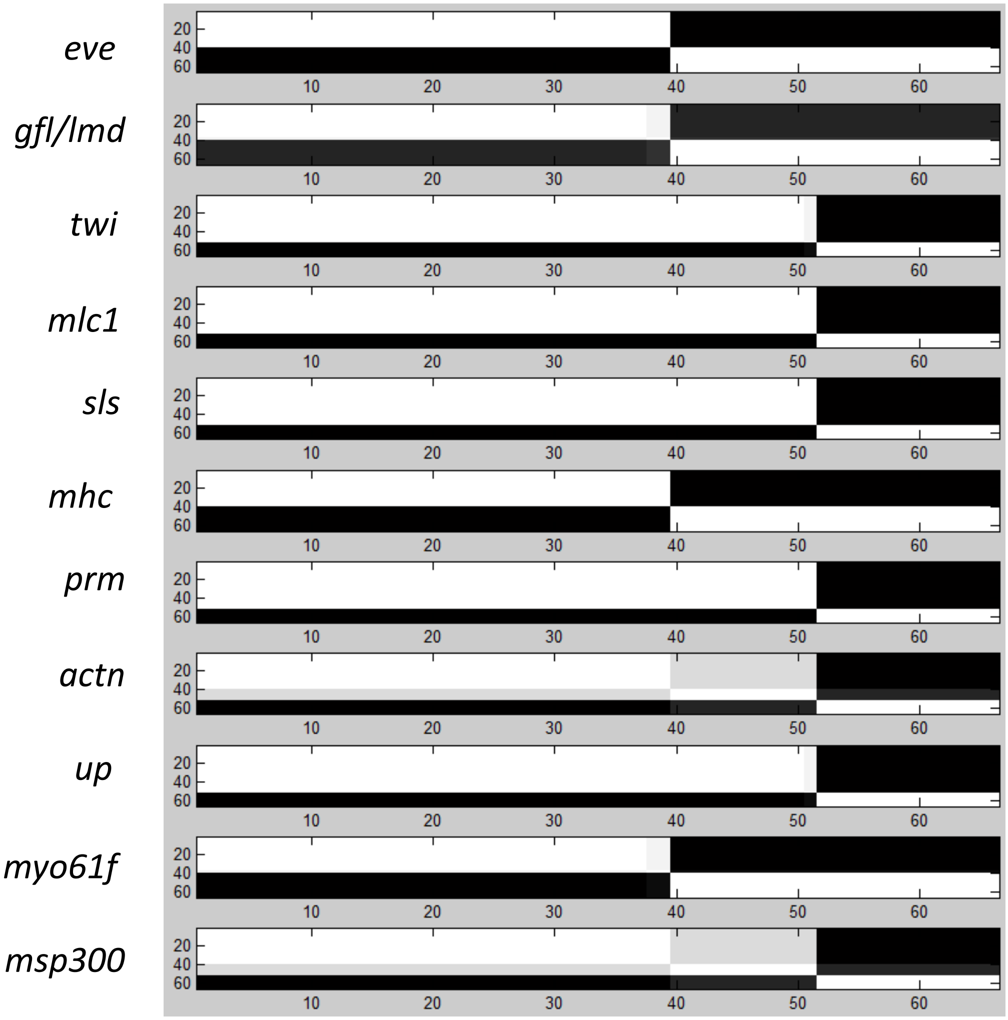

* The grey shading is used to indicate the connectivity probabilities between time points (black=0 and white=1)

# nhDBN on Drosophila muscle-related discrete gene expression dataset: k = 2; p = 1e-4

Connectivity probabilities between genes

	eve	gfl	twi	mlc1	sls	mhc	prm	actn	up	myo61f	msp300
eve	0.75	0.15	0.05	0.15	0.05	0.25	0.3	0.25	0	0.1	0.25
gfl/lmd	0.2	1	0.15	0.85	0	0	0	0	0.05	0.2	0.1
twi	0.55	0.15	1	0	0.9	0.25	0.25	0.75	0.05	0.2	0.05
mlc1	0.15	0.05	0.4	0.2	0.1	0.05	0.15	0.2	1	0.15	0
sls	0.25	0	0	0	1	0.1	0	0	0.1	0.2	0
mhc	0.2	0.1	0.25	0.5	0.2	0.1	0	0.15	0.2	0.05	0
prm	0.1	0.25	0.1	0.05	0.05	0.15	0	0.15	0.25	0.95	0
actn	0.2	0.35	0.25	0.05	0	0.1	0.05	1	0.05	0	0.4
up	0.1	0.1	0.25	0.1	0.4	0.35	0.3	0.15	0.1	0	1
myo61f	0.05	0.05	0.3	0.05	0.1	0.65	0.95	0.2	0.1	1	0.15
msp300	0.2	0.1	0.05	1	0.2	1	1	0.15	1	0	1

* The color yellow marks the edge whose probability is bigger than 0.5

Connectivity probabilities between time points

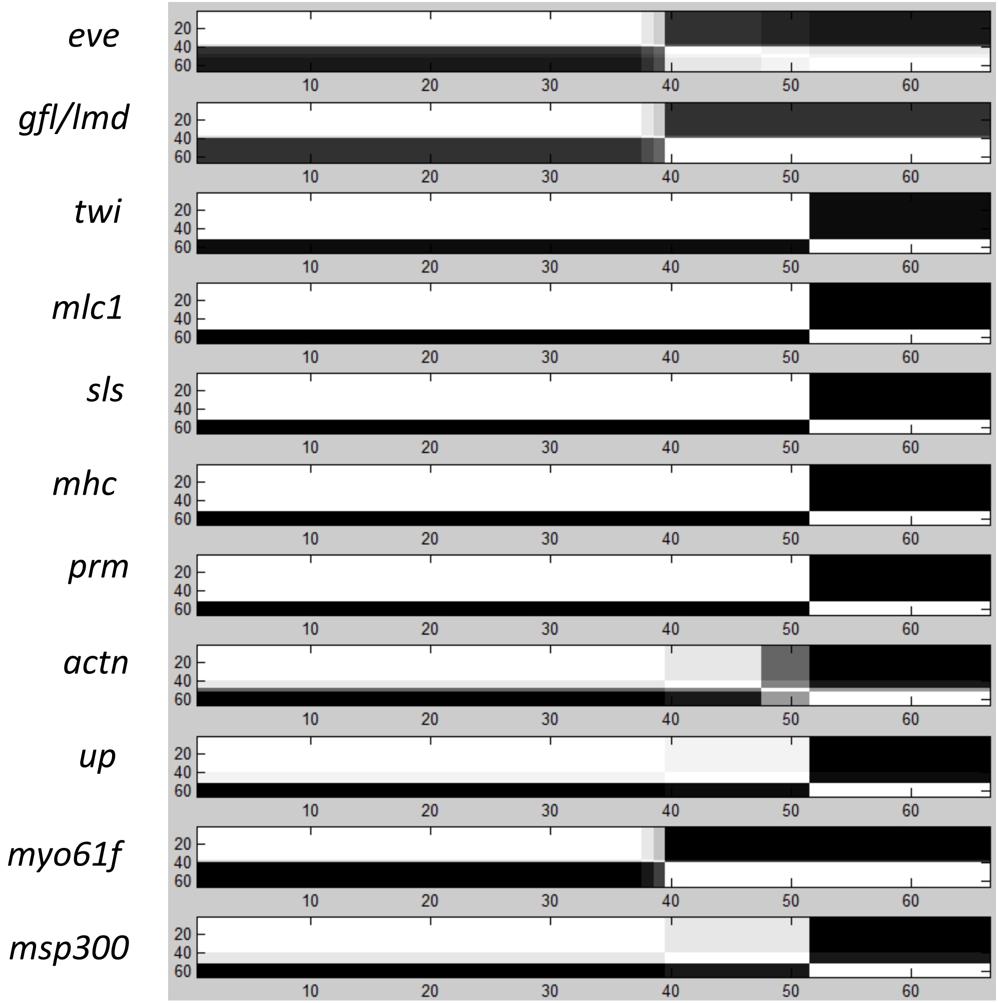

* The grey shading is used to indicate the connectivity probabilities between time points (black=0 and white=1)

# nhDBN on Drosophila muscle-related discrete gene expression dataset: k = 2; p = 1e-3

Connectivity probabilities between genes

	eve	gfl	twi	mlc1	sls	mhc	prm	actn	up	myo61f	msp300
eve	0.55	0.15	0.1	0	0.05	0	0.1	0.1	0	0	0.25
gfl/lmd	0.15	1	0.15	0.75	0	0	0	0.05	0	0.05	0.05
twi	0.55	0.3	1	0	0.95	0	0.3	0.65	0.25	0.25	0.45
mlc1	0.45	0.1	0.25	0.45	0	0	0.05	0.1	1	0.1	0
sls	0.15	0.1	0.1	0.05	1	0	0	0.1	0.25	0.15	0
mhc	0.1	0.15	0.05	0.4	0.35	0.1	0.05	0.2	0.15	0	0
prm	0.05	0.2	0.2	0.1	0.1	0	0.05	0.2	0.1	1	0
actn	0.05	0.3	0.25	0.15	0	1	0.2	1	0.05	0	0.4
up	0.15	0.05	0.35	0.05	0.2	0.5	0.25	0.25	0.1	0	0.9
myo61f	0.25	0.15	0.35	0.05	0.15	0.35	0.9	0.15	0.05	1	0.05
msp300	0.3	0.3	0.05	1	0.15	1	1	0.15	1	0.3	0.9

* The color yellow marks the edge whose probability is bigger than 0.5

Connectivity probabilities between time points

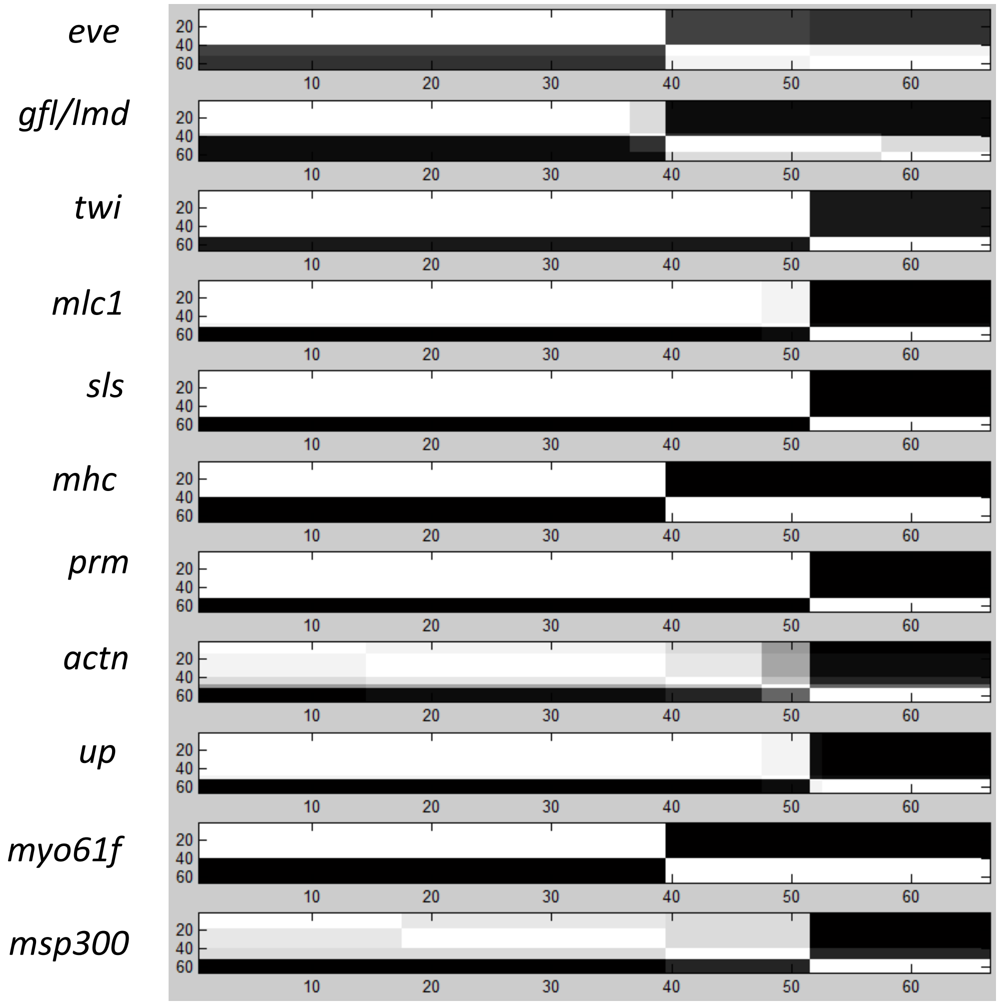

* The grey shading is used to indicate the connectivity probabilities between time points (black=0 and white=1)

# nhDBN on Drosophila muscle-related discrete gene expression dataset: k = 2; p = 1e-2

Connectivity probabilities between genes

	eve	gfl	twi	mlc1	sls	mhc	prm	actn	up	myo61f	msp300
eve	0.6	0	0.1	0.2	0.05	0.15	0.15	0.15	0.05	0.1	0
gfl/lmd	0.15	0.85	0.25	0.5	0.05	0.05	0.1	0.5	0.05	0.1	0.05
twi	0.8	0.7	1	0.05	0.8	0.55	0.7	0.5	0.25	0.1	0.85
mlc1	0.35	0.2	0.1	0.45	0.15	0	0.05	0.1	1	0.1	0.05
sls	0.2	0.1	0.2	0.05	1	0.05	0	0.05	0.1	0.25	0.05
mhc	0.1	0.05	0.1	0.5	0.1	0.1	0	0.15	0.15	0.2	0.1
prm	0.1	0.25	0.2	0.05	0	0	0	0.1	0	0.85	0.1
actn	0.05	0.45	0.65	0.05	0.1	0.1	0	1	0	0.05	0.05
up	0.2	0.2	0.1	0.05	0.3	0.55	0.15	0.05	0.35	0.15	0.9
myo61f	0.2	0.05	0.25	0.1	0.3	0.45	0.85	0.25	0	1	0.05
msp300	0.1	0.1	0.05	1	0.15	1	1	0.15	1	0	0.8

* The color yellow marks the edge whose probability is bigger than 0.5

Connectivity probabilities between time points

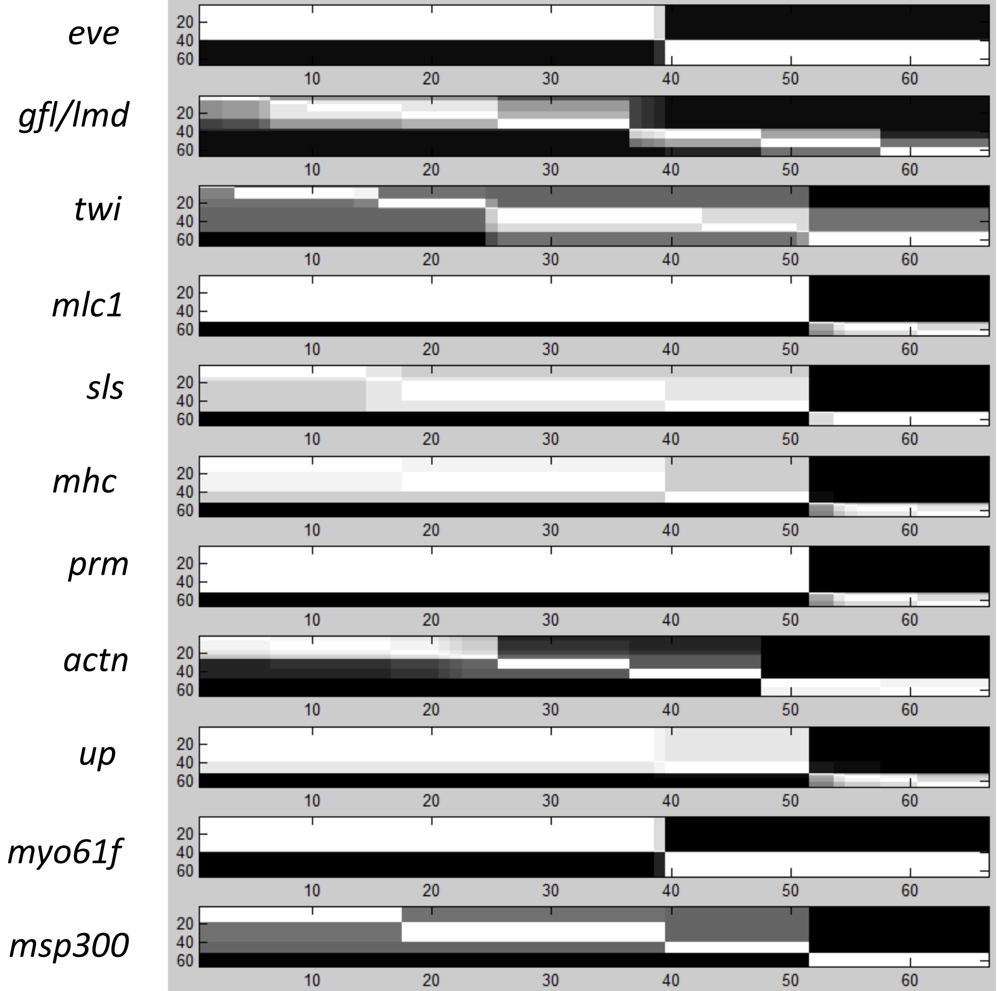

* The grey shading is used to indicate the connectivity probabilities between time points (black=0 and white=1)

# nhDBN on Drosophila muscle-related discrete gene expression dataset: k = 2; p = 1e-1

Connectivity probabilities between genes

	eve	gfl	twi	mlc1	sls	mhc	prm	actn	up	myo61f	msp300
eve	0.35	0	0	0.4	0.05	0	0.05	0.15	0	0.1	0
gfl/lmd	0	0	0	0.05	0.7	0.05	0.05	0.35	0	0.45	0
twi	0.6	1	1	0.45	0.7	0.95	0.6	0.7	0.5	0.15	1
mlc1	0.15	0.25	0.15	0.2	0.1	0	0	0.1	1	0.2	0
sls	0.15	0.1	0.15	0	0.05	0.05	0	0.15	0.05	0	0
mhc	0.25	0.4	0	0.5	0.05	0.05	0.25	0.05	0.1	0.15	0.55
prm	0.05	0.2	0.35	0.05	0	0	0	0.2	0.05	0.7	0.05
actn	0.9	0.2	0.85	0	0.15	0	0.05	1	0	0	0
up	0	0.45	0.2	0.2	0.15	0.7	0	0.15	0.25	0.1	1
myo61f	0.4	0.2	0.2	0.05	1	0.2	1	0.05	0	0.95	0
msp300	0.15	0.2	0.1	1	0.05	1	1	0.1	1	0.05	0.4

* The color yellow marks the edge whose probability is bigger than 0.5

Connectivity probabilities between time points

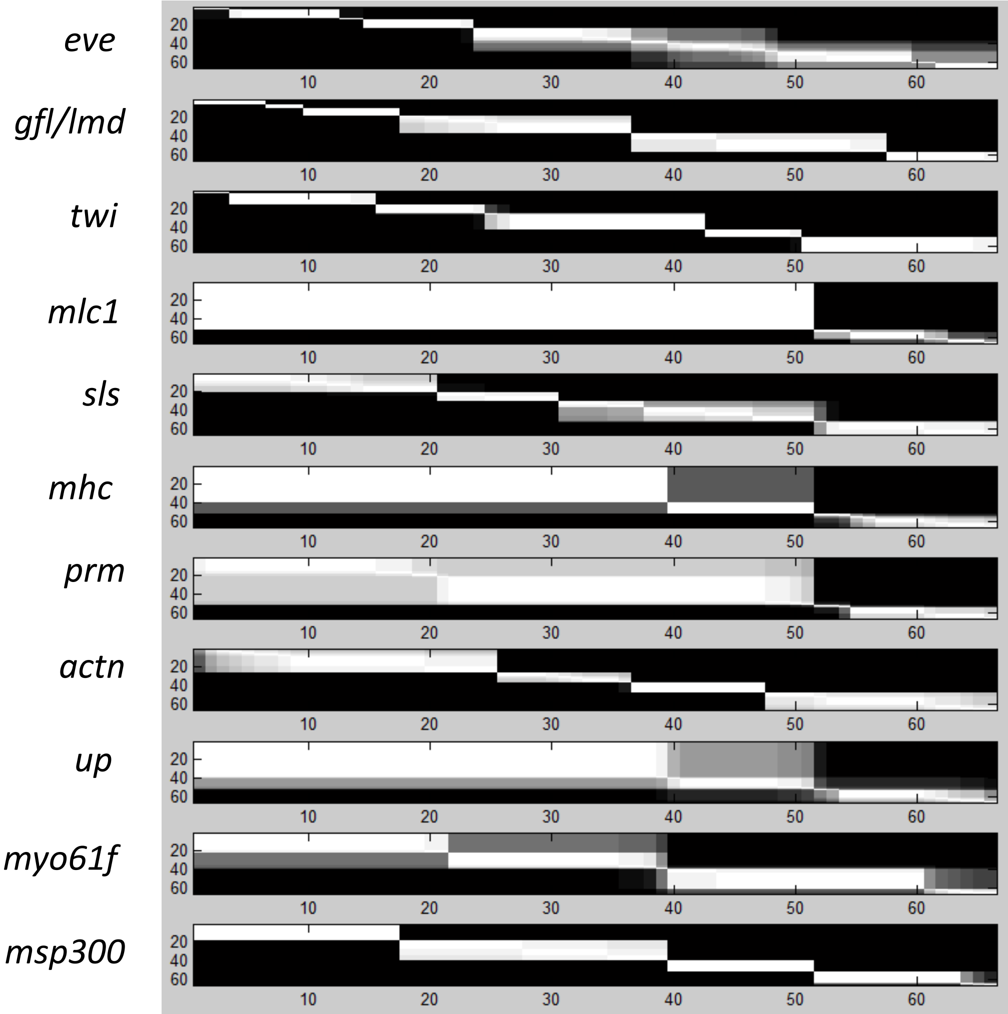

* The grey shading is used to indicate the connectivity probabilities between time points (black=0 and white=1)

# nhDBN on Drosophila muscle-related discrete gene expression dataset: k = 2; p = 2e-1

Connectivity probabilities between genes

	eve	gfl	twi	mlc1	sls	mhc	prm	actn	up	myo61f	msp300
eve	0.25	0.05	0	0.85	0	0	0.1	0.05	0.05	0	0
gfl/lmd	0.2	0.05	0	0	0.05	0.1	0	0.35	0	0.45	0.05
twi	0.7	1	1	0.15	0.95	0.35	0.55	0.6	0.95	0.45	0.95
mlc1	0.15	0.4	0.15	0.15	0.15	0	0.05	0.15	0.95	0.05	0
sls	0.15	0	0.05	0	0	0	0.05	0.15	0	0.05	0
mhc	0.25	0.2	0.15	0.4	0.5	0	0.15	0.05	0	0.25	0.1
prm	0.1	0.1	0.35	0.05	0.1	0	0.1	0.1	0.05	0.35	0
actn	0.9	0.5	0.55	0.1	0.05	0.5	0	1	0	0.15	0.05
up	0	0.4	0.25	0.25	0.05	0.25	0	0.3	0	0.2	1
myo61f	0.2	0.25	0.4	0.05	1	0.8	1	0.1	0	0.85	0.45
msp300	0.1	0.05	0.1	1	0.15	1	1	0.15	1	0.15	0.4

* The color yellow marks the edge whose probability is bigger than 0.5

Connectivity probabilities between time points

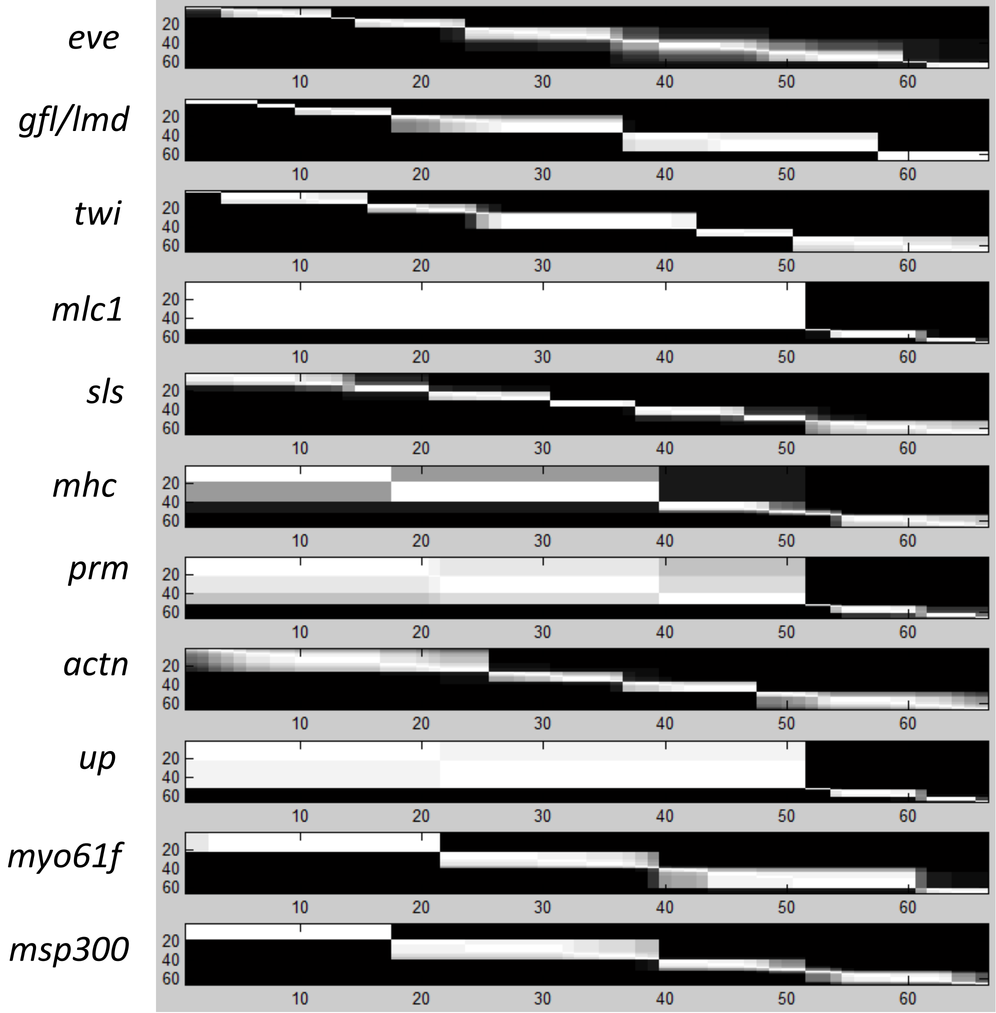

* The grey shading is used to indicate the connectivity probabilities between time points (black=0 and white=1)

# nhDBN on Drosophila muscle-related discrete gene expression dataset: k = 2; p = 5e-1

Connectivity probabilities between genes

	eve	*gfl*	*twi*	*mlc1*	*sls*	*mhc*	*prm*	*actn*	*up*	*myo61f*	*msp300*
eve	0	0	0	1	0	0.1	0.15	0	0	0.05	0.1
gfl/lmd	0.1	0	0	0	0.5	0.1	0	0.25	0.05	0.15	0
twi	0.95	1	1	0.05	0.95	0.85	0.75	0.7	0.9	0.15	0.85
mlc1	0	0.15	0.2	0.2	0.1	0.2	0.1	0.05	0.9	0.15	0
sls	0.45	0.1	0	0	0	0	0	0.1	0	0.3	0
mhc	0	0.35	0.2	0.35	0.2	0.05	0.25	0.05	0	0.1	0.05
prm	0.05	0.2	0.2	0	0	0.05	0	0	0.2	0.95	0.05
actn	1	0.65	0.05	0	0.1	0.05	0	0.9	0	0.15	0.05
up	0	0.2	0.2	0.45	0	0.55	0	0.05	0.1	0.1	1
myo61f	0.45	0.25	0.95	0	0.95	0.3	0.95	0.8	0.05	0.9	0.05
msp300	0	0.1	0.2	0.95	0.2	0.75	0.8	0.1	0.8	0	0.85

* The color yellow marks the edge whose probability is bigger than 0.5

Connectivity probabilities between time points

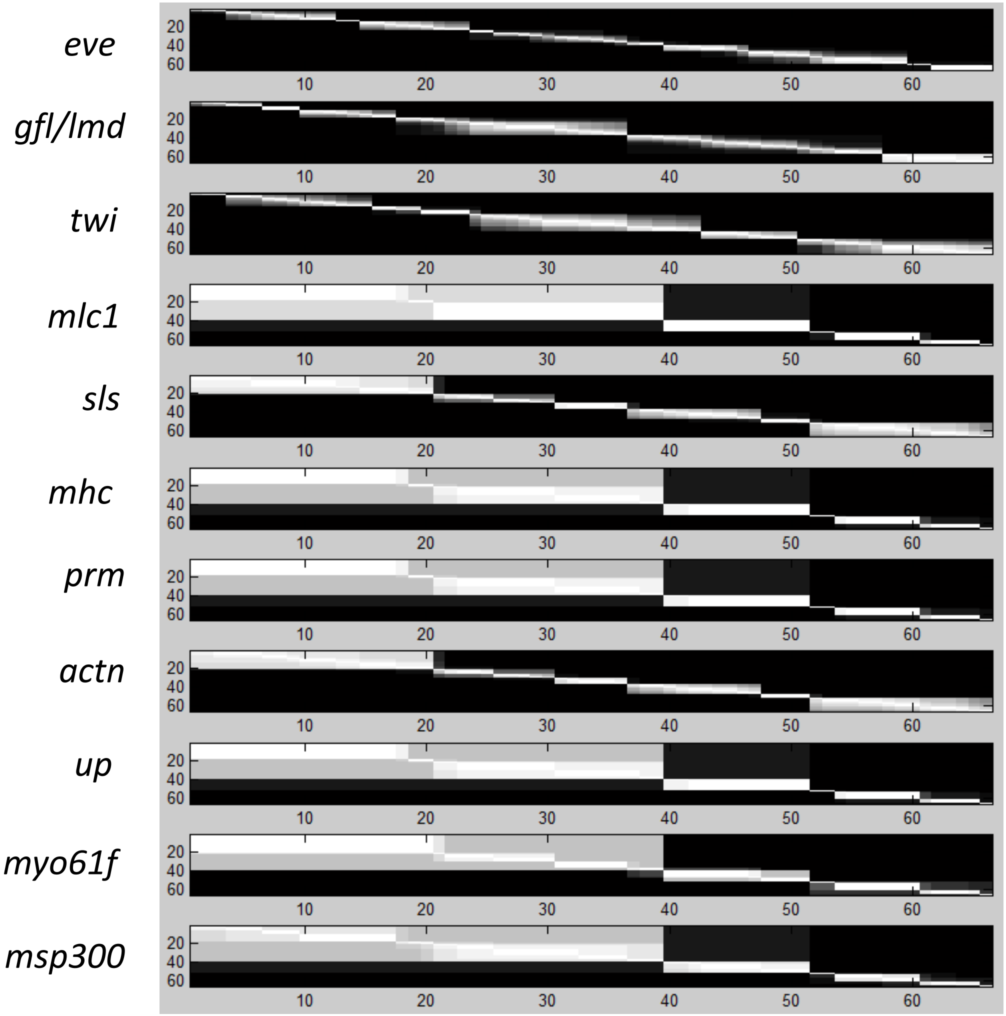

* The grey shading is used to indicate the connectivity probabilities between time points (black=0 and white=1)

# nhDBN on Drosophila muscle-related discrete gene expression dataset: k = 2; p = 8e-1

Connectivity probabilities between genes

	eve	gfl	twi	mlc1	sls	mhc	prm	actn	up	myo61f	msp300
eve	0.25	0.1	0.05	0.3	0.05	0	0.1	0.1	0	0.1	0
gfl/lmd	0.15	0.75	0.2	0.25	0.05	0	0.05	0	0.05	0.1	0.05
twi	0.85	0.6	1	0.3	0.9	0.85	0.55	0.85	0.65	0.3	0.9
mlc1	0.1	0.15	0.1	0.35	0.1	0	0	0	0.75	0.1	0.05
sls	0.25	0.1	0.2	0	0.25	0	0	0.05	0	0.25	0
mhc	0.05	0.25	0.05	0.45	0	0.05	0.15	0.15	0.1	0.1	0.05
prm	0.05	0.15	0.1	0.1	0.1	0	0	0.15	0.25	0.85	0
actn	0.8	0.4	0.25	0	0.3	0.05	0	1	0.05	0.05	0.05
up	0.15	0.25	0.35	0.2	0.1	0.85	0.2	0.2	0.1	0.2	0.95
myo61f	0.3	0.1	0.5	0.05	0.85	0.25	0.95	0.35	0.1	0.9	0.15
msp300	0.05	0.15	0.1	1	0.3	0.95	0.95	0.15	0.9	0	0.8

* The color yellow marks the edge whose probability is bigger than 0.5

Connectivity probabilities between time points

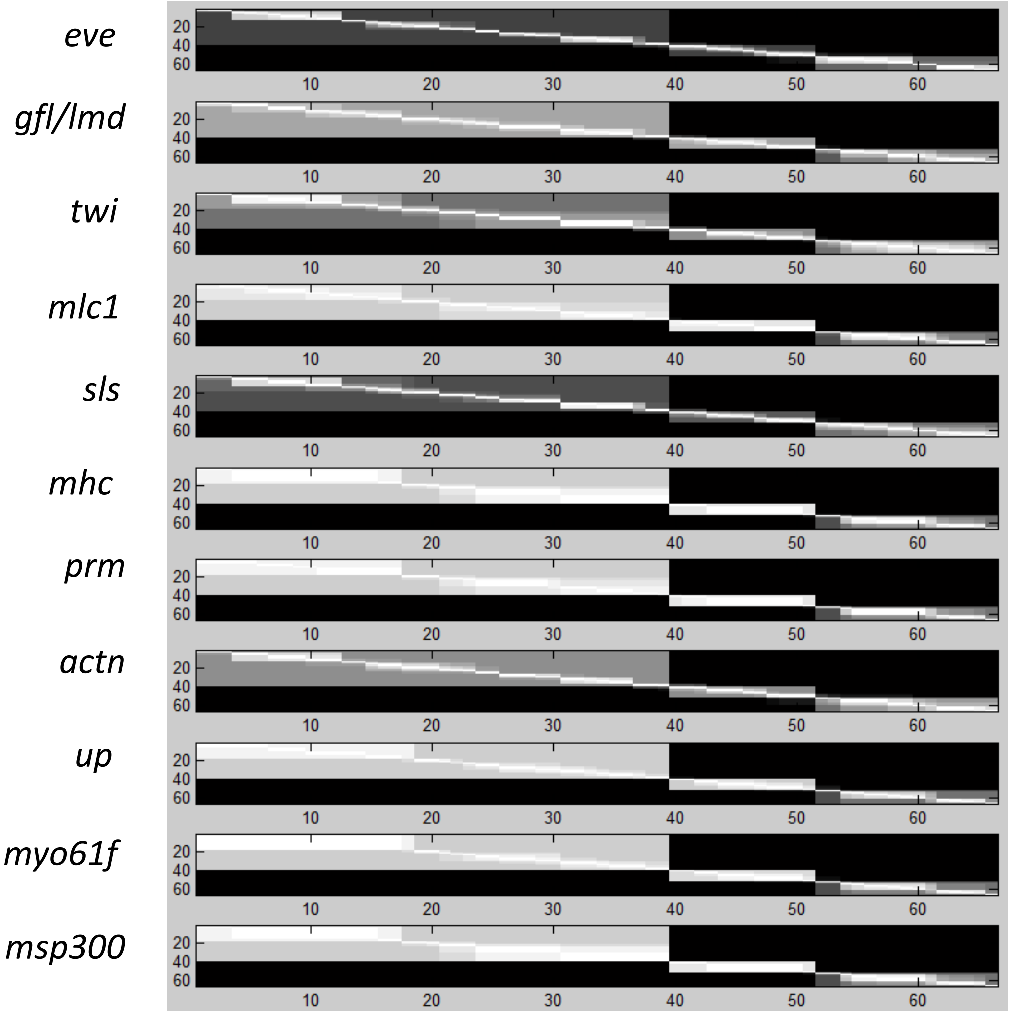

* The grey shading is used to indicate the connectivity probabilities between time points (black=0 and white=1)

# nhDBN on Drosophila muscle-related discrete gene expression dataset: k = 2; p = 1

Connectivity probabilities between genes

	eve	gfl	twi	mlc1	sls	mhc	prm	actn	up	myo61f	msp300
eve	0.45	0.1	0.55	0	0	0	0.05	0.05	0	0.1	0
gfl/lmd	0	0.8	0.25	0	0.15	0	0	0	0	0	0
twi	1	1	1	0.95	0.75	0.9	0.65	0.95	1	0.85	1
mlc1	0.2	0.2	0	0.3	0.1	0.1	0.2	0.1	0.35	0.45	0.35
sls	0.2	0.1	0.2	0	0.05	0	0.05	0	0.05	0.3	0
mhc	0	0.05	0	0.4	0.2	0.25	0.15	0.2	0.1	0.1	0.05
prm	0.05	0.15	0	0.2	0	0.2	0.1	0.1	0.25	0.3	0.15
actn	1	0.35	0.8	0.25	0.35	0.3	0.55	1	0.1	0.5	0.25
up	0.05	0.15	0	0.3	0.15	0.3	0.15	0.15	0.2	0	0.9
myo61f	0	0	0.05	0.4	0.95	0.45	1	0.1	0.45	0.2	0.15
msp300	0.05	0.1	0.15	0.2	0.3	0.5	0.1	0.35	0.5	0.2	0.15

* The color yellow marks the edge whose probability is bigger than 0.5

Connectivity probabilities between time points

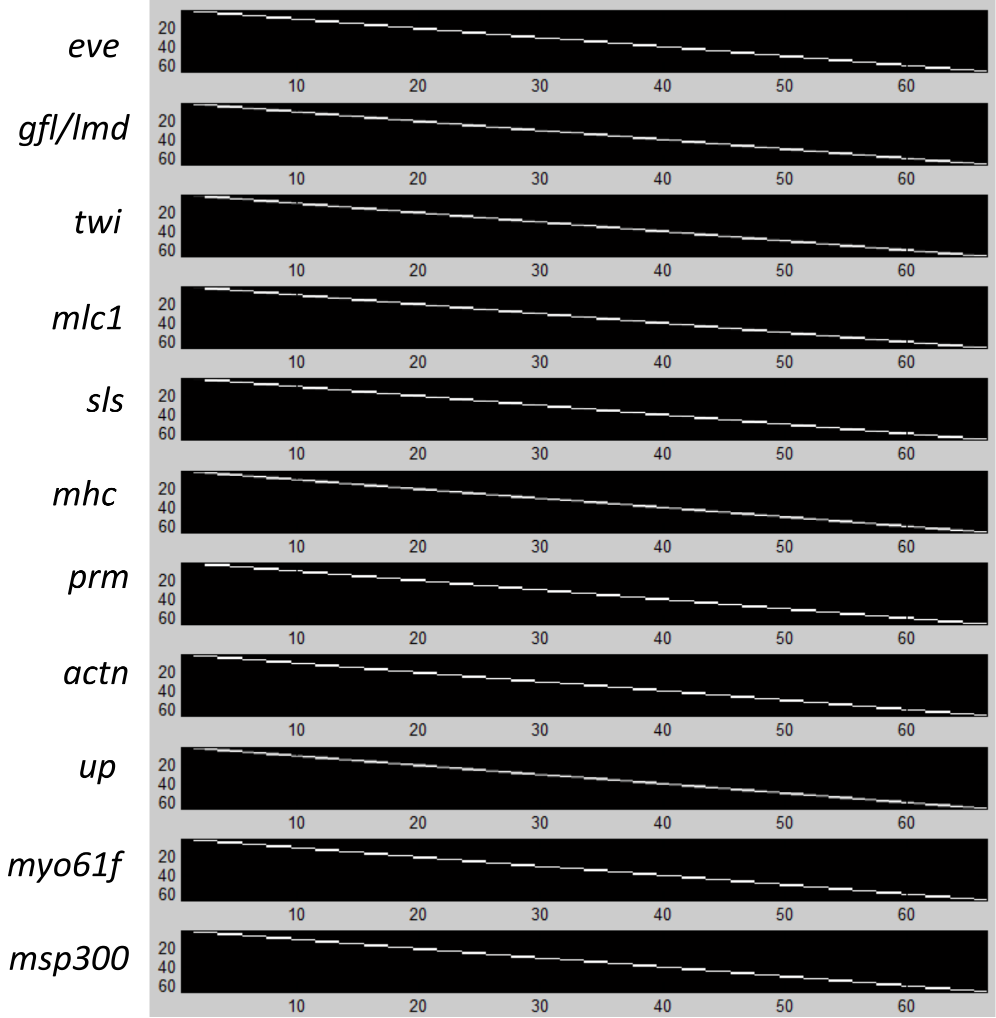

* The grey shading is used to indicate the connectivity probabilities between time points (black=0 and white=1)

# nhDBN on Drosophila muscle-related continuous gene expression dataset: k = 2; p = 1e-6

Connectivity probabilities between genes

	eve	gfl	twi	mlc1	sls	mhc	prm	actn	up	myo61f	msp300
eve	1	0.15	0.5	0	0.25	0.5	0.45	0.8	0.25	0	0.05
gfl/lmd	0.2	0.5	0	0	0.5	0	0	0.05	0.05	0	0.05
twi	0.05	0.3	0.85	0.05	0	0	0.05	0.15	0	0.1	0
mlc1	0.4	0.05	0.55	0	0.25	0.25	0	0.05	0.05	0.2	0.55
sls	0.5	0.05	0	0	0.5	0	0.15	0.05	0.05	0	0
mhc	0	0.5	0.1	0.95	0.1	1	0.9	0.15	0.9	0.05	0.95
prm	0.05	0.4	0.15	0.2	0.55	0	0.5	0.35	0.1	0.1	0
actn	0.25	0.2	0.4	1	0.7	1	0.8	1	1	0.85	1
up	0.1	0.15	0.25	0.75	0.15	0.2	0.1	0.15	0.55	0.65	0.25
myo61f	0.45	0.55	0	0.05	0	0.05	0	0.05	0.05	1	0
msp300	0	0.05	0.15	0	0	0	0.05	0.05	0	0.05	0.15

* The color yellow marks the edge whose probability is bigger than 0.5

Connectivity probabilities between time points

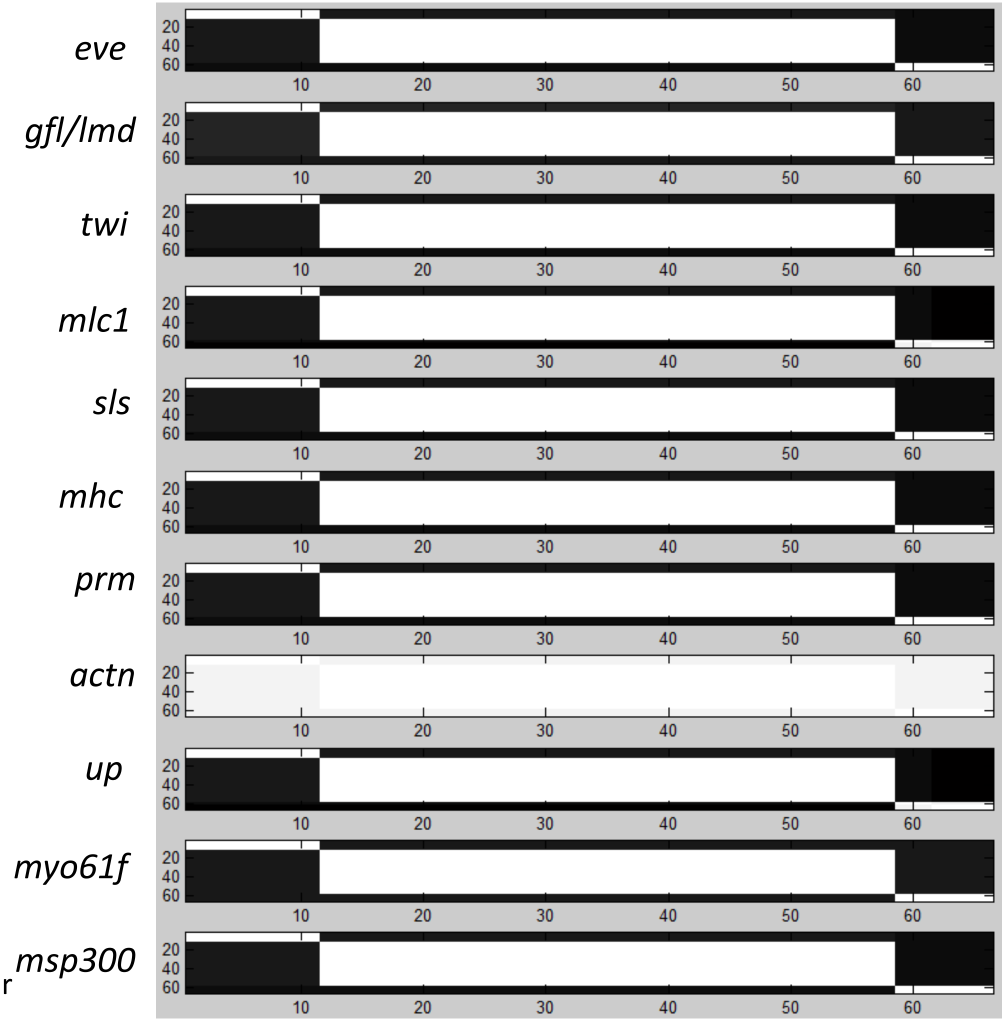

* The grey shading is used to indicate the connectivity probabilities between time points (black=0 and white=1)

# nhDBN on Drosophila muscle-related continuous gene expression dataset:
k = 2; p = 1e-5

Connectivity probabilities between genes

	eve	gfl	twi	mlc1	sls	mhc	prm	actn	up	myo61f	msp300
eve	1	0.25	0.8	0	0.25	0.1	0.2	0.65	0.3	0.15	0.1
gfl/lmd	0	0.95	0.1	0	0.6	0.05	0.1	0.15	0	0	0.05
twi	0	0.15	0.45	0	0.1	0	0.05	0.1	0	0.55	0
mlc1	0.6	0	0.65	0.2	0.15	0.3	0.2	0.1	0	0.05	0.35
sls	0.5	0	0	0	0.05	0.1	0.05	0.1	0.15	0	0
mhc	0.2	0.85	0.15	1	0.55	1	0.5	0.1	1	0.15	0.75
prm	0.05	0.15	0.25	0.2	0.35	0.1	0.7	0.15	0.25	0.15	0.2
actn	0.15	0.1	0.45	1	0.55	1	0.75	1	1	0.45	0.95
up	0.1	0.5	0.05	0.45	0.2	0.35	0.1	0.15	0.3	0.7	0.15
myo61f	0.05	0.05	0	0.15	0.1	0	0.1	0.25	0	0.75	0
msp300	0.25	0	0.1	0	0	0	0.1	0.05	0	0	0.45

* The color yellow marks the edge whose probability is bigger than 0.5

Connectivity probabilities between time points

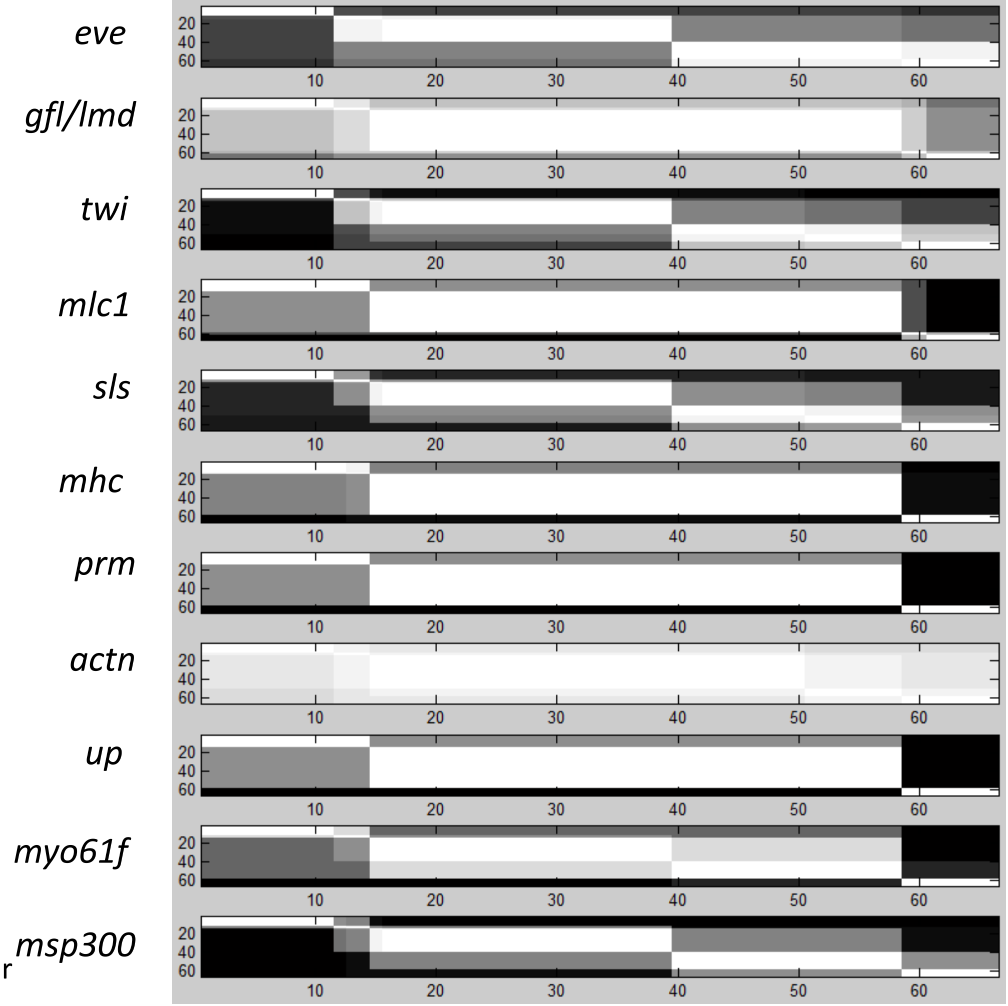

* The grey shading is used to indicate the connectivity probabilities between time points (black=0 and white=1)

# nhDBN on Drosophila muscle-related continuous gene expression dataset: k = 2; p = 1e-4

Connectivity probabilities between genes

	eve	*gfl*	*twi*	*mlc1*	*sls*	*mhc*	*prm*	*actn*	*up*	*myo61f*	*msp300*
eve	1	0.2	0.8	0.05	0.1	0.45	0.5	0.7	0.2	0.15	0
gfl/lmd	0.3	0.75	0	0	0.75	0.05	0	0.05	0	0	0
twi	0.05	0.05	0.6	0	0	0	0	0.25	0	0.1	0.05
mlc1	0.45	0	0.85	0.35	0.1	0.3	0	0.1	0	0.3	0.6
sls	0.55	0.05	0	0	0.3	0.05	0.05	0.1	0	0	0
mhc	0.05	0.7	0	1	0.15	1	0.95	0.15	1	0.05	1
prm	0	0.2	0.3	0.35	0.7	0	0.4	0.45	0.2	0.1	0
actn	0.05	0.05	0.25	1	0.85	1	0.85	1	1	0.75	1
up	0	0.45	0.1	0.25	0.05	0.15	0	0.05	0.55	0.55	0.15
myo61f	0.5	0.5	0	0	0	0	0.05	0	0.05	1	0.1
msp300	0	0	0.1	0	0	0	0.1	0.15	0	0	0.1

* The color yellow marks the edge whose probability is bigger than 0.5

Connectivity probabilities between time points

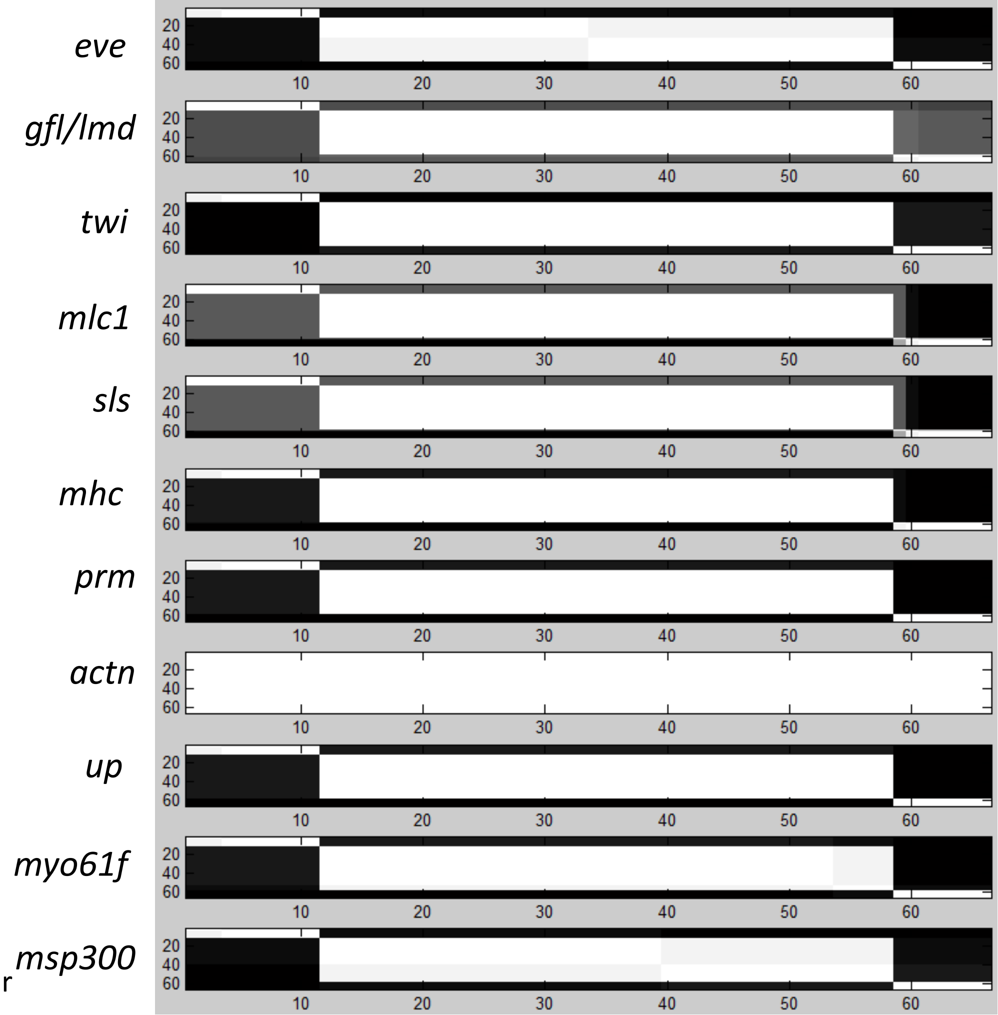

* The grey shading is used to indicate the connectivity probabilities between time points (black=0 and white=1)

# nhDBN on Drosophila muscle-related continuous gene expression dataset: k = 2; p = 1e-3

Connectivity probabilities between genes

	eve	*gfl*	*twi*	*mlc1*	*sls*	*mhc*	*prm*	*actn*	*up*	*myo61f*	*msp300*
eve	0.95	0.15	0.55	0	0.5	0.1	0.15	0.65	0.8	0.05	0.1
gfl/lmd	0.05	0.45	0.4	0	0.2	0	0	0	0	0	0.05
twi	0	0.05	0.65	0	0.15	0	0	0.05	0	0.9	0
mlc1	0.7	0.15	0.55	0	0.2	0.6	0.1	0.15	0	0.15	0.65
sls	0.45	0	0	0	0.15	0.05	0.05	0	0	0	0.05
mhc	0.05	0.8	0.25	1	0.35	1	0.85	0.5	1	0.1	0.95
prm	0.4	0.05	0.05	0.35	0.5	0	0.75	0.1	0.05	0.15	0.05
actn	0	0	0.35	1	0.5	1	0.85	1	1	0.1	1
up	0.1	0.7	0.2	0.6	0.2	0.2	0	0.3	0.1	0.7	0
myo61f	0	0.6	0	0	0.2	0.05	0	0.1	0	0.85	0
msp300	0.2	0	0	0.05	0.05	0	0.15	0.05	0	0	0.15

* The color yellow marks the edge whose probability is bigger than 0.5

Connectivity probabilities between time points

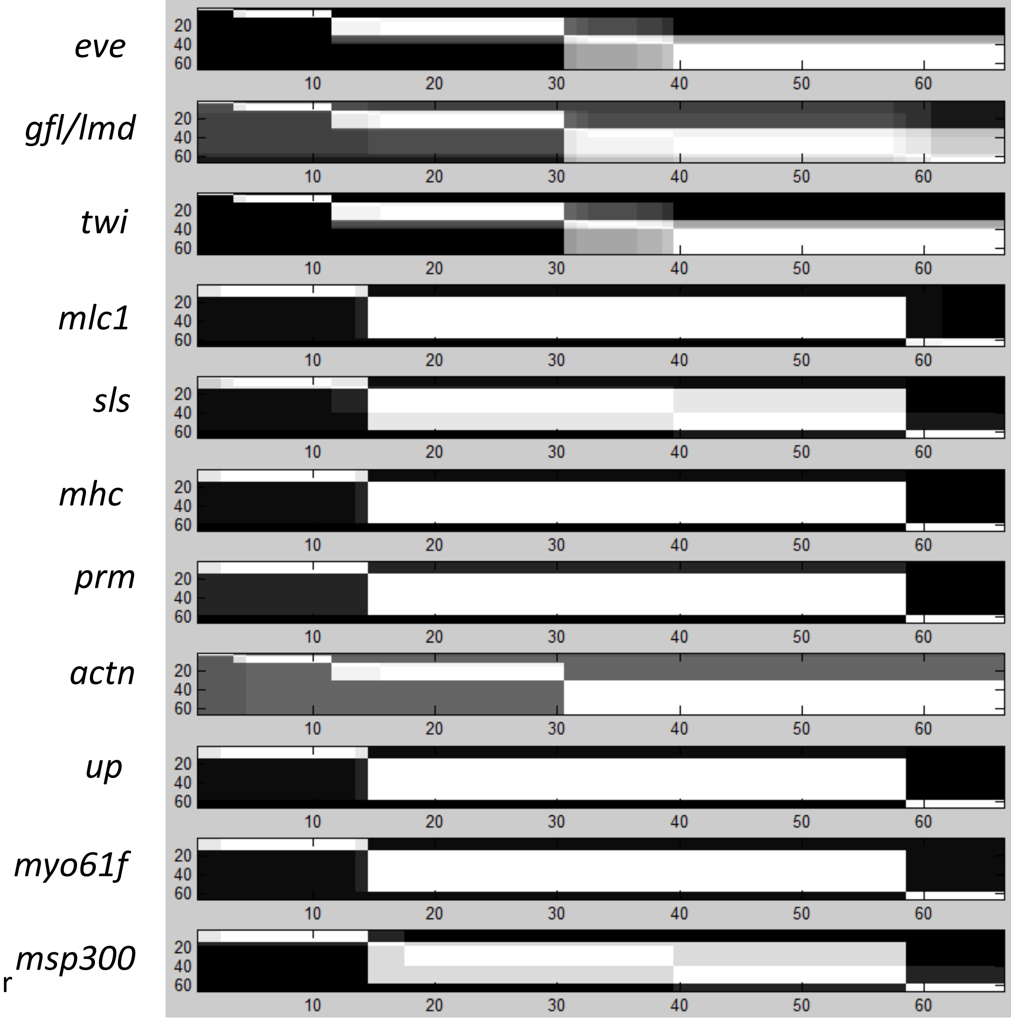

* The grey shading is used to indicate the connectivity probabilities between time points (black=0 and white=1)

# nhDBN on Drosophila muscle-related continuous gene expression dataset: k = 2; p = 1e-2

Connectivity probabilities between genes

	eve	gfl	twi	mlc1	sls	mhc	prm	actn	up	myo61f	msp300
eve	1	0.15	0.9	0.5	0.1	0.05	0	0.35	0.1	0	0
gfl/lmd	0.4	0.1	0.05	0	0.7	0	0	0	0.1	0.1	0
twi	0	0.2	0	0	0.25	0.05	0.05	0	0	0.9	0
mlc1	0.2	0.4	0.2	0.1	0.45	0.35	0.1	1	0.05	0.15	0.8
sls	0.2	0	0	0	0.05	0	0.05	0	0.1	0	0
mhc	0.05	0.55	0.55	0.95	0.55	1	1	0.85	1	0.05	1
prm	0.15	0.35	0	0.2	0.2	0.35	0.9	0.1	0.05	0.05	0
actn	0.1	0.4	1	1	0.4	1	0.85	0.55	0.85	0.3	1
up	0.5	0.5	0.3	0.25	0.15	0.2	0	0	0.75	0.8	0.15
myo61f	0.15	0.3	0	0	0.05	0	0	0.05	0	0.65	0
msp300	0.25	0	0	0	0.05	0	0.05	0.1	0	0	0.05

* The color yellow marks the edge whose probability is bigger than 0.5

Connectivity probabilities between time points

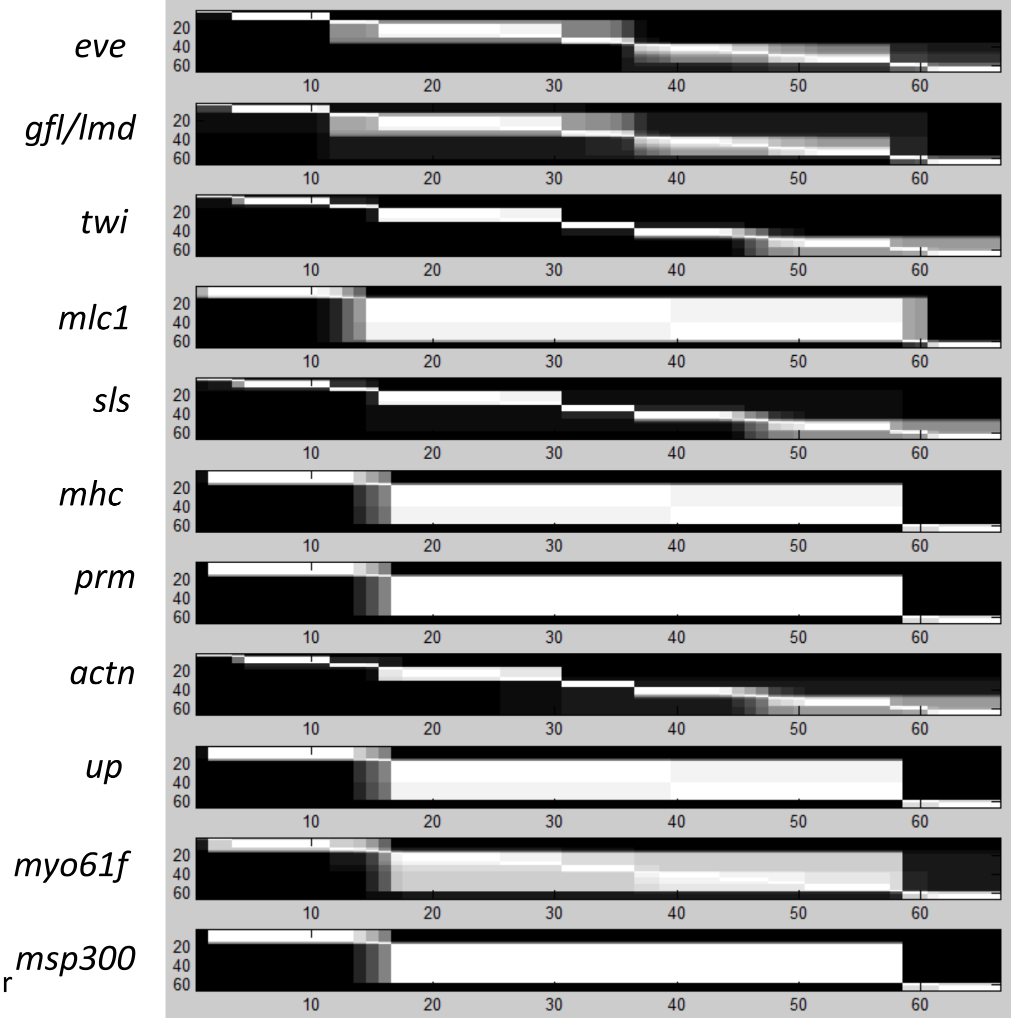

* The grey shading is used to indicate the connectivity probabilities between time points (black=0 and white=1)

# nhDBN on Drosophila muscle-related continuous gene expression dataset: k = 2; p = 1e-1

Connectivity probabilities between genes

	eve	gfl	twi	mlc1	sls	mhc	prm	actn	up	myo61f	msp300
eve	0.55	0	0	0	0.15	0.5	0.05	0.05	1	0	0
gfl/lmd	0	0.05	0.25	0.05	0.55	0	0	0	0	0	0.1
twi	0.45	0.1	0	0	0.05	0	0	0	0	0.05	0
mlc1	0.75	0.15	0.9	0.8	0.7	0.45	0.75	1	0	1	0.7
sls	0	0	0	0	0	0.05	0	0	0	0	0
mhc	0.05	0.8	0.7	1	0.3	1	1	0.9	1	0.45	1
prm	0.25	0.05	0	0	0.15	0	0	0.1	0	0.05	0.05
actn	0.1	0.9	1	1	0.7	1	1	0.95	1	1	1
up	0.65	0.15	0.15	0.1	0.15	0	0.1	0	0	0.4	0
myo61f	0.2	0.8	0	0.05	0.1	0	0	0	0	0	0
msp300	0	0	0	0	0.1	0	0.1	0	0	0.05	0.15

* The color yellow marks the edge whose probability is bigger than 0.5

Connectivity probabilities between time points

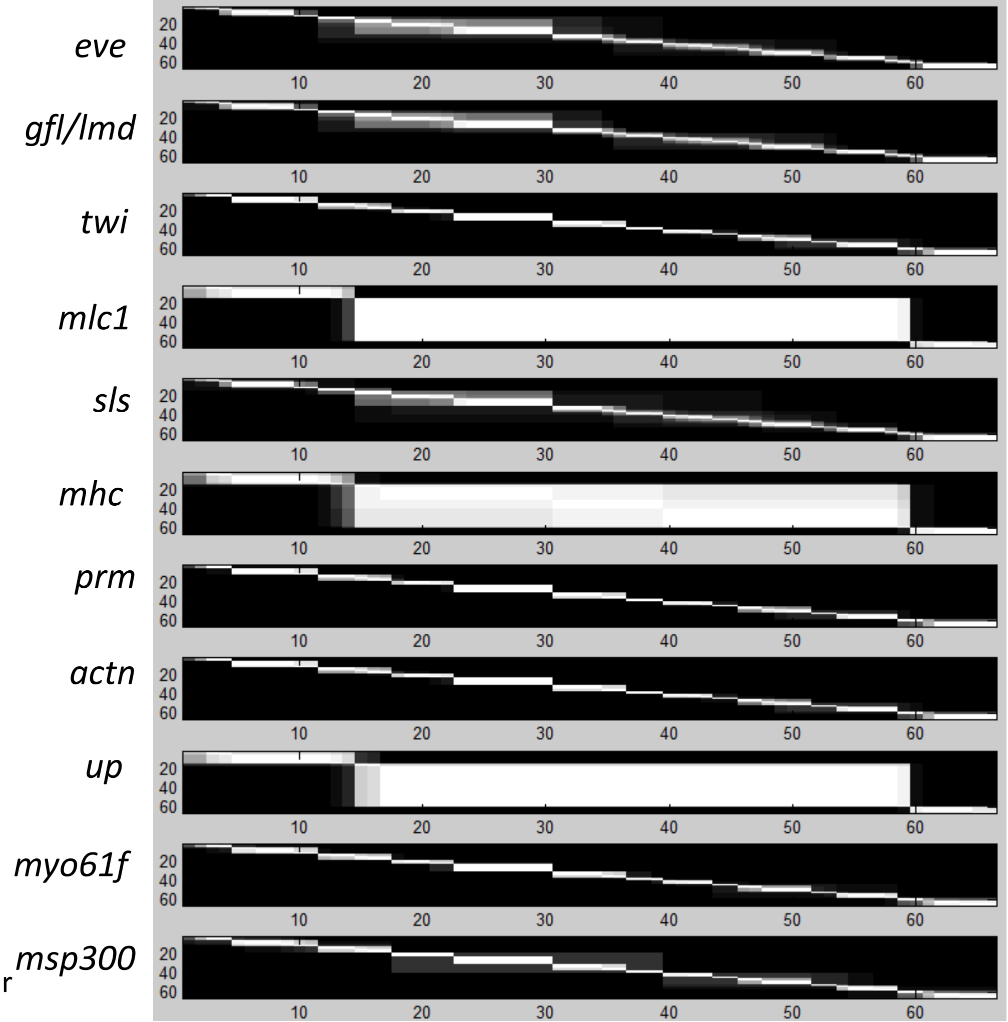

* The grey shading is used to indicate the connectivity probabilities between time points (black=0 and white=1)

# nhDBN on Drosophila muscle-related continuous gene expression dataset: k = 2; p = 2e-1

Connectivity probabilities between genes

	eve	gfl	twi	mlc1	sls	mhc	prm	actn	up	myo61f	msp300
eve	0.4	0.1	0.15	0.35	0.1	0	0	0.8	0	0	0
gfl/lmd	0.1	0.05	0	0	0.2	0	0.05	0	0	0.85	0
twi	0.4	0.05	0	0	0.05	0	0	0	0	0.1	0
mlc1	0.85	0.65	0.85	0.4	0.75	0.35	0.6	1	1	0.65	0.95
sls	0	0	0	0	0.15	0	0	0	0	0	0
mhc	0.4	0.8	1	1	0.5	1	1	1	1	0.1	1
prm	0.55	0.1	0	0	0.25	0	0.2	0	0	0	0
actn	0.25	1	1	1	0.55	1	1	0.2	1	0.9	1
up	0	0.2	0	0.25	0.1	0.2	0.05	0	0	0.4	0.05
myo61f	0.05	0.05	0	0	0.15	0	0	0	0	0	0
msp300	0	0	0	0	0.15	0.45	0.1	0	0	0	0

* The color yellow marks the edge whose probability is bigger than 0.5

Connectivity probabilities between time points

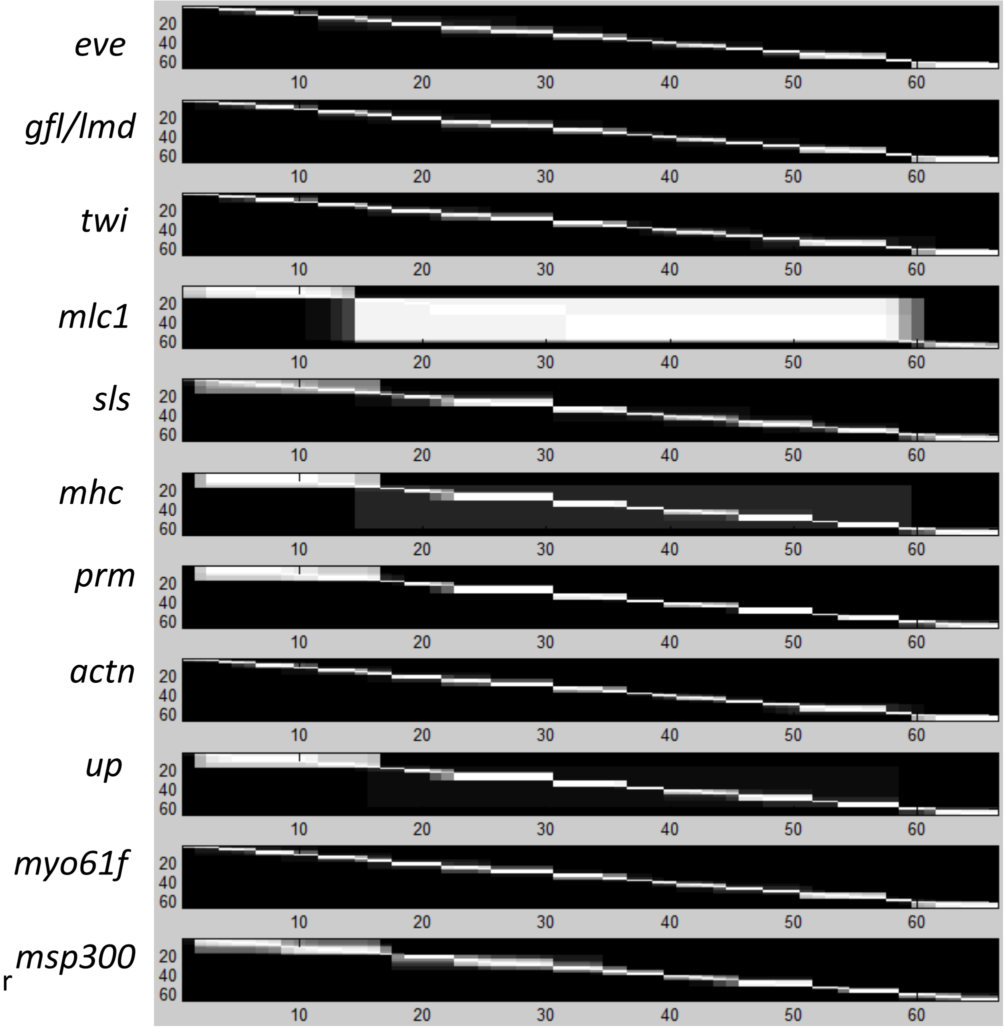

* The grey shading is used to indicate the connectivity probabilities between time points (black=0 and white=1)

# nhDBN on Drosophila muscle-related continuous gene expression dataset: k = 2; p = 5e-1

Connectivity probabilities between genes

	eve	gfl	twi	mlc1	sls	mhc	prm	actn	up	myo61f	msp300
eve	0.8	0.25	0	0	0.3	0	0	0.65	0	0	0
gfl/lmd	0.05	0.15	0	0	0.35	0	0	0.15	0	0	0
twi	0.1	0.05	0	0	0.05	0	0	0	0	0	0
mlc1	0.7	0.05	1	1	0.5	1	1	1	1	1	1
sls	0	0	0	0	0.15	0	0	0	0	0	0
mhc	0.45	0.9	0.9	0	0.4	1	1	0.5	0.85	0.75	1
prm	0.15	0.1	0.05	1	0.15	0	0	0	0.05	0.05	0
actn	0.65	1	1	0	0.55	1	1	0.7	0.7	1	1
up	0.1	0.2	0.05	1	0.3	0	0	0	0.25	0.2	0
myo61f	0	0.25	0	0	0	0	0	0	0	0	0
msp300	0	0.05	0	0	0.2	0	0	0	0.15	0	0

* The color yellow marks the edge whose probability is bigger than 0.5

Connectivity probabilities between time points

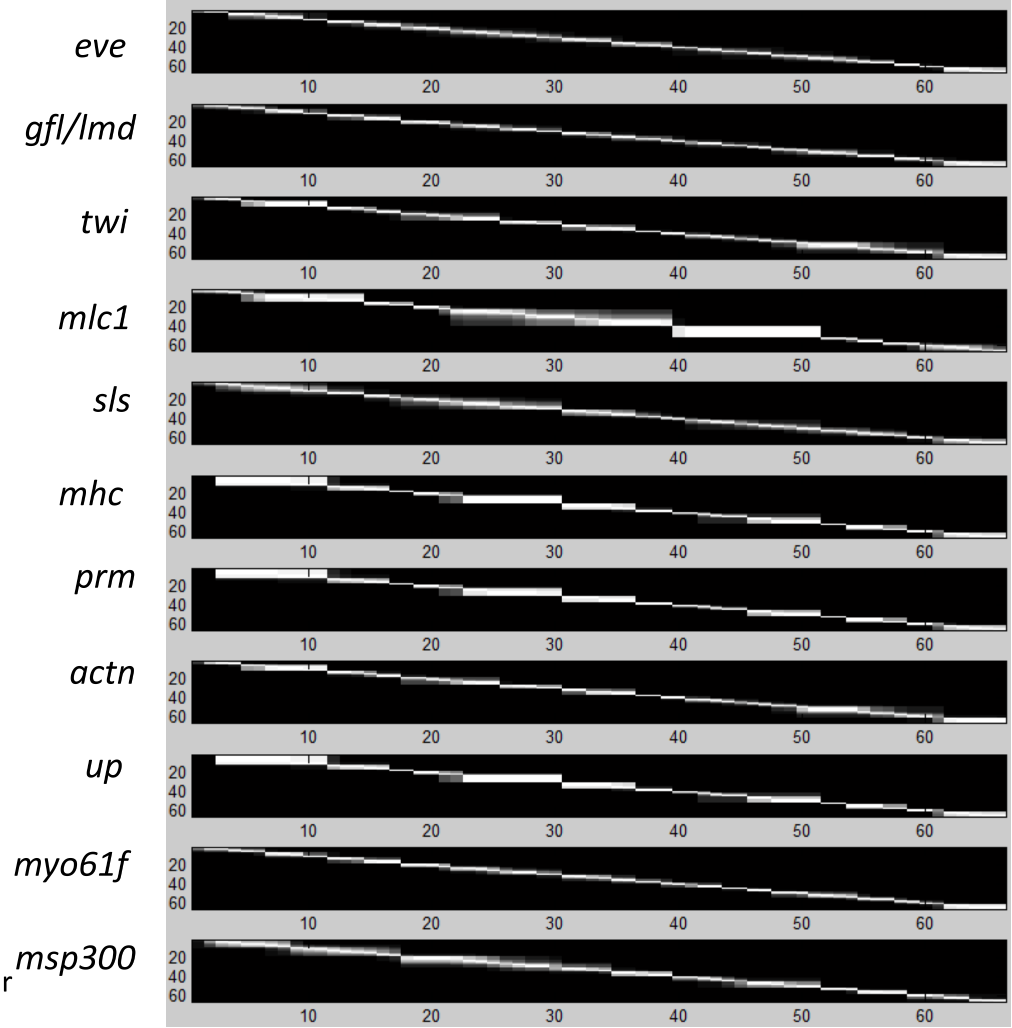

* The grey shading is used to indicate the connectivity probabilities between time points (black=0 and white=1)

# nhDBN on Drosophila muscle-related continuous gene expression dataset: k = 2; p = 8e-1

Connectivity probabilities between genes

	eve	gfl	twi	mlc1	sls	mhc	prm	actn	up	myo61f	msp300
eve	0.5	0.2	0	0	0.15	0	0.05	0.15	0	0	0
gfl/lmd	0.1	0.4	0	0	0.3	0	0	0.05	0	0	0
twi	0.2	0	0	0	0.15	0	0	0	0	0	0
mlc1	0.6	0.35	1	0.45	0.8	1	0.95	1	1	0.95	1
sls	0	0	0	0	0.1	0	0	0	0	0	0
mhc	0.8	0.9	0.9	1	0.4	1	1	0.85	1	0.85	1
prm	0.05	0	0	0	0.25	0	0	0.05	0	0	0
actn	0.55	1	1	0.7	0.4	1	0.9	0.9	0.65	1	1
up	0.15	0.1	0.1	0.85	0.15	0	0.1	0	0.25	0.2	0
myo61f	0.05	0.05	0	0	0.15	0	0	0	0	0	0
msp300	0	0	0	0	0.1	0	0	0	0.1	0	0

* The color yellow marks the edge whose probability is bigger than 0.5

Connectivity probabilities between time points

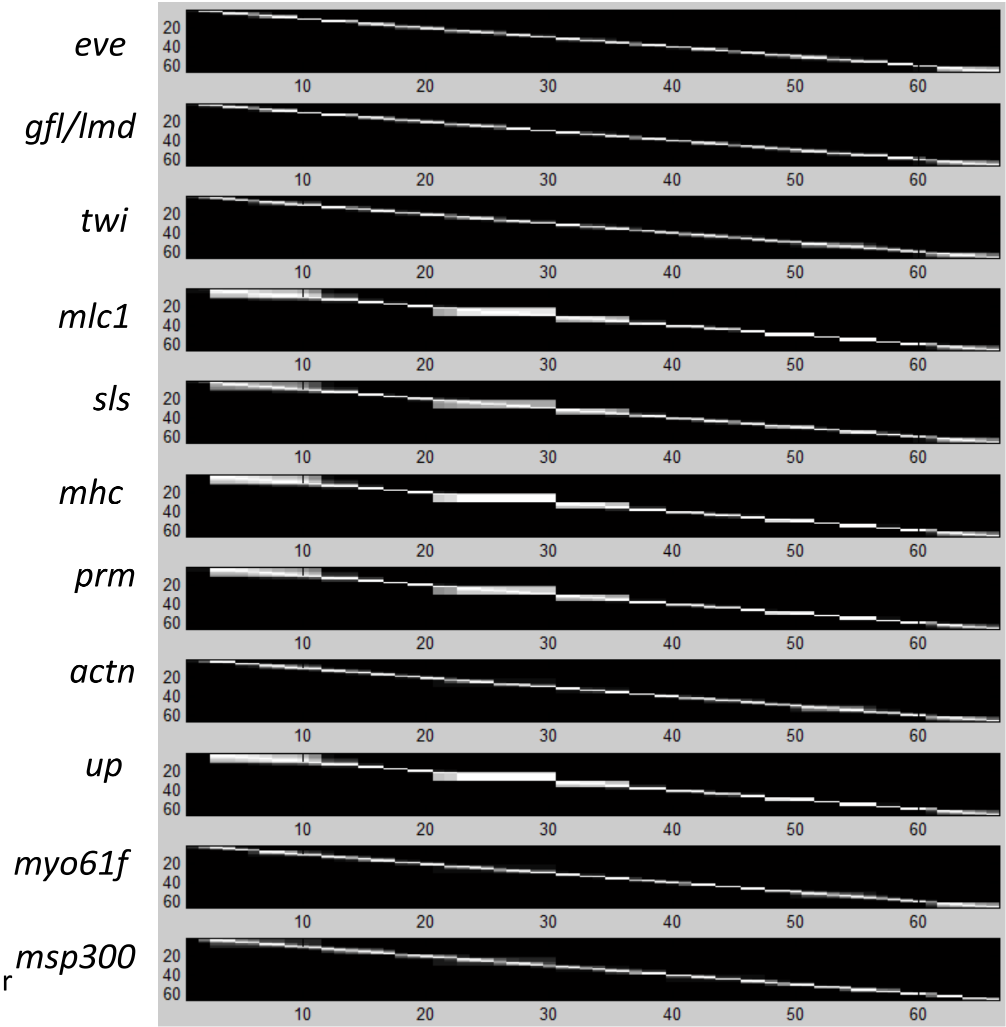

* The grey shading is used to indicate the connectivity probabilities between time points (black=0 and white=1)

# nhDBN on Drosophila muscle-related continuous gene expression dataset: k = 2; p = 1

Connectivity probabilities between genes

	eve	gfl	twi	mlc1	sls	mhc	prm	actn	up	myo61f	msp300
eve	0.4	0.05	0	0	0.25	0	0	0.15	0	0.05	0
gfl/lmd	0.15	0.1	0	0	0.05	0	0	0	0	0	0
twi	0	0.05	0	0	0.05	0	0	0	0	0	0
mlc1	0.55	0.25	1	1	0.65	1	1	1	1	0.9	1
sls	0	0	0	0	0	0	0	0	0	0	0
mhc	0.9	1	1	1	0.7	1	1	1	1	0.8	1
prm	0.05	0.1	0	0	0.2	0	0.7	0	0	0	0
actn	0.9	0.95	0.95	0	0.5	1	0.1	0.85	0	0.9	1
up	0	0.35	0.05	1	0.2	0	0.15	0	1	0.35	0
myo61f	0.05	0.05	0	0	0.2	0	0.05	0	0	0	0
msp300	0	0.05	0	0	0	0	0	0	0	0	0

* The color yellow marks the edge whose probability is bigger than 0.5

Connectivity probabilities between time points

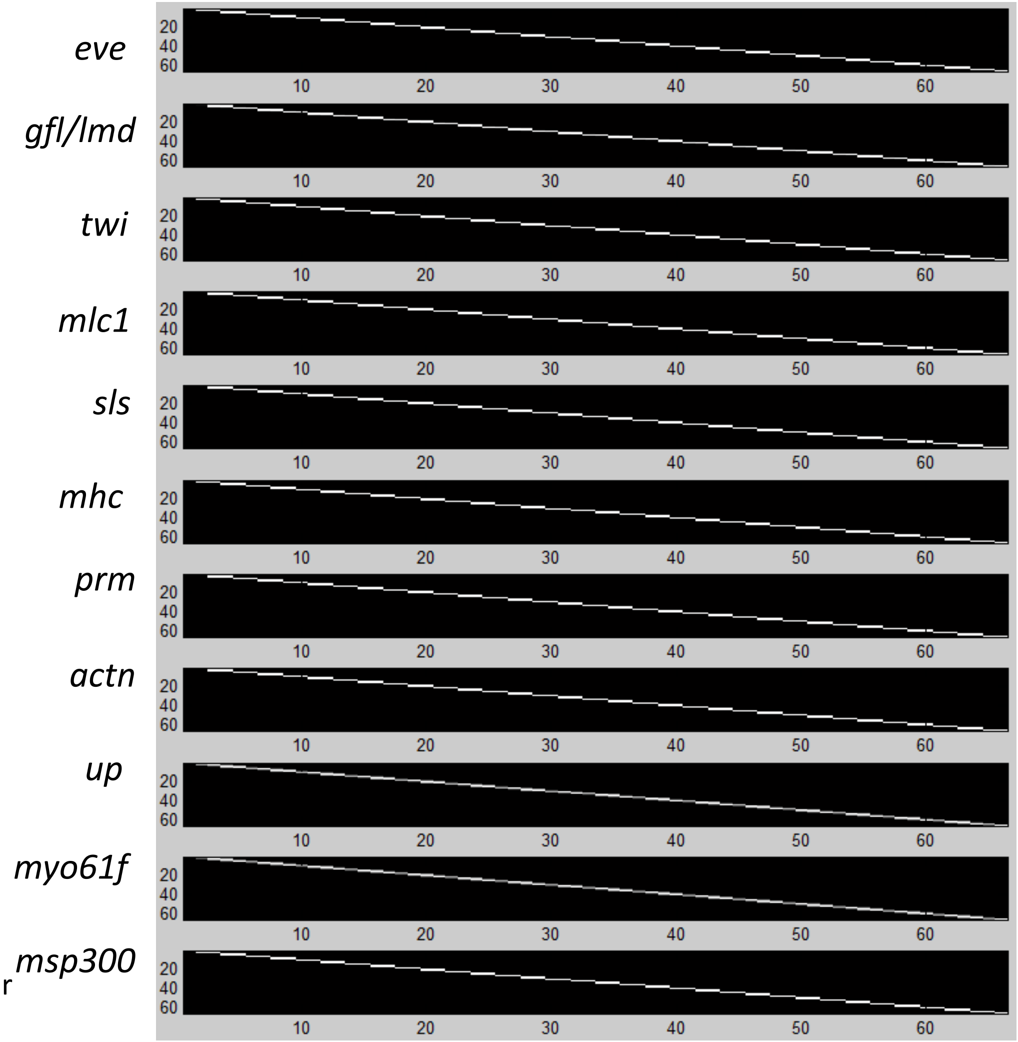

* The grey shading is used to indicate the connectivity probabilities between time points (black=0 and white=1)
